# Copper-Catalyzed Hydroamination of 2-Alkynylazobenzenes: Synthesis of 3-Alkenyl-2*H*-Indazoles

**DOI:** 10.1021/acs.joc.4c02144

**Published:** 2024-11-02

**Authors:** Clara Mañas, Juan Herrero-Bourdieu, Estíbaliz Merino

**Affiliations:** † Departamento de Química Orgánica y Química Inorgánica, Instituto de Investigación Andrés M. del Río (IQAR), Facultad de Farmacia, Universidad de Alcalá, Alcalá de Henares, 28805 Madrid, Spain; ‡ Instituto Ramón y Cajal de Investigación Sanitaria (IRYCIS), Ctra. De Colmenar Viejo, Km. 9.100, 28034 Madrid, Spain

## Abstract

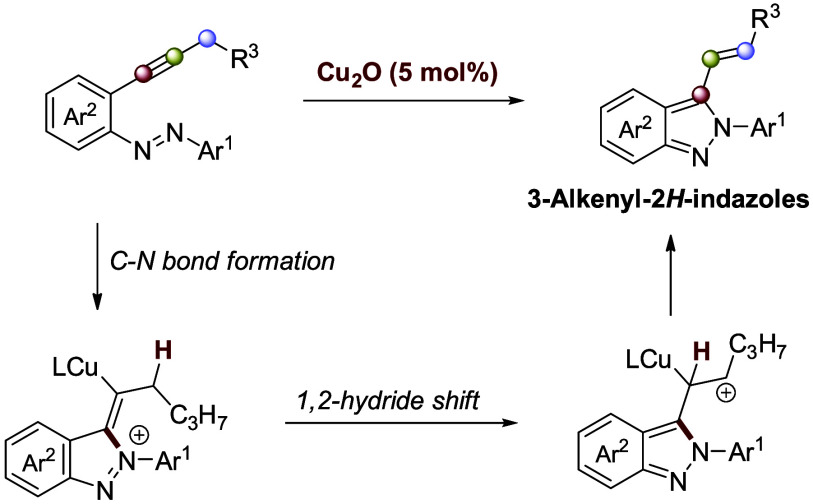

A copper-catalyzed intramolecular synthesis of 3-alkenyl-2*H*-indazoles from 2-alkynylazobenzenes is described. The reaction proceeds in a single step via C–N bond formation and a subsequent 1,2-hydride shift, affording products in high yields. DFT calculations suggest the 1,2-hydride shift as the rate-determining step. Further derivatization enables functionalization of the 3-alkenyl-2*H*-indazoles.

## Introduction

**Figure 1:**
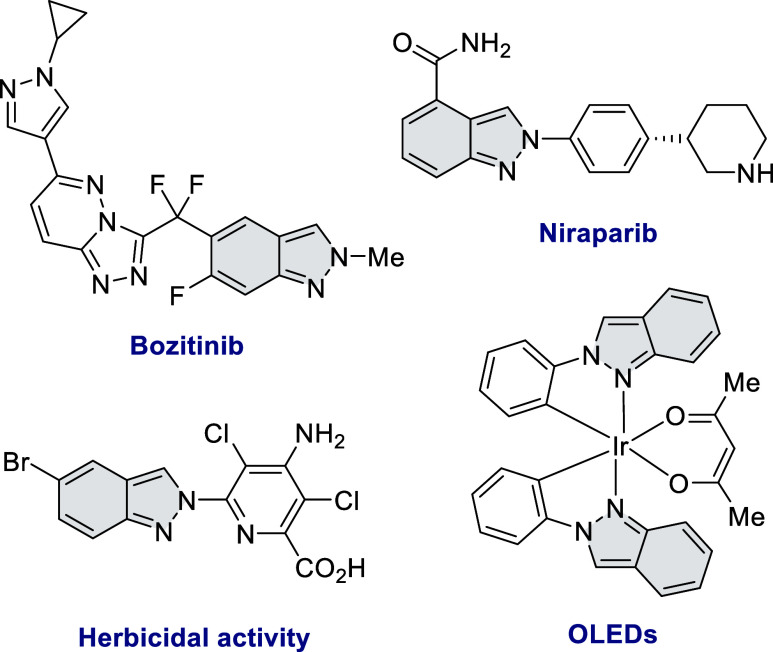
Examples of compounds containing a 2*H*-indazol scaffold.

2*H*-Indazoles, a class of heterocyclic compounds featuring a fused benzene and pyrazole ring, have emerged as attractive targets in medicinal chemistry due to their unique properties.^1−5^ Their diverse biological activities, structural flexibility, and favorable drug-like properties make them valuable candidates for drug discovery. Notably, 2*H*-indazoles exhibit a wide range of pharmacological effects, including anti-inflammatory, antiarrhythmic, antitumor, antifungal, antibacterial, and anti-HIV activities.^6−11^ Additionally, they have demonstrated herbicidal activity.^12^ Beyond medicinal applications, 2*H*-indazoles have found utility in other scientific fields (Figure 1). The indazole ligand serves as a nitrogen σ donor in organometallic chemistry.^13−16^ Their potential as charge-transporting materials makes them promising candidates for organic optoelectronics. For instance, iridium(III) complexes containing indazole ligands have been successfully employed in the fabrication of organic light-emitting diodes (OLEDs).^17−19^

**Scheme 1:**
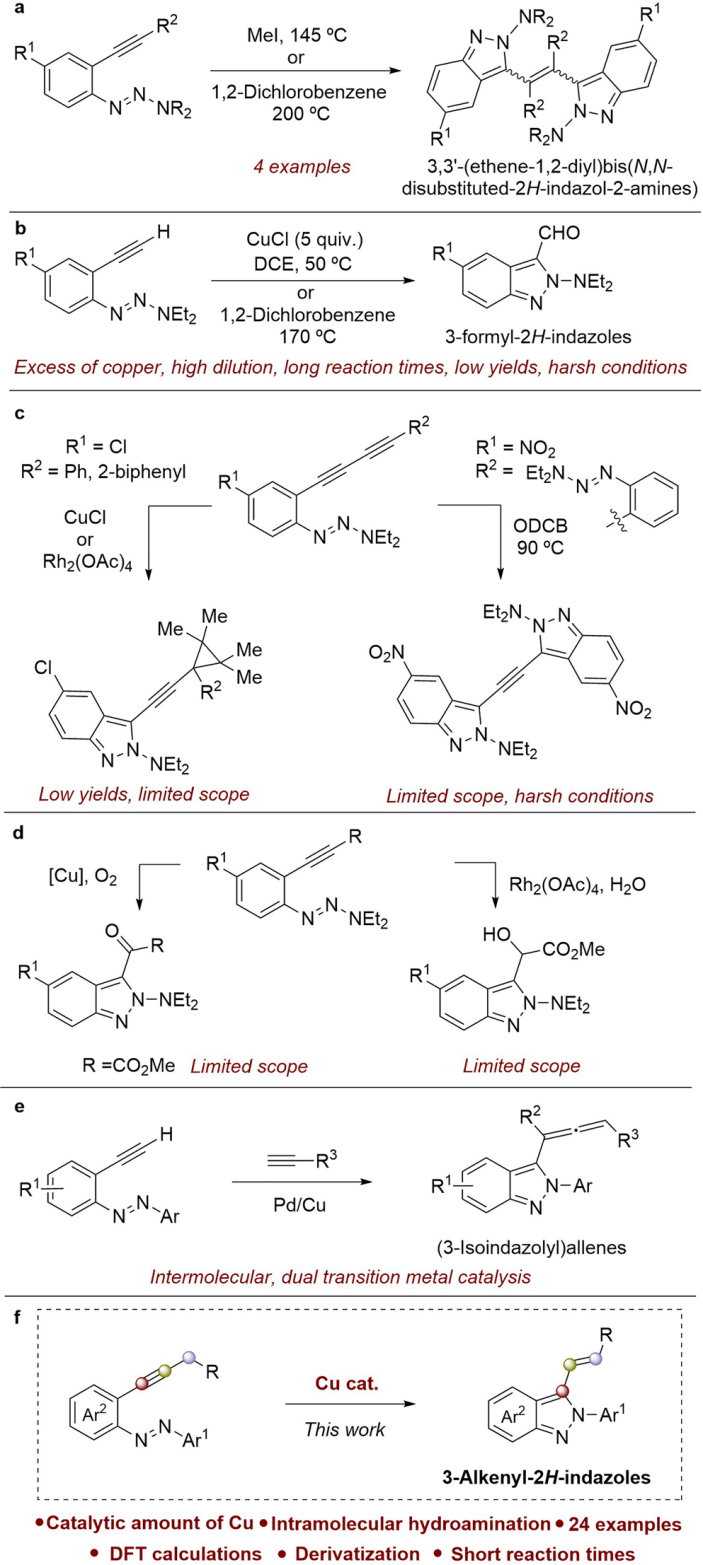
Intramolecular Cyclization of 2-Alkynylazobenzenes and Triazenes

To date, the synthesis of 2-alkenyl-2*H*-indazoles has been described using 1-(2-ethynylphenyl)-3,3-dialkyltriazenes as starting materials, and this route has been underexplored. Existing methods often present limitations. For example, one approach utilizing MeI at 145 °C or 1,2-dichlorobenzene at 200 °C proposes a carbene intermediate leading to dimerization (Scheme 1a).^20,21^

Another method involves the reaction of these compounds with terminal alkynes to obtain the corresponding 3-formyl-2*H*-indazoles. While efficient, this method requires a large excess of copper species, high dilutions, and extended reaction times.^22−24^ Alternatively, 2*H*-indazoles can be formed under harsh conditions with low yields (Scheme 1b).^25^ Another example is the synthesis of alkyne-linked bis-2*H*-indazoles, prepared by the double cyclization of ethyne -or butadiyne-linked phenyltriazene.^26^ Two azo-ene-butadiyne conjugated systems undergo CuCl-mediated cyclization to afford isoindazolyl carbenoids that can be trapped with 2,3-dimethyl-2-butene as [2 + 1] cycloaddition products (Scheme 1c).^27^ Using either Cu or Rh complexes to induce cyclizations, it is possible to trap the isoindazole carbene with molecular oxygen to give α-ketoesters or water to afford α-hydroxyesters (Scheme 1d).^28,29^ Recently, a Pd/Cu cooperative catalysis approach for the synthesis of (3-isoindazolyl)allenes via cross-coupling of 2-alkynylazobenzenes and terminal alkynes has been described (Scheme 1e).^30^

Due to the significance of 2*H*-indazole scaffolds and the limited methods available for the synthesis of 3-alkenyl-2*H*-indazoles,^31−33^ this work aims to establish a general copper-catalyzed protocol for the intramolecular synthesis of these compounds (Scheme 1f).

This new approach seeks to overcome the drawbacks of previous methods by employing catalytic quantities of a copper catalyst and shorter reaction times and achieving the synthesis of a new family of 2*H*-indazoles through a hydroamination of the alkynyl fragment. This novel approach utilizes 2-alkynylazobenzenes by an intramolecular reaction, unlike previous methods that use triazenes to obtain indazole derivatives. The following sections detail the development and evaluation of this novel synthetic strategy, leading to the production of previously undescribed 3-alkenyl-2*H*-indazoles.

## Results and Discussion

We began our investigation using (*E*)-1-(2-(hex-1-yn-1-yl)phenyl)-2-phenyldiazene **1a** as our benchmark substrate.^34^ Initially, the reaction was conducted in acetonitrile at room temperature, with Cu(MeCN)_4_PF_6_ as the catalyst. This resulted in a moderate yield of 32% for desired product (*E*)-3-(pent-1-en-1-yl)-2-phenyl-2*H*-indazole **2a** (Table 1, entry 1). Increasing the temperature to 80 °C yielded only a minor improvement (Table 1, entry 2). Next, we explored different solvents with higher boiling points, such as dioxane, toluene, *m*-xylene, DMF, mesitylene, and trifluorotoluene (Table 1, entries 3–8). These solvents provided a significant improvement, affording the product in 75% yield with toluene at 110 °C. Following solvent optimization, we evaluated different copper catalysts. Cu(MeCN)_4_BF_4_ and Cu(dap)_2_Cl delivered disappointing yields for **2a** (Table 1, entries 9–10). Switching to CuBr or CuCl resulted in a slight increase, reaching 41% with CuBr (Table 1, entries 11–12). Unfortunately, Cu(OAc)_2_ did not offer any improvement (Table 1, entry 13). Finally, using Cu_2_O as the catalyst proved to be the most successful, achieving a remarkable yield of 81% (Table 1, entry 14).

**Table 1:** Optimization of Reaction Conditions

entry^a^	solvent	temp (°C)	catalyst	**2a** (%)^b^
1	MeCN	25	Cu(MeCN)_4_PF_6_	32
2	MeCN	80	Cu(MeCN)_4_PF_6_	40
3	Dioxane	100	Cu(MeCN)_4_PF_6_	69
4	Toluene	110	Cu(MeCN)_4_PF_6_	75
5	*m*-Xylene	140	Cu(MeCN)_4_PF_6_	74
6	DMF	150	Cu(MeCN)_4_PF_6_	64
7	Mesitylene	110	Cu(MeCN)_4_PF_6_	73
8	Trifluorotoluene	102	Cu(MeCN)_4_PF_6_	54
9	Toluene	110	Cu(MeCN)_4_BF_4_	13
10	Toluene	110	[Cu(dap)_2_Cl]	22
11	Toluene	110	CuBr	41
12	Toluene	110	CuCl	29
13	Toluene	110	Cu(OAc)_2_	34
**14**	**Toluene**	**110**	**Cu**_**2**_**O**	**81**

a Reaction conditions: 0.2 mmol of **1a** was stirred in solvent (0.1 M) under Ar at 110 °C for 3 h.

b Isolated yields by column chromatography.

**Scheme 2:**
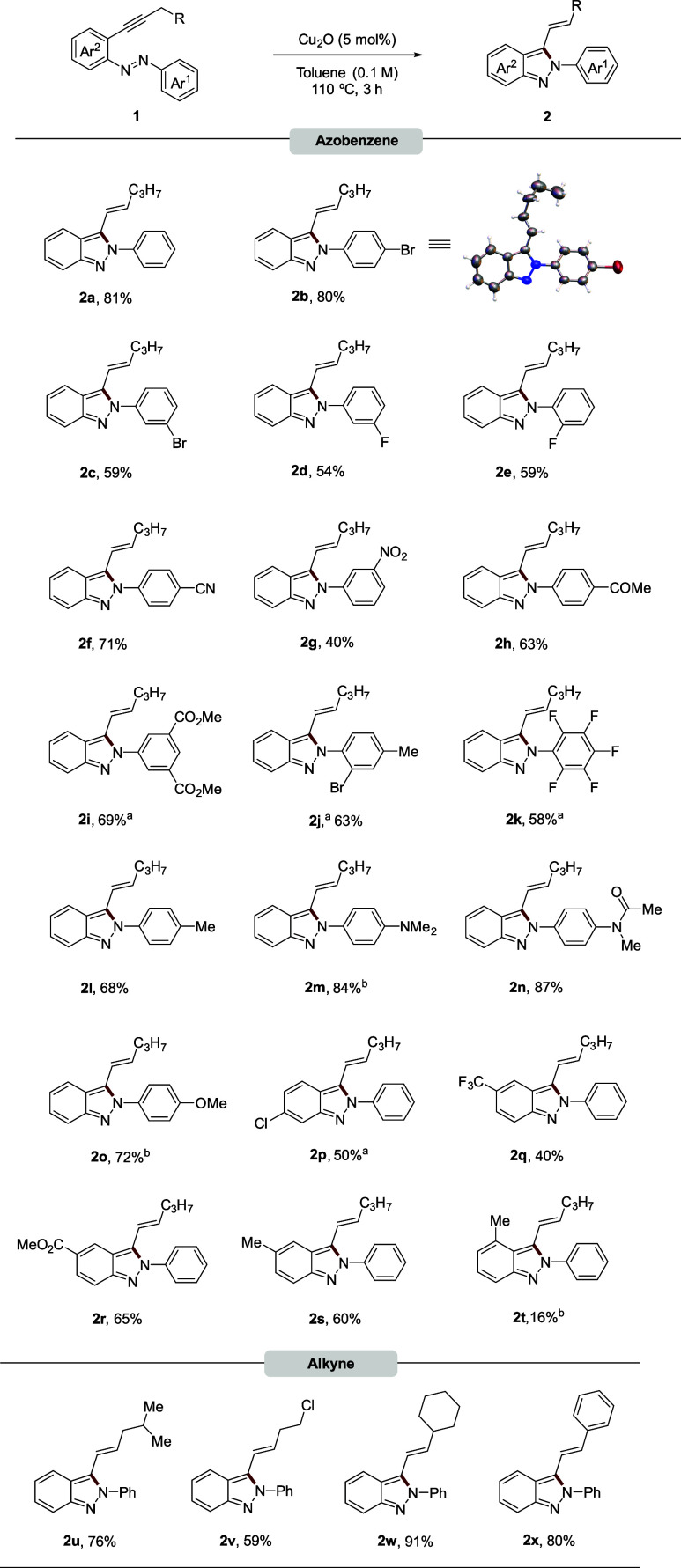
Substrate Scope in Azobenzene and Alkyne Moiety 6 h. 24 h.

We then investigated the reaction’s versatility under the optimized conditions (Table 1, Entry 14). A range of 2-alkynylazobenzenes, including those with halogens in *ortho*, *meta*, or *para* positions on Ar^1^, electron-withdrawing groups as cyano, nitro, acetyl, and methoxycarbonyl and even two substituents on the Ar^1^ ring, all successfully yielded the corresponding 3-alkenyl-2*H*-indazoles (**2b**–**2h**, **2i**–**2j**) in good yields (Scheme 2). The structure of **2b** was confirmed by X-ray diffraction.^35^ Fluorinated compounds, particularly relevant in medicinal chemistry due to their ability to influence the properties of drugs,^36^ were also compatible. Standard conditions provided 2*H*-indazoles **2k** and **2q** in 58% and 40% yields, respectively. This demonstrated the reaction’s tolerance for electron-donating groups present in methyl (**2l**), dimethylamine (**2m**), and methoxy (**2o**) derivatives. Amides (**1n**) were also successfully converted to 2*H*-indazoles (**2n**). Attempts to replace Ar^1^ with an alkyl group were unsuccessful (see SI for details). Modifications on the Ar^2^ ring, introducing electron-withdrawing or electron-donating groups at various positions, yielded excellent results for products **2p**-**2t**. Further exploration of structural diversity focused on modifications of the alkynyl moiety. Substrates with branched chains, cycloalkyl groups (cyclohexyl), halogenated chains, and even an aromatic ring on the alkyne all afforded the corresponding products (**2u**-**2x**) in very good yields (up to 91%) (Scheme 2).

**Scheme 3:**
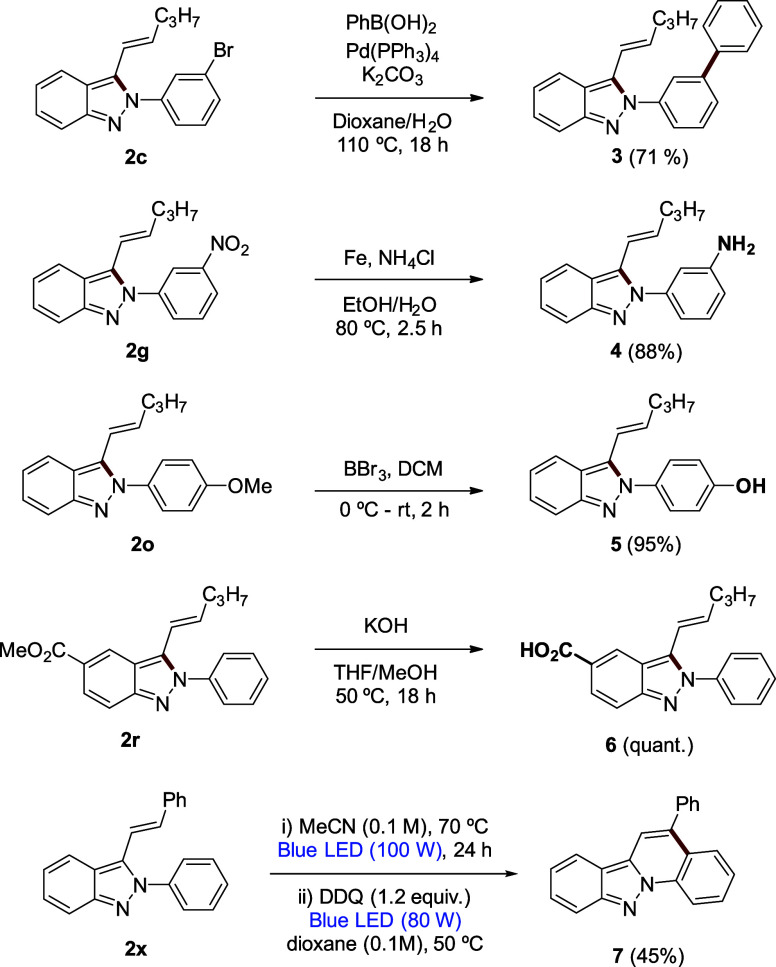
Derivatization of 3-Alkenyl-2*H*-Indazoles **2**

In order to enhance the applications of indazoles, we investigated methods to modify the 2*H*-indazole core structures represented in Scheme 3. A biphenyl moiety can be introduced onto indazole **2c** through Suzuki coupling to afford compound **3**. Reduction of the nitro group in **2g** efficiently yields the corresponding amino indazole **4**. Deprotection of the phenolic group on 3-alkenyl-2*H*-indazole **2o** cleanly provides compound **5**. Quantitative hydrolysis of the ester group in **2r** transforms it into compound **6**. Irradiating **2x** with blue LED light followed by treatment with DDQ under blue LED irradiation led to tetracycle **7** with a moderate yield.

**Scheme 4:**
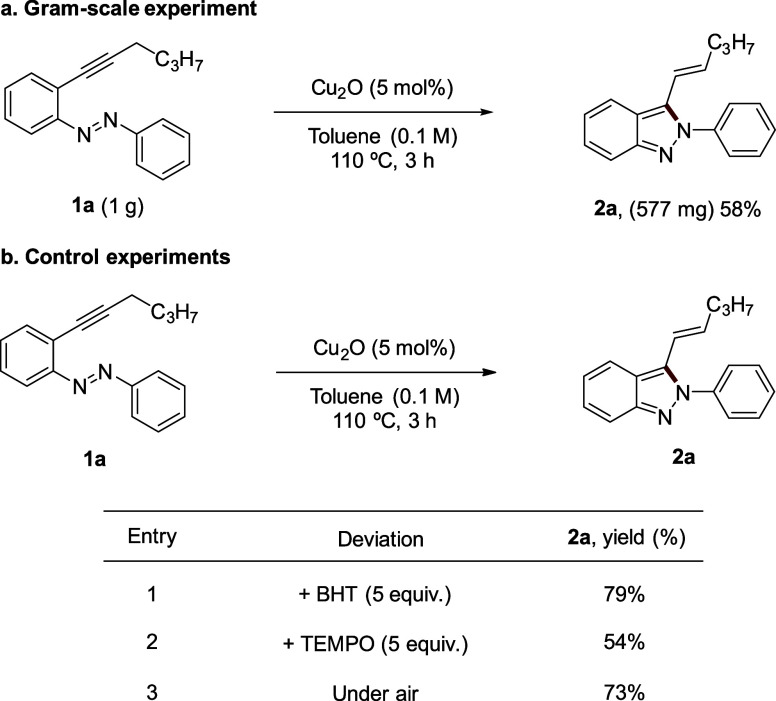
Additional Experiments

A gram-scale experiment was conducted using 2-alkynylazobenzene **1a**, resulting in the formation of the corresponding 2*H*-indazole **2a** with a 58% yield (Scheme 4a). To gain further insight into the reaction mechanism, we performed a series of control experiments. First, we introduced two different radical scavengers, BHT and TEMPO, which afforded the desired product **2a** in yields of 79 and 54%, respectively. These results suggest that the reaction likely proceeds through an ionic mechanism. Additionally, when the reaction was carried out under aerobic conditions, the yield of **2a** was similar to that obtained under an argon atmosphere (Scheme 4b).

**Scheme 5:**
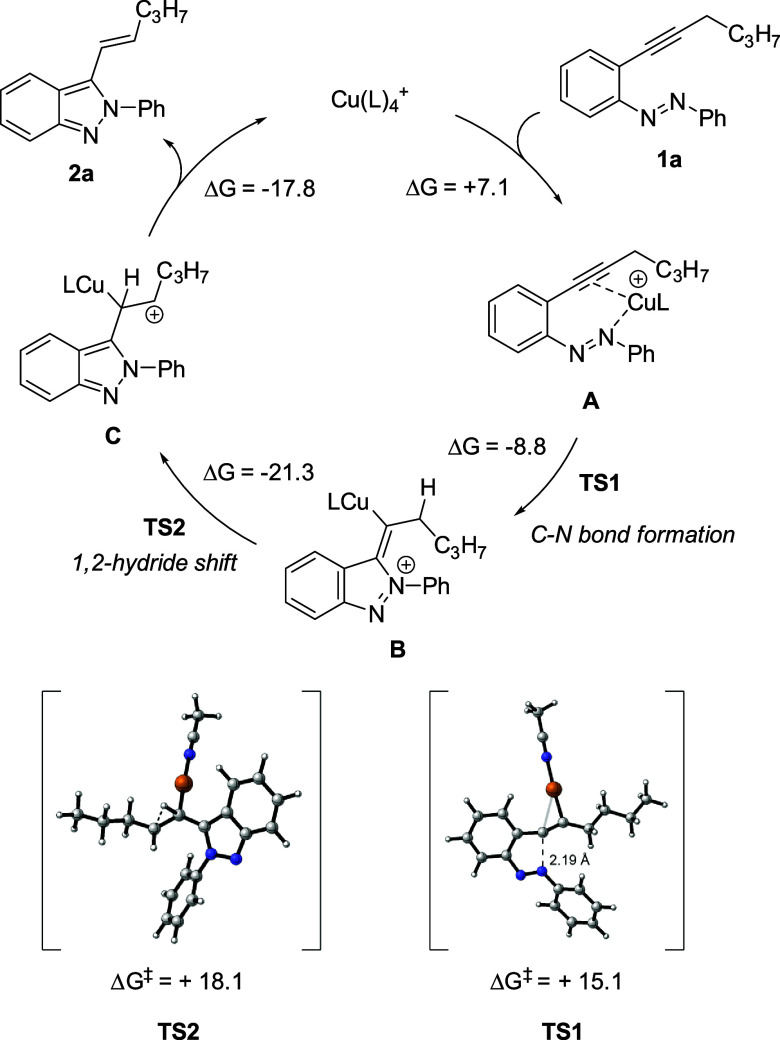
Plausible Mechanism for the Formation of 2. L = MeCN Intermediates and transition states computed (opt/freq) at M06/6-31G(d,p) level (cpcm, solvent = Toluene), temperature = 383.15 K. Energies are given in kcal/mol.

A plausible mechanistic hypothesis has been proposed to rationalize the copper-catalyzed hydroamination reaction that transforms 2-alkynylazobenzenes **1** into 2-alkenyl-2*H*-indazoles **2**. This hypothesis suggests an initial coordination of the copper catalyst to the alkynyl moiety, followed by the formation of a C–N bond and a C–H bond via a 1,2-hydride shift, ultimately leading to the formation of the final product. To explore this mechanistic hypothesis, density functional calculations were conducted (Scheme 5).

Initially, the copper complex coordinates simultaneously to the alkynyl moiety and a nitrogen atom of the azo group in a slightly endothermic process (+7.1 kcal/mol), forming intermediate **A**. Then, C–N bond formation occurs via a transition state with an energy of +15.1 kcal/mol, leading to intermediate **B**. The rate-determining step involves a 1,2-hydride shift with an energy value of +18.1 kcal/mol. Finally, intermediate **C** evolves into product **2**, regenerating the catalyst and closing the catalytic cycle.

## Conclusions

We successfully synthesized a novel family of 3-alkenyl-2*H*-indazoles. This synthesis was accomplished via an intramolecular copper-catalyzed cyclization of 2-alkynylazobenzenes. In contrast to the previously reported triazenes cyclization method, our approach offers several advantages: it requires only catalytic amounts of copper and exhibits shorter reaction times. Moreover, the resulting 3-alkenyl-2*H*-indazoles can be modified to broaden their range of functionalities. DFT calculations suggest that copper coordinates with both the alkyne and one nitrogen atom of the azo group, facilitating the cyclization process with the 1,2-hydride shift identified as the rate-determining step.

## Data Availability

The data underlying this study are available in the published article and its Supporting Information.

## Supporting Information Available

Detailed experimental procedures, characterization data, DFT calculations, X-ray crystallographic data, and copies of NMR spectra (PDF)
