# Supplementary material for: Copper-Catalyzed Hydroamination of 2-Alkynylazobenzenes: Synthesis of 3-Alkenyl-2H-Indazoles
Source: J Org Chem. 2024 Nov 2;89(22):16883–8. doi: 10.1021/acs.joc.4c02144 (PMC11574842; doi:10.1021/acs.joc.4c02144)
Supplement: Supplementary file 1 — jo4c02144_si_001.pdf [file jo4c02144_si_001.pdf]

Supporting Information

Copper-Catalyzed Hydroamination of 2-Alkynylazobenzenes: Synthesis of 3-Alkenyl-2*H*-Indazoles

Clara Mañas,[‡,‖] Juan Herrero-Bourdieu,[‡] Estíbaliz Merino[‡,‖]*

[‡] Universidad de Alcalá, Departamento de Química Orgánica y Química Inorgánica, Instituto de Investigación Andrés M. del Río (IQAR), Facultad de Farmacia, Alcalá de Henares, 28805 Madrid, Spain.

[‖] Instituto Ramón y Cajal de Investigación Sanitaria (IRYCIS), Ctra. De Colmenar Viejo, Km. 9.100, 28034 Madrid, Spain.

E-mail: estibaliz.merino@uah.es

CONTENT

General information	S3
Optimization of reaction conditions	S4
Control experiments	S5
Experimental procedure and characterization of 2*H*-indazoles	S10
X-Ray crystallographic data for **2b**	S27
DFT Calculations	S28
NMR Spectra	S34
References	S96

General information

All manipulations of air and moisture sensitive species were performed under argon atmosphere unless otherwise stated. Dry solvents, where necessary, were dried by a MBRAUN MB-SPS-800 apparatus. Starting materials were acquired from commercial sources and used without further purification. (*E*)-1-(2-alkynylphenyl)-2-phenyldiazenes were synthesized according to previously reported procedures.[1]

Reactions were monitored by thin-layer chromatography (TLC) carried out on 0.25 mm E. Merck silica gel plates (60FS-254) using UV light for visualization. Silica gel grade 60 (230-400 mesh, Silicycle Inc.) was used for column chromatography. Melting points were measured in open capillary tubes on a Stuart Scientific SMP3 melting point apparatus and are uncorrected. ^{1}H, ^{13}C and ^{19}F NMR spectra were recorded on either a Varian Mercury VX-300, Varian Unity 300, Bruker Avance Neo 400 or Varian Unity 500 MHz spectrometers at room temperature. Chemical shifts are given in ppm (δ) downfield from TMS. Coupling constants (J) are in Hertz (Hz) and signals are described as follows: s, singlet; d, doublet; dd, doublet of doublets; dt, double of triplets; dq, double of quadruplets; ddd, doublet of doublet of doublets; t, triplet; td, triplet of doublets, q, quadruplet; quint, quintuplet; sext, sextet; sept, septet; m, multiplet. Absorption spectra were recorded in a UV-VIS FLS980 Spectrophotometer (Edinburgh Instruments) equipped with a detector (200-1000 nm) that is allowed for absorbance measurements. High-resolution analysis (HRMS) was performed on an Agilent 6545 Q-TOF. Crystals for X-ray diffraction were grown by vapor diffusion crystallization in a mixture hexane/ethyl acetate. X-ray measurements were made using a Bruker D8 VENTURE PhtotonIII area-detector diffractometer with Cu-K α radiation (α = 1.54 Å).

Optimization of reaction conditions

Table S1. Solvent evaluation.

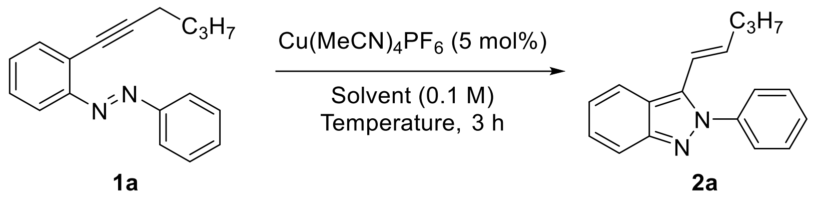

Entry	Solvent	Temperature (°C)	Yield (%)[a]
**1**	MeCN	25	32
**2**	MeCN	80	40
**3**	Dioxane	100	69
**4**	Toluene	110	75
**5**	*m*-Xylene	140	74
**6**	DMF	150	64
**7**	Mesitylene	110	73
**8**	Trifluorotoluene	102	54

[a] Isolated yields by column chromatography

Table S2. Catalyst evaluation.

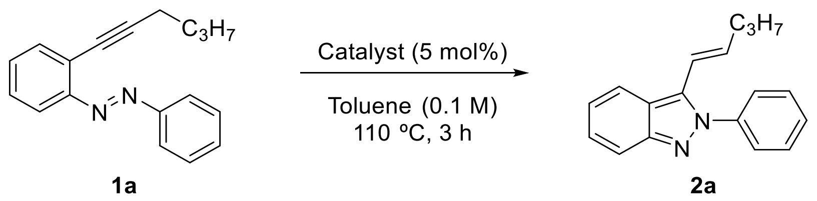

Entry	Solvent	Yield (%)[a]
**1**	$Cu(MeCN)_4PF_6$	75
**2**	$Cu(MeCN)_4BF_4$	13
**3**	$[Cu(dap)_2Cl]$	22
**4**	CuBr	41
**5**	CuCl	29
**6**	**Cu_2O**	**81**
**7**	$Cu(OAc)_2$	34

[a] Isolated yields by column chromatography

Control experiments

No conversion was observed in the reaction with copper (I) oxide at room temperature (entry 2). This outcome was probably due to the low solubility of Cu_2O in toluene at room temperature (< 14mg/L).[2]

Table S3. Control experiments for the synthesis of 2*H*-indazole **2a** with Cu_2O.

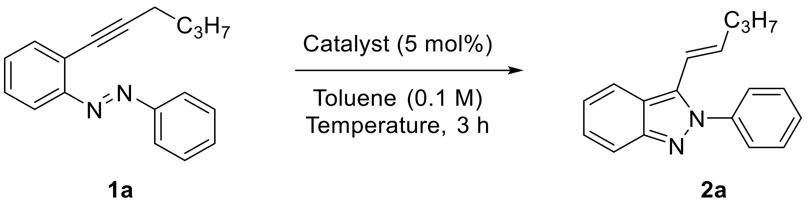

Entry	Catalyst	Temperature (°C)	Yield (%)
**1**	-	rt	0
**2**	Cu_2O	rt	0
**3**	-	110	14

The reaction was also performed under aerobic conditions. The general procedure was followed, using toluene as the solvent in an aerobic atmosphere. After 3 hours, the reaction mixture was cooled to room temperature and filtered through Celite. The solvent was then removed under reduced pressure. The crude product was purified by column chromatography using hexane/ethyl acetate (20:1) as the eluent, affording the corresponding 2*H*-indazole **2a** in a 73% yield.

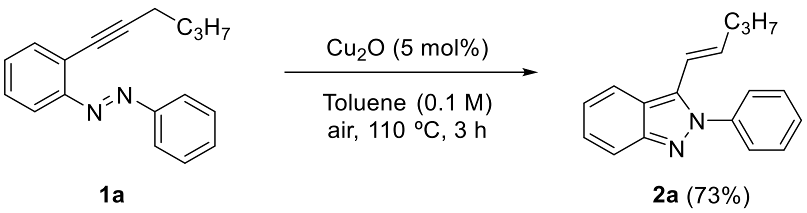

Scheme S1. Synthesis of 2*H*-indazole **2a** under aerobic conditions

Radical inhibitor experiment

The general procedure was followed with the addition of BHT (5 equiv.) or TEMPO (5 equiv.). After 3 hours, the reaction mixture was cooled to room temperature, filtered and analyzed by ^{1}H-NMR. Almost full conversion was achieved, with only trace amounts of the starting material **1a** detected. After purification by column chromatography, the desired (*E*)-3-(pent-1-enyl)-2-phenyl-2*H*-indazole (**2a**) was obtained with a 79% yield using BHT and a 54% yield with TEMPO.

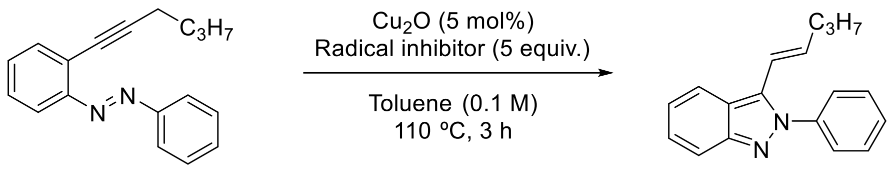

1a **2a**

BHT:	79%
TEMPO:	54%

Scheme S2. Radical inhibitor experiments.

Experiment with both isomers of 1a

An experiment was conducted using a mixture of the *E* and *Z* isomers of 2-alkynylazobenzene **1a**. A solution of **1a** (0.06 mmol) in toluene-d_8 (0.1 M, 0.6 mL) was prepared in a J-Young tube and irradiated with a 40 W blue LED for 15 minutes, as previously established,[1] to achieve a photostationary state. After this time, the *E:Z* isomer ratio was 69:31. At this point, Cu_2O (0.003 mmol, 5 mol%) was added, and the reaction mixture was heated to 110 °C. ^{1}H-NMR spectra were acquired over time. After 10 min, no *Z*-isomer was present, as the characteristic signal at 2.15 ppm had disappeared (Figure S1). This indicates that the reaction proceeds exclusively with the *E*-isomer, as no *Z*-isomer remains under the reaction conditions. After 30 minutes, the formation of 2*H*-indazole **2a** was observed (signal at 1.94 ppm), and after 3 hours, only **2a** was present in the crude mixture.

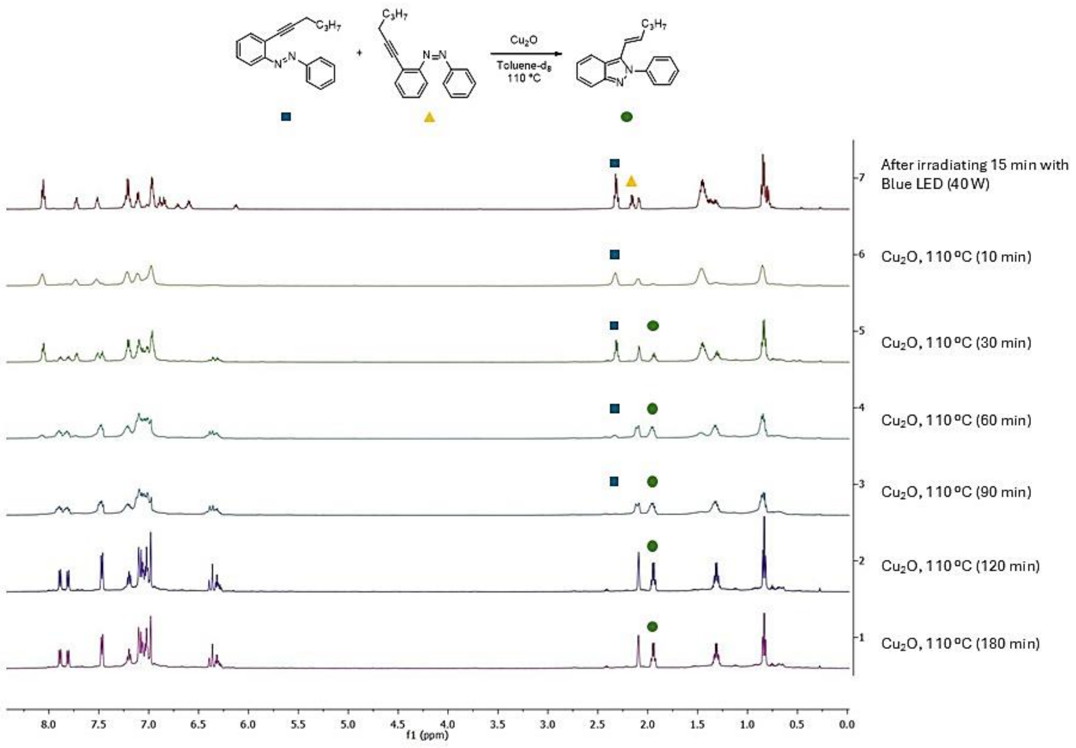

Figure S1. ^{1}H-spectra of the reaction of a mixture *E:Z* of **2a** over time.

Attempted substrates

The synthesis of the following substrates was attempted using either the Mills reaction or diazonium salt formation; however, in no case was the formation of the corresponding substrate detected:

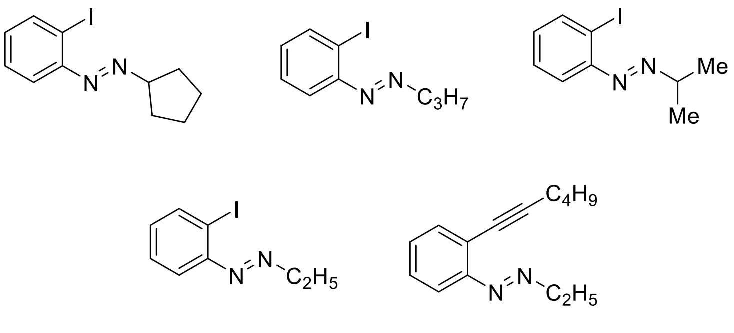

Figure S2. Attempts to replace Ar[1] with an alkyl group.

Visible light-promoted reaction setup

For the synthesis of 5-phenylindazolo[2,3-a]quinoline (**7**), the reaction was carried out using a custom-made photoreactor equipped with two 50 W Chip LED COB EPISTAR 35mil lights. The Schlenk tube was positioned centrally, approximately 2 cm from the LEDs. To maintain a temperature of 70 ºC, one fan was placed above the Schlenk tube and another on the side (**Figure S3**). The emission spectrum of the 50 W blue LED chip is shown in **Figure S4**.

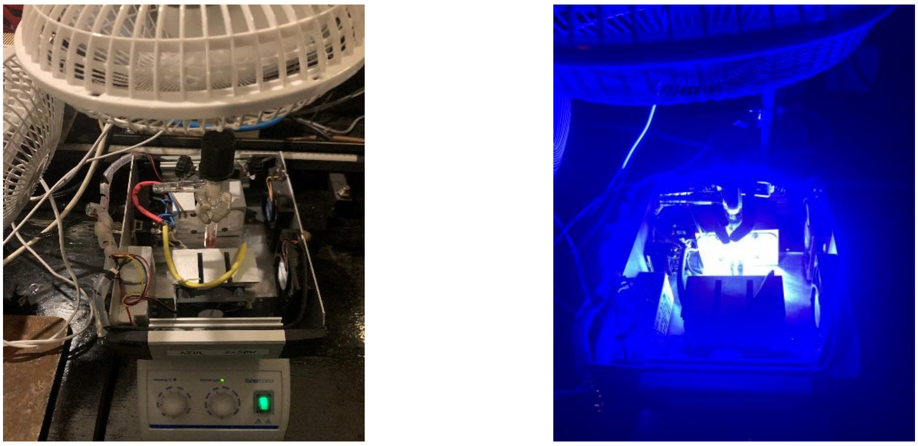

Figure S3. Photochemical experimental setup for the first step in the synthesis of 5-phenylindazolo[2,3-*a*]quinoline (**7**).

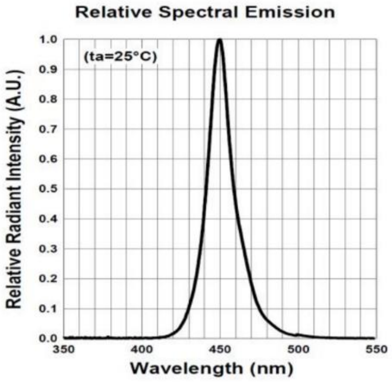

Figure S4. Emission spectra of the 50 W blue LED chips.

In the second reaction step, the reaction was exposed to two Kessil 40 W blue LED lamps (model: A160WE Tuna Blue), positioned approximately 3 cm from the Schlenk tube. To maintain a temperature of 50 ºC, one fan was placed above the Schlenk tube and another on the side (**Figure S5**). The emission spectra of the Kessil 40 W blue LED lamp is shown in **Figure S6**.

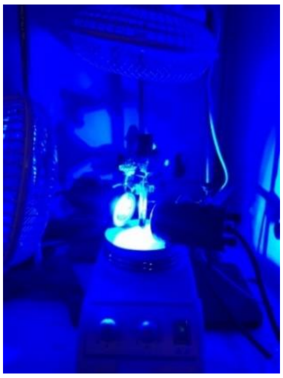

Figure S5. Photochemical experimental setup for the second step of the synthesis of 5-phenylindazolo[2,3-a]quinoline (**7**).

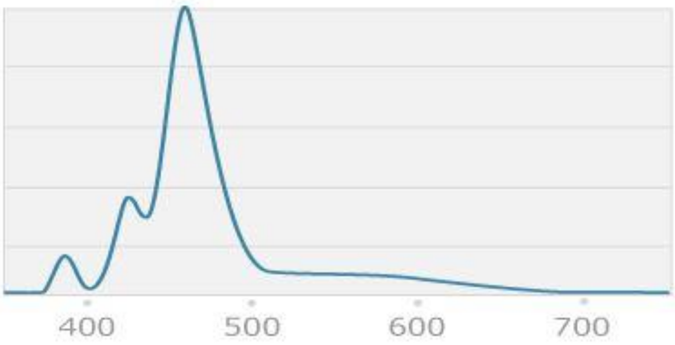

Figure S6. Emission spectrum of the Kessil blue LED lamp

Experimental procedure and characterization of 2*H*-indazoles

2-(Hex-1-yn-1-yl)aniline[1]

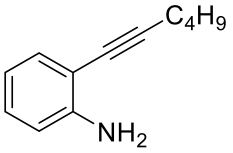

Colorless oil, 77% yield.

^{1}H-NMR (300 MHz, CDCl$_3$) δ (ppm) 7.29–7.20 (m, 1H), 7.14–7.02 (m, 1H), 6.73–6.61 (m, 2H), 4.16 (s, 2H), 2.48 (td, *J* = 7.0, 1.2 Hz, 2H), 1.67–1.43 (m, 4H), 0.96 (t, *J* = 7.2 Hz, 3H).

^{13}C-NMR (126 MHz, CDCl$_3$) δ (ppm) 147.7, 132.2, 128.9, 118.0, 114.3, 109.1, 95.9, 77.1, 31.2, 22.2, 19.5, 13.8.

General procedure for the synthesis of 2*H*-indazoles 2

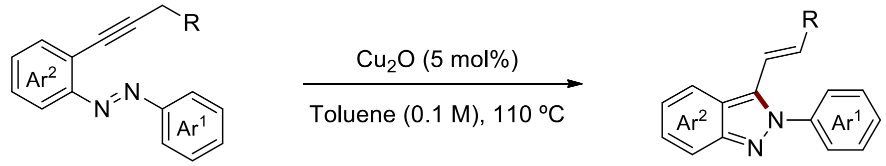

1 **2**

The corresponding alkynylazobenzene **1** (0.2 mmol, 1 equiv.) and Cu_2O (0.01 mmol, 5 mol%) were dissolved in toluene (0.1 M) in a Schlenk tube (5 mL). The reaction mixture was heated at 110 ^oC in a sand bath until completion. The crude mixture was cooled down to room temperature and filtered through Celite, afterwards the solvent was removed under reduced pressure. The crude was purified by column chromatography using mixtures of hexane and ethyl acetate as eluent to yield the corresponding 2*H*-indazoles **2**.

(*E*)-3-(Pent-1-enyl)-2-phenyl-2*H*-indazole (2a)

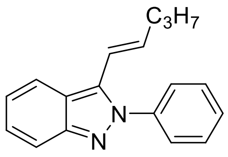

Eluent: Hexane/EtOAc (20:1). Yellow oil (40 mg, 81% yield).

^{1}H-NMR (300 MHz, $CDCl_3$) δ (ppm) 7.87 (dt, *J* = 8.4, 1.1 Hz, 1H), 7.75 (dt, *J* = 8.7, 0.9 Hz, 1H), 7.65 – 7.46 (m, 5H), 7.35 (ddd, *J* = 8.7 Hz, 6.6 Hz, 0.9 Hz, 1H), 7.15 (ddd, *J* = 1.0 Hz, 8.5 Hz, 6.6 Hz, 1H), 6.63 – 6.41 (m, 2H), 2.32 – 2.20 (m, 2H), 1.54 (sext, *J* = 7.3 Hz, 2H), 0.98 (t, *J* = 7.4 Hz, 3H).

^{13}C {^{1}H} NMR (75 MHz, $CDCl_3$) δ (ppm) 149.1, 140.1, 136.1, 133.7, 129.2, 128.8, 126.8, 126.4, 122.3, 121.0, 120.2, 118.5, 118.0, 35.8, 22.5, 13.8.

HRMS (ESI+) m/z calculated for $C_{18}H_{19}N_2$ [M+H]$^+$: 263.1543, found [M+H]$^+$: 263.1543.

(*E*)-2-(4-Bromophenyl)-3-(pent-1-enyl)-2*H*-indazole (2b)

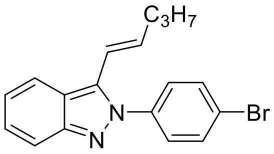

Eluent: Hexane/EtOAc (20:1). White solid (51 mg, 80% yield).

M.p. 40-41 ^{o}C.

^{1}H-NMR (300 MHz, $CDCl_3$) δ (ppm) 7.84 (dt, *J* = 8.6, 1.1 Hz, 1H), 7.73 (dt, *J* = 8.8, 1.0 Hz, 1H), 7.60 – 7.54 (m, 2H), 7.55 – 7.45 (m, 2H), 7.35 (ddd, *J* = 8.8, 6.6, 1.1 Hz, 1H), 7.15 (ddd, *J* = 8.5, 6.6, 1.0 Hz, 1H), 6.63 – 6.38 (m, 2H), 2.33 – 2.20 (m, 2H), 1.55 (sext, *J* = 7.3 Hz, 2H), 0.98 (t, *J* = 7.4 Hz, 3H).

^{13}C {^{1}H} NMR (126 MHz, $CDCl_3$) δ (ppm) 149.3, 139.2, 136.7, 133.8, 132.5, 127.8, 127.1, 122.6, 122.5, 121.0, 120.4, 118.2, 118.0, 35.9, 22.5, 13.8.

HRMS (ESI+) m/z calculated for $C_{18}H_{18}BrN_2$ [M+H]$^+$: 343.0629, found [M+H]$^+$: 343.0627.

(*E*)-2-(3-Bromophenyl)-3-(pent-1-enyl)-2*H*-indazole (2c)

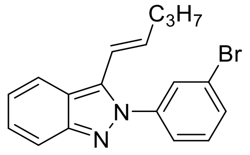

Eluent: Hexane/EtOAc (20:1). White solid (37 mg, 59% yield).

M.p. 49-50 ^{o}C.

^{1}H-NMR (300 MHz, CDCl$_3$) δ (ppm) 7.89 – 7.78 (m, 2H), 7.73 (dt, *J* = 8.8, 0.9 Hz, 1H), 7.62 (ddd, *J* = 8.0, 1.9, 1.1 Hz, 1H), 7.55 (ddd, *J* = 8.0, 2.1, 1.1 Hz, 1H), 7.42 (d, *J* = 8.0 Hz, 1H), 7.59 – 7.51 (m, 1H), 7.15 (ddd, *J* = 8.5, 6.6, 0.9 Hz, 1H), 6.61 – 6.39 (m, 2H), 2.28 (q, *J* = 7.1 Hz, 2H), 1.60 – 1.52 (m, 2H), 0.99 (t, *J* = 7.3 Hz, 3H).

^{13}C {^{1}H} NMR (101 MHz, CDCl$_3$) δ (ppm) 149.3, 141.2, 137.0, 134.0, 131.9, 130.4, 129.5, 127.2, 124.9, 122.8, 122.6, 121.0, 120.4, 118.1, 118.0, 35.9, 22.4, 13.8.

HRMS (ESI+) m/z calculated for $C_{18}H_{18}BrN_2$ [M+H]$^+$: 341.0648, found [M+H]$^+$: 341.0652.

(*E*)-2-(3-Fluorophenyl)-3-(pent-1-enyl)-2*H*-indazole (2d)

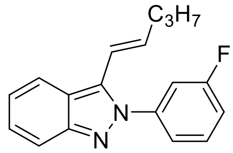

Eluent: Hexane/EtOAc (20:1). Yellow oil (29 mg, 54% yield).

^{1}H-NMR (300 MHz, CDCl$_3$) δ (ppm) 7.84 (dt, *J* = 8.5, 1.1 Hz, 1H), 7.73 (dt, *J* = 8.8, 1.0 Hz, 1H), 7.57 – 7.28 (m, 4H), 7.23 – 7.10 (m, 2H), 6.66 – 6.39 (m, 2H), 2.35 – 2.21 (m, 2H), 1.57 (sext, *J* = 7.3 Hz, 2H), 0.99 (t, *J* = 7.3 Hz, 3H).

^{19}F NMR (376 MHz, CDCl$_3$) δ (ppm) δ -110.85 – -110.94 (m).

^{13}C {^{1}H} NMR (101 MHz, CDCl$_3$) δ (ppm) 162.8 (d, *J* = 248.4 Hz), 149.2, 141.5 (d, *J* = 9.9 Hz), 136.9, 133.9, 130.5 (d, *J* = 8.9 Hz), 127.2, 122.6, 122.0 (d, *J* = 3.3 Hz), 121.0, 120.4, 118.2, 118.0, 115.8 (d, *J* = 21.1 Hz), 114.0 (d, *J* = 24.4 Hz), 35.9, 22.5, 13.8.

HRMS (ESI+) m/z calculated for $C_{18}H_{18}FN_2$ [M+H]$^+$: 281.1449, found [M+H]$^+$: 281.1454.

(*E*)-2-(2-Fluorophenyl)-3-(pent-1-enyl)-2*H*-indazole (2e)

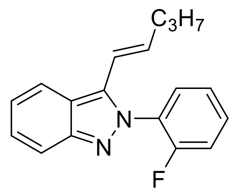

Eluent: Hexane/EtOAc (20:1). Yellow oil (34 mg, 59% yield).

^{1}H-NMR (300 MHz, CDCl$_3$) δ (ppm) 7.88 (dt, *J* = 8.6, 1.1 Hz, 1H), 7.74 (dt, *J* = 8.7, 1.0 Hz, 1H), 7.61 – 7.46 (m, 2H), 7.40 – 7.26 (m, 3H), 7.15 (ddd, *J* = 8.5, 6.6, 0.9 Hz, 1H), 6.53 (dt, *J* = 16.1, 7.0 Hz, 1H), 6.31 (dq, *J* = 16.1, 1.6 Hz, 1H), 2.22 (qd, *J* = 7.1, 1.4 Hz, 2H), 1.50 (sext, *J* = 7.3 Hz, 2H), 0.94 (t, *J* = 7.4 Hz, 3H).

^{19}F NMR (376 MHz, CDCl$_3$) δ (ppm) -118.19 – -118.41 (m).

^{13}C {^{1}H} NMR (101 MHz, CDCl$_3$) δ (ppm) 156.8 (d, *J* = 253.7 Hz), 149.6, 136.5, 135.4, 131.1 (d, *J* = 7.7 Hz), 129.6, 128.1, 128.0 (d, *J* = 12.2 Hz), 124.7 (d, *J* = 4.0 Hz), 122.4, 121.0, 119.6, 118.1, 117.5 (d, *J* = 1.8 Hz), 116.8 (d, *J* = 19.6 Hz), 35.9, 22.5, 13.7.

HRMS (ESI+) m/z calculated for $C_{18}H_{18}FN_2$ [M+H]$^+$: 281.1449, found [M+H]$^+$: 281.1452.

(*E*)-4-(3-(Pent-1-enyl)-2*H*-indazol-2-yl)benzonitrile (2f)

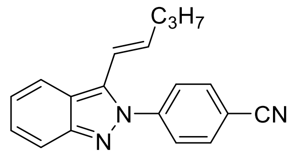

Eluent: Hexane/EtOAc (5:1). Yellow solid (39 mg, 71% yield)

M.p. 101-102 ^oC.

^{1}H-NMR (300 MHz, CDCl$_3$) δ (ppm) 7.92 – 7.75 (m, 5H), 7.72 (dt, *J* = 8.8, 1.0 Hz, 1H), 7.36 (ddd, *J* = 8.8, 6.6, 1.1 Hz, 1H), 7.16 (ddd, *J* = 8.6, 6.6, 0.9 Hz, 1H), 6.68 – 6.37 (m, 2H), 2.36 – 2.23 (m, 2H), 1.62 – 1.53 (m, 2H), 1.00 (t, *J* = 7.4 Hz, 3H).

^{13}C {^{1}H} NMR (101 MHz, CDCl$_3$) δ (ppm) 149.7, 143.7, 137.8, 134.1, 133.3, 127.7, 126.7, 123.0, 121.0, 120.9, 118.2, 118.1, 117.9, 112.3, 35.9, 22.4, 13.8.

HRMS (ESI+) m/z calculated for $C_{19}H_{18}N_3$ [M+H]$^+$: 288.1495, found [M+H]$^+$: 288.1501.

(*E*)-2-(3-Nitrophenyl)-3-(pent-1-en-1-yl)-2*H*-indazole (2g)

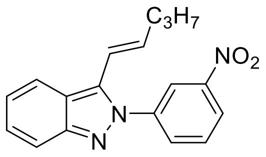

Eluent: Hexane/EtOAc (5:1). Yellow solid (24 mg, 40% yield).

M.p. 74-75°C.

^{1}H-NMR (300 MHz, CDCl$_3$) δ (ppm) 8.54 (t, *J* = 2.2 Hz, 1H), 8.34 (ddd, *J* = 8.2, 2.3, 1.1 Hz, 1H), 8.03 (ddd, *J* = 8.8, 1.9, 0.9 Hz, 1H), 7.84 (dt, *J* = 8.6, 1.1 Hz, 1H), 7.78 – 7.70 (m, 2H), 7.37 (ddd, *J* = 8.8, 6.6, 1.1 Hz, 1H), 7.17 (ddd, *J* = 8.6, 6.6, 1.0 Hz, 1H), 6.66 – 6.38 (m, 2H), 2.30 (q, *J* = 7.0 Hz, 2H), 1.62 – 1.51 (m, 2H), 1.00 (t, *J* = 7.4 Hz, 3H).

^{13}C {^{1}H} NMR (126 MHz, CDCl$_3$) δ (ppm) 149.6, 148.6, 141.2, 138.1, 134.2, 131.8, 130.3, 127.7, 123.3, 123.0, 121.2, 121.0, 120.7, 118.1, 117.7, 35.9, 22.4, 13.8.

HRMS (ESI+) m/z calculated for $C_{18}H_{18}N_3O_2$ [M+H]$^+$: 308.1394 , found [M+H]$^+$: 308.1396.

(*E*)-1-(4-(3-(Pent-1-enyl)-2*H*-indazol-2-yl)phenyl)ethan-1-one (2h)

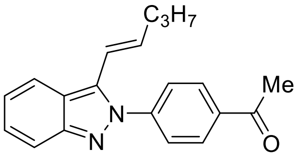

Eluent: Hexane/EtOAc (20:1). Yellow oil (34 mg, 63% yield).

^{1}H-NMR (300 MHz, CDCl$_3$) δ (ppm) 8.18 – 8.09 (m, 2H), 7.84 (dd, *J* = 8.5, 1.0 Hz, 1H), 7.80 – 7.68 (m, 3H), 7.35 (ddd, *J* = 8.8, 6.6, 1.1 Hz, 1H), 7.15 (ddd, *J* = 8.6, 6.6, 0.9 Hz, 1H), 6.65 – 6.41 (m, 2H), 2.68 (s, 3H), 2.28 (dt, *J* = 8.1, 6.6 Hz, 2H), 1.55 (sext, *J* = 7.3 Hz, 2H), 0.99 (t, *J* = 7.4 Hz, 3H).

^{13}C {^{1}H} NMR (101 MHz, CDCl$_3$) δ (ppm) 197.2, 149.5, 143.8, 137.1, 136.8, 134.0, 129.4, 127.4, 126.2, 122.7, 121.1, 120.7, 118.2, 118.1, 35.9, 26.9, 22.5, 13.8.

HRMS (ESI+) m/z calculated for $C_{20}H_{21}N_2O$ [M+H]$^+$: 305.1648, found [M+H]$^+$: 305.1652.

(*E*)-Dimethyl 5-(3-(pent-1-en-1-yl)-2*H*-indazol-2-yl)isophthalate (2i)

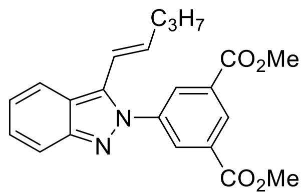

Eluent: Hexane/EtOAc (10:1). Yellow oil (40 mg, 69% yield).

^{1}H NMR (500 MHz, $CDCl_3$) δ (ppm) 8.79 (t, *J* = 1.5 Hz, 1H), 8.51 – 8.49 (m, 2H), 7.84 (dt, *J* = 8.5, 1.1 Hz, 1H), 7.73 (dt, *J* = 8.7, 1.0 Hz, 1H), 7.36 (ddd, *J* = 8.8, 6.6, 1.1 Hz, 1H), 7.16 (ddd, *J* = 8.6, 6.6, 1.0 Hz, 1H), 6.61 – 6.52 (m, 1H), 6.44 (dt, *J* = 15.9, 1.4 Hz, 1H), 3.98 (s, 6H), 2.30 – 2.26 (m, 2H), 1.55 (sext, *J* = 7.3 Hz, 2H), 0.99 (t, *J* = 7.4 Hz, 3H).

^{13}C {^{1}H} NMR (126 MHz, $CDCl_3$) δ (ppm) 165.3, 149.5, 140.7, 137.7, 134.1, 132.0, 131.1, 130.6, 127.4, 122.7, 121.0, 120.6, 118.0, 117.9, 52.8, 35.8, 22.4, 13.8.

HRMS (ESI+) m/z calculated for $C_{22}H_{23}N_2O_4$ $[M+H]^+$: 379.1652, found $[M+H]^+$: 379.1657.

(*E*)-2-(2-Bromo-4-methylphenyl)-3-(pent-1-enyl)-2*H*-indazole (2j)

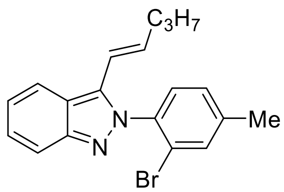

Eluent: Hexane/EtOAc (20:1). Yellow oil (37 mg, 63% yield).

^{1}H NMR (300 MHz, $CDCl_3$) δ (ppm) 7.89 (dt, *J* = 8.6, 1.1 Hz, 1H), 7.75 (dt, *J* = 8.8, 1.0 Hz, 1H), 7.64 – 7.51 (m, 1H), 7.41 – 7.29 (m, 2H), 7.32 – 7.23 (m, 1H), 7.15 (ddd, *J* = 8.6, 6.6, 1.0 Hz, 1H), 6.54 – 6.40 (m, 1H), 6.19 (dt, *J* = 16.1, 1.5 Hz, 1H), 2.46 (s, 3H), 2.25 – 2.12 (m, 2H), 1.48 (sext, *J* = 7.3 Hz, 2H), 0.93 (t, *J* = 7.4 Hz, 3H).

^{13}C {^{1}H} NMR (126 MHz, $CDCl_3$) δ (ppm) 149.2, 141.8, 136.9, 136.0, 133.9, 129.4, 129.0, 126.9, 122.3, 121.5, 121.1, 119.3, 118.2, 117.8, 110.2, 35.9, 22.6, 21.2, 13.8.

HRMS (ESI+) m/z calculated for $C_{19}H_{20}BrN_2$ $[M+H]^+$: 355.0804, found $[M+H]^+$: 355.0810.

(*E*)-3-(Pent-1-en-1-yl)-2-(perfluorophenyl)-2*H*-indazole (2k)

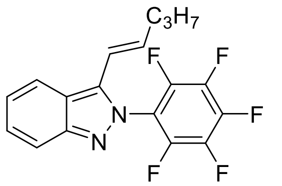

Eluent: Hexane/EtOAc (20:1). Yellow oil (23 mg, 58% yield).

^{1}H MR (300 MHz, CDCl$_3$) δ (ppm) 7.90 – 7.79 (m, 1H), 7.71 (dt, *J* = 8.8, 1.0 Hz, 1H), 7.38 (ddd, *J* = 8.8, 6.6, 1.1 Hz, 1H), 7.18 (ddd, *J* = 8.6, 6.6, 1.0 Hz, 1H), 6.64 – 6.48 (m, 1H), 6.24 (d, *J* = 15.9 Hz, 1H), 2.25 (qd, *J* = 7.2, 1.5 Hz, 2H), 1.50 (d, *J* = 7.3 Hz, 2H), 0.96 (t, *J* = 7.4 Hz, 3H).

^{19}F NMR (282 MHz, CDCl$_3$) δ (ppm) -144.16 – -144.67 (m, 2F), -150.44 (tt, *J* = 21.1, 1.9 Hz, 1F), -160.20 – -160.44 (m, 2F).

^{13}C {^{1}H} NMR (126 MHz, CDCl$_3$) δ (ppm) 150.6, 138.5, 136.7, 127.9, 123.2, 121.0, 119.4, 118.3, 116.1, 35.9, 22.4, 13.7. *C coupled with fluorine atoms are not observed.

HRMS (ESI+) m/z calculated for $C_{18}H_{14}F_5N_2$ [M+H]$^+$: 353.1072, found [M+H]$^+$: 353.1073.

(*E*)-3-(Pent-1-enyl)-2-(*p*-tolyl)-2*H*-indazole (2l)

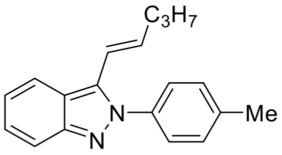

Eluent: Hexane/EtOAc (20:1). Yellow oil (36 mg, 68% yield).

^{1}H NMR (300 MHz, CDCl$_3$) δ (ppm) 7.86 (dt, *J* = 8.5, 1.1 Hz, 1H), 7.74 (dt, *J* = 8.7, 1.0 Hz, 1H), 7.50 – 7.43 (m, 2H), 7.36 – 7.29 (m, 3H), 7.14 (ddd, *J* = 8.5, 6.6, 1.0 Hz, 1H), 6.63 – 6.42 (m, 2H), 2.46 (s, 3H), 2.28 – 2.20 (m, 2H), 1.54 (sext, *J* = 7.4 Hz, 2H), 0.98 (t, *J* = 7.3 Hz, 3H).

^{13}C {^{1}H} NMR (101 MHz, CDCl$_3$) δ (ppm) 149.0, 138.8, 137.7, 135.8, 133.7, 129.8, 126.7, 126.1, 122.2, 121.0, 120.2, 118.7, 118.0, 35.9, 22.5, 21.4, 13.8.

HRMS (ESI+) m/z calculated for $C_{19}H_{21}N_2$ [M+H]$^+$: 277.1699, found [M+H]$^+$: 277.1701.

(*E*)-*N*,*N*-Dimethyl-4-(3-(pent-1-enyl)-2*H*-indazol-2-yl)aniline (2m)

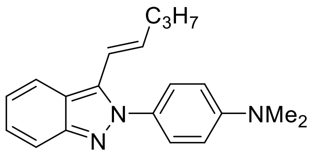

Eluent: Hexane/EtOAc (5:1). Yellow solid (49 mg, 84% yield). **M.p.** 149-150 ^{o}C.

^{1}H NMR (300 MHz, $CDCl_3$) δ (ppm) 7.88 – 7.84 (m, 1H), 7.76 – 7.71 (m, 1H), 7.47 – 7.37 (m, 2H), 7.35 – 7.26 (m, 1H), 7.15 – 7.09 (m, 1H), 6.79 (dd, *J* = 9.1, 0.9 Hz, 2H), 6.56 – 6.41 (m, 2H), 3.04 (d, *J* = 1.0 Hz, 6H), 2.31 – 2.18 (m, 2H), 1.63 – 1.44 (m, 2H), 0.98 (t, *J* = 7.4 Hz, 3H).

^{13}C {^{1}H} NMR (101 MHz, $CDCl_3$) δ (ppm) 150.6, 148.8, 135.1, 133.5, 129.2, 127.1, 126.4, 121.8, 120.9, 120.0, 119.0, 117.9, 112.1, 40.6, 35.9, 22.6, 13.8.

HRMS (ESI+) m/z calculated for $C_{20}H_{24}N_3$ $[M+H]^+$: 306.1965, found $[M+H]^+$: 306.1966.

(*E*)-*N*-Methyl-*N*-(4-(3-(pent-1-enyl)-2*H*-indazol-2-yl)phenyl)acetamide (2n)

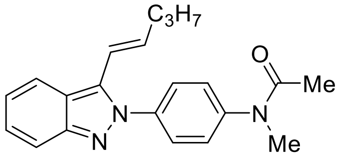

Eluent: Hexane/EtOAc (2:1). Yellow solid (59 mg, 87% yield).

M.p. 105-106 ^{o}C.

^{1}H NMR (300 MHz, $CDCl_3$) δ (ppm) 7.86 (dt, *J* = 8.5, 1.1 Hz, 1H), 7.73 (dd, *J* = 8.7, 1.0 Hz, 1H), 7.68 – 7.63 (m, 2H), 7.42 – 7.32 (m, 3H), 7.16 (ddd, *J* = 7.7, 6.7, 1.0 Hz, 1H), 6.63 – 6.45 (m, 2H), 3.34 (s, 3H), 2.33 – 2.24 (m, 2H), 1.97 (s, 3H), 1.55 (sext, *J* = 7.3 Hz, 2H), 0.99 (t, *J* = 7.4 Hz, 3H).

^{13}C {^{1}H} NMR (101 MHz, $CDCl_3$) δ (ppm) 170.5, 149.3, 144.8, 139.4, 136.9, 133.8, 128.0, 127.5, 127.2, 122.6, 121.0, 120.4, 118.2, 118.0, 37.4, 35.9, 22.7, 22.5, 13.8.

HRMS (ESI+) m/z calculated for $C_{21}H_{24}N_3O$ $[M+H]^+$: 334.1914, found $[M+H]^+$: 334.1918.

(*E*)-2-(4-Methoxyphenyl)-3-(pent-1-enyl)-2*H*-indazole (2o)

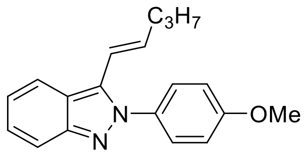

Eluent: Hexane/EtOAc (5:1). Yellow oil (35 mg, 72% yield).

^{1}H NMR (300 MHz, CDCl$_3$) δ (ppm) 7.86 (dt, *J* = 8.5, 1.2 Hz, 1H), 7.74 (dt, *J* = 8.8, 1.2 Hz, 1H), 7.55 – 7.45 (m, 2H), 7.39 – 7.28 (m, 1H), 7.19 – 7.08 (m, 1H), 7.08 – 6.99 (m, 2H), 6.61 – 6.36 (m, 2H), 3.89 (s, 3H), 2.25 (dt, *J* = 7.9, 6.4 Hz, 2H), 1.53 (sext, *J* = 7.3 Hz, 2H), 0.97 (t, *J* = 7.4 Hz, 3H).

^{13}C {^{1}H} NMR (75 MHz, CDCl$_3$) δ (ppm) 159.9, 148.9, 135.8, 133.8, 133.1, 127.6, 126.7, 122.2, 120.9, 120.1, 118.6, 117.9, 114.4, 55.7, 35.9, 22.5, 13.8.

HRMS (ESI+) m/z calculated for $C_{19}H_{21}N_2O$ [M+H]$^+$: 293.1648, found [M+H]$^+$: 293.1655.

(*E*)-6-Chloro-3-(pent-1-enyl)-2-phenyl-2*H*-indazole (2p)

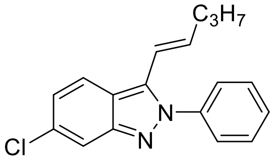

Eluent: Hexane/EtOAc (20:1). White solid (26 mg, 50% yield).

M.p. 53-54 ^{o}C.

^{1}H NMR (500 MHz, CDCl$_3$) δ (ppm) 7.79 – 7.77 (m, 1H), 7.72 – 7.71 (m, 1H), 7.61 – 7.47 (m, 5H), 7.09 (dt, *J* = 8.9, 1.6 Hz, 1H), 6.58 – 6.39 (m, 2H), 2.26 (q, *J* = 7.2 Hz, 2H), 1.53 (sext, *J* = 7.3 Hz, 2H), 0.97 (t, *J* = 7.1 Hz, 3H).

^{13}C {^{1}H} NMR (126 MHz, CDCl$_3$) δ (ppm) 149.1, 139.7, 137.1, 134.4, 132.9, 129.4, 129.1, 126.3, 123.7, 122.3, 118.7, 118.1, 116.9, 35.8, 22.4, 13.8.

HRMS (ESI+) m/z calculated for $C_{18}H_{18}ClN_2$ [M+H]$^+$: 297.1153, found [M+H]$^+$: 297.1162.

(*E*)-3-(Pent-1-enyl)-2-phenyl-5-(trifluoromethyl)-2*H*-indazole (2q)

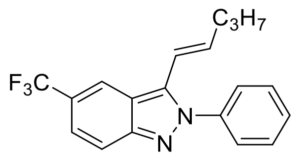

Eluent: Hexane/EtOAc (20:1). White solid (19 mg, 40% yield).

M.p. 55-56 ^{o}C.

^{1}H NMR (400 MHz, $CDCl_3$) δ (ppm) 8.17 (s, 1H), 7.83 (d, *J* = 9.2 Hz, 1H), 7.63 – 7.48 (m, 6H), 6.62 – 6.54 (m, 1H), 6.47 (d, *J* = 16.2 Hz, 1H), 2.29 (q, *J* = 7.2 Hz, 2H), 1.56 (sext, *J* = 7.6, 2H), 0.99 (t, *J* = 7.8 Hz, 3H).

^{19}F NMR (376 MHz, $CDCl_3$) δ (ppm) δ -61.68 (s).

^{13}C {^{1}H} NMR (101 MHz, $CDCl_3$) δ (ppm) 149.1, 139.2, 138.3, 136.0, 129.5, 129.4, 124.8 (q, *J* = 272.7 Hz), 124.2 (q, *J* = 31.9 Hz), 122.9 (q, *J* = 3.1 Hz), 119.8 (q, *J* = 4.9 Hz), 118.9, 118.9, 117.8, 35.9, 22.3, 13.8.

HRMS (ESI+) m/z calculated for $C_{19}H_{18}F_3N_2$ $[M+H]^+$: 331.1417, found $[M+H]^+$: 331.1418.

Methyl (*E*)-3-(pent-1-enyl)-2-phenyl-2*H*-indazole-5-carboxylate (2r)

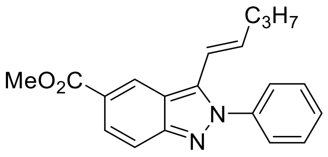

Eluent: Hexane/EtOAc (20:1). Yellow solid (33 mg, 65% yield).

M.p. 68-69 ^{o}C.

^{1}H NMR (300 MHz, $CDCl_3$) δ (ppm) 8.68 (dd, *J* = 1.6, 0.9 Hz, 1H), 7.95 (dd, *J* = 9.1, 1.6 Hz, 1H), 7.72 (dd, *J* = 9.1, 0.9 Hz, 1H), 7.64 – 7.45 (m, 5H), 6.66 (dt, *J* = 16.1, 6.9 Hz, 1H), 6.47 (dt, *J* = 16.1, 1.4 Hz, 1H), 3.96 (s, 3H), 2.33 – 2.24 (m, 2H), 1.57 (sext, *J* = 7.4 Hz, 2H), 0.98 (t, *J* = 7.4 Hz, 3H).

^{13}C {^{1}H} NMR (101 MHz, $CDCl_3$) δ (ppm) 167.6, 150.2, 139.7, 138.3, 136.4, 129.4, 129.2, 126.8, 126.3, 125.8, 124.1, 119.7, 117.9, 117.7, 52.2, 35.9, 22.3, 13.8.

HRMS (ESI+) m/z calculated for $C_{20}H_{21}N_2O_2$ $[M+H]^+$: 321.1598, found $[M+H]^+$: 321.1603.

(*E*)-5-Methyl-3-(pent-1-enyl)-2-phenyl-2*H*-indazole (2s)

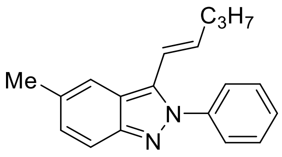

Eluent: Hexane/EtOAc (20:1). White solid (32 mg, 60% yield).

M.p. 49-50 ^{o}C.

^{1}H NMR (300 MHz, CDCl$_3$) δ (ppm) 7.67 – 7.45 (m, 7H), 7.19 (dd, *J* = 8.8, 1.6 Hz, 1H), 6.60 – 6.39 (m, 2H), 2.48 (s, 3H), 2.25 (td, *J* = 7.2, 5.6 Hz, 2H), 1.54 (sext, *J* = 7.3 Hz, 2H), 0.98 (t, *J* = 7.3 Hz, 3H).

^{13}C {^{1}H} NMR (101 MHz, CDCl$_3$) δ (ppm) 148.1, 140.3, 135.4, 132.8, 131.7, 129.7, 129.2, 128.7, 126.4, 120.4, 119.1, 118.7, 117.7, 35.9, 22.5, 22.1, 13.8.

HRMS (ESI+) m/z calculated for $C_{19}H_{21}N_2$ $[M+H]^+$: 277.1699, found $[M+H]^+$: 277.1702.

(*E*)-4-Methyl-3-(pent-1-enyl)-2-phenyl-2*H*-indazole (2t)

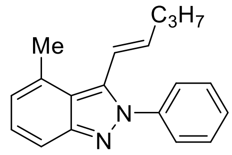

Eluent: Hexane/EtOAc (20:1). Yellow solid (8 mg, 16% yield).

M.p. 52-53 ^{o}C.

^{1}H NMR (400 MHz, CDCl$_3$) δ (ppm) 7.60 – 7.53 (m, 3H), 7.55 – 7.39 (m, 4H), 7.20 (dd, *J* = 8.7, 6.7 Hz, 1H), 6.83 (dt, *J* = 6.7, 1.1 Hz, 1H), 6.65 (dt, *J* = 16.1, 1.6 Hz, 1H), 5.72 (dt, *J* = 16.1, 7.1 Hz, 1H), 2.62 (s, 3H), 2.14 (qd, *J* = 7.1, 1.6 Hz, 2H), 1.39 (sext, *J* = 7.3 Hz, 2H), 0.87 (t, *J* = 7.3 Hz, 3H).

^{13}C {^{1}H} NMR (101 MHz, CDCl$_3$) δ (ppm) 149.2, 140.9, 140.6, 134.4, 131.7, 129.1, 128.5, 126.9, 126.6, 122.5, 121.0, 118.4, 115.6, 35.7, 22.1, 20.9, 13.8.

HRMS (ESI+) m/z calculated for $C_{19}H_{21}N_2$ $[M+H]^+$: 277.1699, found $[M+H]^+$: 277.1701.

(*E*)-3-(4-Methylpent-1-en-1-yl)-2-phenyl-2*H*-indazole (2u)

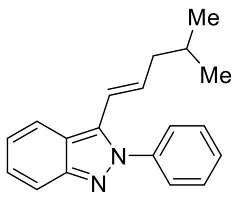

Eluent: Hexane/EtOAc (20:1). Yellow oil (41 mg, 76% yield).

^{1}H NMR (400 MHz, CDCl$_3$) δ (ppm) 7.86 (dt, *J* = 8.5, 1.1 Hz, 1H), 7.76 (dt, *J* = 8.8, 1.0 Hz, 1H), 7.64 – 7.46 (m, 5H), 7.35 (ddd, *J* = 8.7, 6.6, 1.1 Hz, 1H), 7.17 – 7.12 (m, 1H), 6.59 – 6.41 (m, 2H), 2.17 (t, *J* = 6.6 Hz, 2H), 1.80 (sept, *J* = 6.7 Hz, 1H), 0.98 (d, *J* = 6.7 Hz, 6H).

^{13}C {^{1}H} NMR (101 MHz, CDCl$_3$) δ 149.1, 140.2, 135.1, 133.8, 129.2, 128.8, 126.9, 126.4, 122.3, 121.0, 120.3, 119.4, 118.0, 43.2, 28.7, 22.5.

HRMS (ESI+) m/z calculated for $C_{19}H_{21}N_2$ [M+H]$^+$: 277.1699, found [M+H]$^+$: 277.1703.

(*E*)-3-(4-Chlorobut-1-enyl)-2-phenyl-2*H*-indazole (2v)

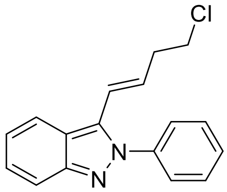

Eluent: Hexane/EtOAc (20:1). Yellow solid (29 mg, 59% yield).

M.p. 74-75 ^{o}C.

^{1}H NMR (400 MHz, CDCl$_3$) δ (ppm) 7.87 (dt, *J* = 8.5, 1.1 Hz, 1H), 7.77 (dt, *J* = 8.7, 1.1 Hz, 1H), 7.64 – 7.47 (m, 5H), 7.36 (ddd, *J* = 8.7, 6.6, 1.1 Hz, 1H), 7.18 (dd, *J* = 8.6, 6.6, 0.9 Hz, 1H), 6.65 – 6.42 (m, 2H), 3.67 (t, *J* = 6.6 Hz, 2H), 2.73 (q, *J* = 6.6 Hz, 2H).

^{13}C {^{1}H} NMR (101 MHz, CDCl$_3$) δ (ppm) 149.1, 139.9, 132.9, 130.5, 129.3, 129.0, 126.9, 126.4, 126.3, 122.7, 121.3, 120.8, 120.4, 118.2, 43.9, 36.6.

HRMS (ESI+) m/z calculated for $C_{17}H_{16}ClN_2$ [M+H]$^+$: 283.0997, found [M+H]$^+$: 283.0999.

(*E*)-3-(2-Cyclohexylvinyl)-2-phenyl-2*H*-indazole (2w)

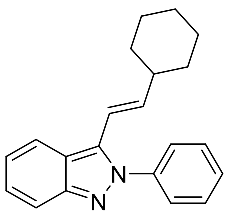

Eluent: Hexane/EtOAc (20:1). Yellow oil (52 mg, 91% yield).

^{1}H NMR (300 MHz, CDCl$_3$) δ (ppm) 7.87 (dd, *J* = 8.6, 1.0 Hz, 1H), 7.75 (d, *J* = 8.7 Hz, 1H), 7.70 – 7.40 (m, 5H), 7.34 (ddd, *J* = 8.9, 6.6, 1.1 Hz, 1H), 7.20 – 7.09 (m, 1H), 6.57 – 6.38 (m, 2H), 2.28 – 2.10 (m, 1H), 1.78 (m, 5H), 1.38 – 1.12 (m, 5H).

^{13}C {^{1}H} NMR (101 MHz, CDCl$_3$) δ (ppm) 149.1, 141.8, 140.2, 133.9, 129.7, 129.2, 128.8, 126.8, 126.3, 122.2, 121.1, 120.1, 118.0, 116.0, 41.9, 32.8, 26.2, 26.0.

HRMS (ESI+) m/z calculated for $C_{21}H_{23}N_2$ [M+H]$^+$: 303.1856, found [M+H]$^+$: 303,1857.

(*E*)-2-Phenyl-3-styryl-2*H*-indazole (2x)

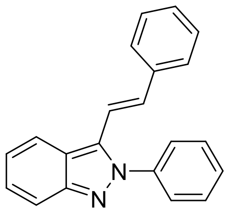

Eluent: Hexane/EtOAc (20:1). Yellow oil (50 mg, 80% yield).

^{1}H NMR (400 MHz, CDCl$_3$) δ (ppm) 8.04 (dt, *J* = 8.5, 1.1 Hz, 1H), 7.81 (dt, *J* = 8.6, 1.0 Hz, 1H), 7.68 – 7.62 (m, 2H), 7.60 – 7.52 (m, 3H), 7.50 – 7.45 (m, 2H), 7.44 – 7.33 (m, 4H), 7.32 – 7.28 (m, 1H), 7.25 – 7.15 (m, 2H),

^{13}C {^{1}H} NMR (101 MHz, CDCl$_3$) δ (ppm) 149.3, 140.0, 137.0, 133.5, 132.1, 129.4, 129.1, 129.0, 128.5, 127.1, 126.7, 126.5, 123.0, 121.0, 120.5, 118.3, 116.5.

HRMS (ESI+) m/z calculated for $C_{21}H_{17}N_2$ [M+H]$^+$: 297.1386, found [M+H]$^+$: 297.1387.

Derivatization of 2*H*-indazoles 2

(*E*)-2-[(1,1'-biphenyl)-3-yl]-3-(pent-1-en-1-yl)-2*H*-indazole (3)[3]

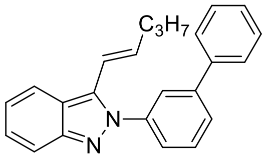

In a 5 mL Schlenk tube under an argon atmosphere, dimethyl (*E*)-2-(3-bromophenyl)-3-(pent-1-enyl)-2*H*-indazole (**2c**, 30 mg, 0.09 mmol) phenylboronic acid (0.18 mmol, 2 equiv.), $Pd(PPh_3)_4$ (0.0045 mmol, 5 mol %) and K_2CO_3 (0.18 mmol, 2 equiv.) were dissolved in a mixture of dioxane/H_2O (2:1, 0.1 M). The reaction was stirred and heated to 110 ^oC in a sand bath for 18 hours. Subsequently the mixture was cooled to room temperature, diluted with EtOAc (5 mL), washed with H_2O (2 x 5 mL), dried over $MgSO_4$, and filtered. The solvent was concentrated *in vacuo* and purified by column chromatography, using a mixture of hexane/EtOAc (20:1). (*E*)-2-([1,1'-biphenyl]-3-yl)-3-(pent-1-en-1-yl)-2*H*-indazole (**3**) was obtained as a yellow oil (22 mg, 0.07 mmol, 71% yield).

^{1}H NMR (300 MHz, $CDCl_3$) δ (ppm) 7.93 – 7.81 (m, 2H), 7.74 (dd, *J* = 14.1, 8.0 Hz, 2H), 7.69 – 7.52 (m, 4H), 7.47 (t, *J* = 7.4 Hz, 2H), 7.44 – 7.33 (m, 2H), 7.21 – 7.10 (m, 1H), 6.55 (d, *J* = 2.7 Hz, 2H), 2.27 (q, *J* = 7.1 Hz, 2H), 1.59 – 1.46 (m, 2H), 0.97 (t, *J* = 7.4 Hz, 4H).

^{13}C {^{1}H} NMR (126 MHz, $CDCl_3$) δ (ppm) 149.2, 142.6, 140.6, 140.0, 136.3, 134.0, 129.6, 129.1, 128.1, 127.5, 127.4, 127.0, 125.1, 125.1, 122.4, 121.0, 120.3, 118.6, 118.0, 35.9, 22.6 , 13.8.

HRMS (ESI+) m/z calculated for $C_{24}H_{23}N_2$ $[M+H]^+$: 339.1856, found $[M+H]^+$: 339.1858.

(*E*)-3-[3-(Pent-1-en-1-yl)-2*H*-indazol-2-yl]aniline (4)[4]

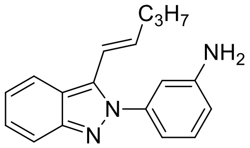

In a 5 mL Schlenk tube under an argon atmosphere, (*E*)-2-(3-nitrophenyl)-3-(pent-1-en-1-yl)-2*H*-indazole (**2g**, 20 mg, 0.07 mmol) NH_4Cl (0.035 mmol, 0.5 equiv.) and Fe powder (0.70 mmol, 10 equiv.) were suspended in EtOH/H_2O (4:1, 0.1 M). The reaction was stirred and heated at 80 ^oC in a sand bath for 3 hours. Subsequently, the mixture was cooled to room temperature, diluted with EtOAc (5 mL), washed with H_2O (2 x 5 mL), dried over $MgSO_4$, and filtered through Celite.

The solvent was concentrated *in vacuo* and (*E*)-3-[3-(pent-1-en-1-yl)-2*H*-indazol-2-yl]aniline (**4**) was obtained as a yellow oil (16 mg, 0.06 mmol, 88% yield).

^{1}H NMR (300 MHz, $CDCl_3$) δ (ppm) 7.85 (d, *J* = 8.5 Hz, 1H), 7.73 (d, *J* = 8.7 Hz, 1H), 7.38 – 7.22 (m, 2H), 7.18 – 7.07 (m, 1H), 6.97 – 6.86 (m, 2H), 6.78 – 6.75 (m, 1H), 6.54 – 6.51 (m, 2H), 3.86 (s, 2H), 2.28 – 2.22 (m, 2H), 1.53 (sext, *J* = 7.3 Hz, 2H), 0.98 (t, *J* = 7.3 Hz, 3H).

^{13}C {^{1}H} NMR (75 MHz, $CDCl_3$) δ (ppm) 148.9, 147.5, 141.1, 135.7, 133.7, 129.9, 126.7, 122.2, 121.0, 120.1, 118.8, 118.0, 116.4, 115.3, 112.8, 35.9, 22.6, 13.8.

HRMS (ESI+) m/z calculated for $C_{18}H_{20}N_3$ $[M+H]^+$: 278.1652, found $[M+H]^+$: 278.1653.

(*E*)-4-[3-(Pent-1-en-1-yl)-2*H*-indazol-2-yl]phenol (5)[5]

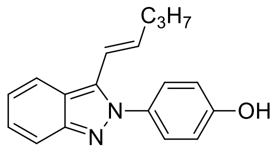

The corresponding dimethyl (*E*)-2-(4-methoxyphenyl)-3-(pent-1-enyl)-2*H*-indazole (**2o**, 31 mg, 0.11 mmol) was dissolved in DCM (0.2 M) within a 5 mL Schlenk tube under an argon atmosphere. Subsequently, BBr_3 (1.10 mmol, 10 equiv.) was added dropwise at 0 ^oC, and the reaction was stirred for 30 minutes. The resulting mixture was quenched with MeOH at 0 ^oC and the solvents were evaporated under vacuum. The crude mixture was dissolved in DCM (5 mL), washed with a saturated aqueous $NaHCO_3$ solution (2 × 5 mL), dried over $MgSO_4$, and filtered. The solvent was then concentrated *in vacuo* and the product was purified by column chromatography, using a Hex/AcOEt mixture (4:1). (*E*)-4-[3-(pent-1-en-1-yl)-2*H*-indazol-2-yl]phenol (**5**) was obtained as a yellow oil (28 mg, 0.10 mmol, 95% yield).

^{1}H NMR (300 MHz, $CDCl_3$) δ (ppm) 9.83 (s, 1H), 7.88 (d, *J* = 8.4 Hz, 1H), 7.74 (d, *J* = 8.6 Hz, 1H), 7.42 – 7.32 (m, 1H), 7.26 (d, *J* = 8.4 Hz, 2H), 7.17 (dd, *J* = 8.5, 6.6 Hz, 1H), 6.79 (d, *J* = 8.4 Hz, 2H), 6.58 (dt, *J* = 16.0, 6.8 Hz, 1H), 6.40 (d, *J* = 16.2 Hz, 1H), 2.23 (q, *J* = 7.1 Hz, 2H), 1.52 (sext, *J* = 7.4 Hz, 2H), 0.96 (t, *J* = 7.3 Hz, 3H).

^{13}C {^{1}H} NMR (75 MHz, $CDCl_3$) δ (ppm) 158.2, 148.2, 137.0, 134.7, 131.0, 127.7, 127.5, 122.4, 121.1, 119.7, 118.2, 117.0, 116.4, 35.9, 22.5, 13.8.

HRMS (ESI+) m/z calculated for $C_{18}H_{19}N_2O$ $[M+H]^+$: 279.1492, found $[M+H]^+$: 279.1503.

(*E*)-3-(Pent-1-en-1-yl)-2-phenyl-2*H*-indazole-5-carboxylic acid (6)[6]

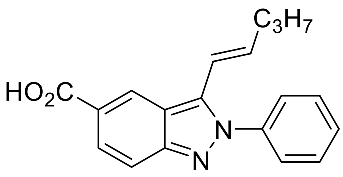

Methyl (*E*)-3-(pent-1-enyl)-2-phenyl-2*H*-indazole-5-carboxylate (**2r**, 29 mg, 0.09 mmol, 1 equiv.) was dissolved in THF/MeOH (3:1, 0.25 M) within a 5 mL Schlenk tube. Subsequently, an aqueous solution of KOH (30% w/v, 0.03 mL) was added dropwise at room temperature. The reaction was stirred and heated at 50 ^{o}C in a sand bath for 18 hours. Following this, the mixture was cooled to room temperature, diluted with H_2O (5 mL), and washed with Et_2O (2 x 5 mL). The aqueous layer was acidified with 1 M aq. HCl, extracted with DCM, dried over $MgSO_4$, and filtered. The solvent was concentrated *in vacuo*, yielding (*E*)-3-(pent-1-en-1-yl)-2-phenyl-2*H*-indazole-5-carboxylic acid (**6**) as a yellow oil (28 mg, 0.09 mmol, 100% yield).

^{1}H NMR (300 MHz, CD_3COCD_3) δ (ppm) 8.72 (t, *J* = 1.2 Hz, 1H), 7.94 (dd, *J* = 9.1, 1.5 Hz, 1H), 7.74 – 7.61 (m, 5H), 7.62 – 7.54 (m, 1H), 6.71 (dt, *J* = 16.0, 6.8 Hz, 1H), 6.59 (dt, *J* = 16.2, 1.4 Hz, 1H), 2.32 (q, *J* = 7.1 Hz, 2H), 1.57 (sext, *J* = 7.4 Hz, 2H), 0.98 (t, *J* = 7.3 Hz, 3H).

^{13}C {^{1}H} NMR (126 MHz, CD_3COCD_3) δ (ppm) 168.0, 150.8, 140.8, 138.6, 136.7, 130.1, 130.0, 127.3, 127.1, 126.4, 125.1, 120.4, 118.7, 118.4, 36.3, 22.8, 13.9.

HRMS (ESI+) m/z calculated for $C_{19}H_{19}N_2O_2$ $[M+H]^+$: 307.1441, found $[M+H]^+$: 307.1440.

5-Phenylindazolo[2,3-*a*]quinoline (7)

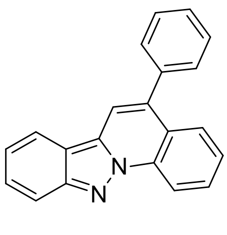

(*E*)-2-Phenyl-3-styryl-2*H*-indazole **2x** (0.16 mmol, 1 equiv.) was dissolved in MeCN (0.1 M) in a Schlenk tube (5 mL). The reaction mixture was irradiated with blue LED chips (2 x 50 W) at 70 ^{o}C for 24 hours. DDQ (0.19 mmol, 1.2 equiv.) was then added, and the reaction mixture was irradiated with blue LED Kessil (2 x 40 W) at 50 ^{o}C until completion. The mixture was diluted with EtOAc and washed with $NaHCO_3$ (3 x 10 mL). The organic layer was dried over $MgSO_4$ and filtered. The solvents were removed under reduced pressure and the crude was purified by

column chromatography using a mixture of hexane/EtOAc (50:1) as eluent to yield the corresponding indazolo[2,3-*a*]quinoline **6** as a yellow solid (18 mg, 45% yield).

M.p. 145-146 ^{0}C.

^{1}H NMR (500 MHz, $CDCl_3$) δ (ppm) 9.05 (dd, *J* = 8.5, 1.4 Hz, 1H), 8.07 (dt, *J* = 8.3, 1.1 Hz, 1H), 8.01 – 7.93 (m, 3H), 7.82 (ddd, *J* = 8.4, 7.0, 1.4 Hz, 1H), 7.62 – 7.50 (m, 7H), 7.28 – 7.25 (m, 1H).

^{13}C {^{1}H} NMR (126 MHz, $CDCl_3$) δ (ppm) 149.5, 138.7, 136.0, 134.3, 132.1, 130.0, 129.6, 128.8, 128.3, 128.3, 127.5, 126.1, 124.6, 120.9, 119.9, 117.6, 117.1, 116.7, 115.8.

HRMS (ESI+) m/z calculated for $C_{21}H_{15}N_2$ $[M+H]^+$: 295.1230, found $[M+H]^+$: 295.1233.

X-Ray crystallographic data

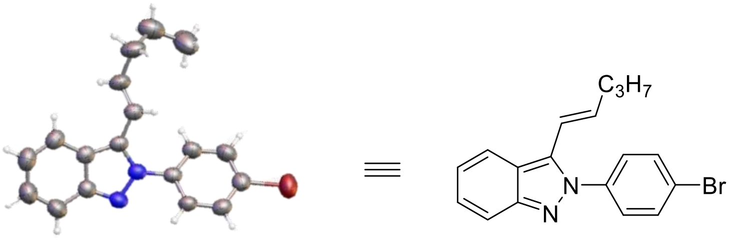

Molecular structure of **2b**: ellipsoid contours of probability levels are 50%.

X-Ray Crystallographic Data for **2b** (CCDC number: 2374982)

Empirical formula	$C_{18}H_{17}BrN_2$
Formula weight	341.24
Temperature/K	300.0
Crystal system	triclinic
Space group	P-1
a/Å	7.8419(4)
b/Å	9.8788(5)
c/Å	12.0913(5)
α/°	68.302(2)
β/°	79.259(2)
γ/°	68.100(2)
Volume/Å^3	806.24(7)
Z	2
ρ_{calc}g/cm^3	1.406
μ/mm^{-1}	2.544
F(000)	348.0
Crystal size/mm^3	0.1 × 0.08 × 0.05
Radiation	MoKα (λ = 0.71073)
2Θ range for data collection/°	4.908 to 61.09
Index ranges	$-11 \leq h \leq 11$, $-14 \leq k \leq 14$, $-17 \leq l \leq 17$
Reflections collected	23327
Independent reflections	4881 [R_{int} = 0.0652, R_{sigma} = 0.0627]
Data/restraints/parameters	4881/0/191
Goodness-of-fit on F^2	1.010
Final R indexes [I>=2σ (I)]	R_1 = 0.0569, wR_2 = 0.1195
Final R indexes [all data]	R_1 = 0.1379, wR_2 = 0.1488
Largest diff. peak/hole / e Å^{-3}	0.50/-0.47

DFT Calculations

All reported structures were optimized at Density Functional Theory level as implemented in Gaussian 16.[7] The geometry optimizations were performed using M06 functional[8] with a mixed basis set of LANL2DZ[9] for Cu and 6-31+G(d,p) basis set for all other atoms. Solvent effects were considered in all the calculations applying the solvation model based on density (SMD)10 using toluene as solvent at 393.15 K. Reported energy values correspond to Gibbs Free (G) energies in kcal·mol^{-1}. All structures were optimized without geometrical constraint. Stationary points were characterized by frequency calculations (no negative frequency for minima and one negative frequency for transition states).

CARTESIAN COORDINATES OF THE COMPUTED STRUCTURES

$Cu(MeCN)_4^+$

N	-0.94572200	-0.23842800	-1.80066300
N	-0.21949100	-1.70448000	1.11990600
C	-1.48703600	-0.37567300	-2.81481500
C	-0.35316700	-2.66997900	1.74464100
C	-0.52149500	-3.87667600	2.52455000
C	-2.16387300	-0.54752800	-4.08173400
Cu	0.00654800	0.00270800	0.00200200
N	2.00223700	0.35729400	-0.31539800
N	-0.82246800	1.59110400	1.00349800
C	3.12888300	0.55687400	-0.49199400
C	4.53669400	0.80622900	-0.71226900
C	-1.29305900	2.48722200	1.56552200
C	-1.88126400	3.60698800	2.26754400
H	0.30327500	-4.56691000	2.32823600
H	-1.46331200	-4.36439700	2.25927200
H	-0.53529100	-3.63419300	3.59048400
H	-2.95805100	0.19673400	-4.18513800
H	-2.60420500	-1.54667300	-4.13766300
H	-1.45411200	-0.42582300	-4.90433900
H	5.09340200	0.65099100	0.21581800
H	4.92195900	0.12484500	-1.47555100
H	4.68579700	1.83603700	-1.04781800
H	-1.34808400	4.52769300	2.01571400
H	-2.93153000	3.71596900	1.98389000
H	-1.81995500	3.44523600	3.34710900

SCF Done: E(RM06) = -726.774088583
Zero-point correction= 0.185663 (Hartree/Particle)
Thermal correction to Energy= 0.216327
Thermal correction to Enthalpy= 0.217541
Thermal correction to Gibbs Free Energy= 0.103082
Sum of electronic and zero-point Energies= -726.588425
Sum of electronic and thermal Energies= -726.557761
Sum of electronic and thermal Enthalpies= -726.556548
Sum of electronic and thermal Free Energies= -726.671007

MeCN

N	-1.43606300	-0.00003700	0.00001900
C	-0.27577700	0.00009100	-0.00004500
C	1.17449000	0.00001600	-0.00000800
H	1.55357000	1.00392500	-0.20899800
H	1.55326500	-0.32108300	0.97403800
H	1.55333300	-0.68322200	-0.76485500

SCF Done: E(RM06) = -132.665054392
Zero-point correction= 0.045217 (Hartree/Particle)
Thermal correction to Energy= 0.050308
Thermal correction to Enthalpy= 0.051521
Thermal correction to Gibbs Free Energy= 0.012883
Sum of electronic and zero-point Energies= -132.619837
Sum of electronic and thermal Energies= -132.614746
Sum of electronic and thermal Enthalpies= -132.613533
Sum of electronic and thermal Free Energies= -132.652171

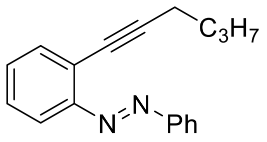

1a

C	-1.10193400	-2.13088200	0.01589100
C	1.03700600	-3.24768300	-0.06798000
C	0.40464900	-4.48101600	-0.12361900
C	-0.98592600	-4.55367700	-0.09161200
C	-1.72224900	-3.38351900	-0.00686900
H	2.12281000	-3.18851900	-0.07160000
H	1.00065700	-5.38831900	-0.17965600
H	-1.48974000	-5.51581600	-0.12403800
H	-2.80873100	-3.39868900	0.03224100
C	-2.42998000	1.14824500	-0.11715000
C	-1.90821900	2.38969800	-0.48482800
C	-3.75402900	1.04613100	0.32590400
C	-2.70006000	3.52850300	-0.41380500
H	-0.87446300	2.42921600	-0.82147100
C	-4.53966900	2.18520100	0.39269400
H	-4.14018100	0.07101200	0.60879000
C	-4.01671700	3.42640400	0.02436700
H	-2.29214000	4.49454900	-0.69977900
H	-5.56928400	2.11238900	0.73488500
H	-4.64099500	4.31483900	0.08120800
N	-2.00191600	-1.05005000	0.09728700
N	-1.52852700	0.06469100	-0.23165500
C	0.31263200	-2.04739400	-0.00039200
C	1.06032000	-0.83856600	0.09949800
C	1.81096700	0.10816300	0.20464600
C	2.69418400	1.25828900	0.31807400
H	2.35466600	2.05457900	-0.36260700
H	2.61097900	1.68198100	1.33087500
C	4.15883200	0.92973200	0.02634200
H	4.24136500	0.50731000	-0.98582300
H	4.49732300	0.14108400	0.71426800
C	5.05850700	2.14834500	0.15265800
H	4.96057500	2.56665900	1.16589700
H	4.70281200	2.93297700	-0.53221700
C	6.51410900	1.82650700	-0.13792100
H	7.15486700	2.70950300	-0.04452700
H	6.89382600	1.06437100	0.55390100
H	6.63420400	1.43350100	-1.15516100

SCF Done: E(RM06) = -805.574257390
Zero-point correction= 0.312401 (Hartree/Particle)
Thermal correction to Energy= 0.342085
Thermal correction to Enthalpy= 0.343298
Thermal correction to Gibbs Free Energy= 0.239585
Sum of electronic and zero-point Energies= -805.261857
Sum of electronic and thermal Energies= -805.232173
Sum of electronic and thermal Enthalpies= -805.230959

Sum of electronic and thermal Free Energies= -805.334672

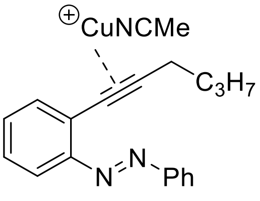

A

C	-1.29136900	-1.86852100	-0.35870800
C	-0.05682800	-1.93100800	-0.26907500
Cu	-0.39399300	0.05495700	-0.26788100
N	-1.83785900	1.38282200	-0.29595700
C	-2.71111300	2.14259500	-0.31199600
C	-3.79604300	3.09571300	-0.33361000
H	-4.59660000	2.73416700	-0.98542800
H	-4.19102600	3.23001500	0.67748600
H	-3.43748800	4.05909100	-0.70673300
C	-2.74858200	-1.96093500	-0.38696500
H	-3.14295800	-1.26828300	-1.14308900
H	-3.02574300	-2.97201700	-0.71813600
C	-3.37415500	-1.67480400	0.98473200
H	-2.99251500	-0.71267000	1.36077800
H	-3.03135200	-2.44223000	1.68978800
C	-4.89679600	-1.63779700	0.93087500
H	-5.28610900	-1.68961200	1.95528300
H	-5.26623300	-2.53995500	0.42165700
C	-5.44347700	-0.39172800	0.25076500
H	-6.53787300	-0.38994100	0.23498000
H	-5.11957000	0.51082100	0.78763100
H	-5.10590400	-0.30259900	-0.79035800
C	1.28510200	-2.41449400	-0.17730800
C	1.45412600	-3.80232500	-0.25249000
C	2.43510700	-1.60240600	0.00707700
C	2.70961200	-4.38646800	-0.15024300
H	0.57133600	-4.42124400	-0.39066700
C	3.69221300	-2.21840600	0.11880400
C	3.83785900	-3.59198500	0.03767600
H	2.80314900	-5.46707900	-0.21335100
H	4.54883400	-1.56640800	0.26498200
H	4.82221400	-4.04242600	0.12208400
N	2.53735900	-0.21396400	0.09432600
N	1.55352800	0.54778900	-0.08894200
C	1.86760500	1.93119000	0.05321700
C	1.12734600	2.84420900	-0.69913000
C	2.87525100	2.36951900	0.91647700
C	1.40901000	4.20064700	-0.60187600
H	0.36204100	2.48138900	-1.38231700
C	3.13296800	3.72791700	1.02268500
H	3.42911900	1.64135900	1.50156900
C	2.40574300	4.64413100	0.26332900
H	0.85077500	4.91282400	-1.20355800
H	3.90351200	4.07725300	1.70469100
H	2.61551500	5.70700000	0.34985800

SCF Done: E(RM06) = -1134.31847705

Zero-point correction= 0.362157 (Hartree/Particle)
Thermal correction to Energy= 0.400593
Thermal correction to Enthalpy= 0.401806
Thermal correction to Gibbs Free Energy= 0.280648
Sum of electronic and zero-point Energies= -1133.956320
Sum of electronic and thermal Energies= -1133.917884
Sum of electronic and thermal Enthalpies= -1133.916671
Sum of electronic and thermal Free Energies= -1134.037829

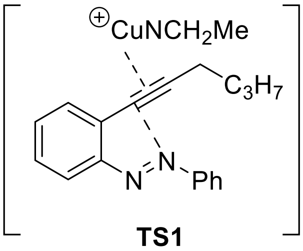

TS1

C	0.17757700	2.07672800	0.28971100
C	-0.93431400	2.86538800	-0.07240500
C	-0.82323900	4.24276300	-0.23868400
C	0.40028900	4.85791800	-0.01891500
C	1.50383700	4.09358600	0.36407300
C	1.39809300	2.71748100	0.52615800
C	-0.00161600	0.66509100	0.37454400
H	-1.70805000	4.80526100	-0.52337400
H	0.49829000	5.93296500	-0.13708000
H	2.46090500	4.57675200	0.54073300
H	2.26591500	2.13399900	0.83230800
N	-2.16945300	2.24008600	-0.24464300
N	-2.14185800	1.05857900	0.17110700
C	-3.29756700	0.27669700	-0.01080900
C	-4.24258700	0.55146000	-1.00606400
C	-3.47083200	-0.80094700	0.86161900
C	-5.35666000	-0.26453300	-1.12149100
H	-4.07927400	1.39061700	-1.67650400
C	-4.59805700	-1.60264200	0.74669600
H	-2.73190600	-0.97111900	1.64135100
C	-5.53666200	-1.33818400	-0.24762100
H	-6.09028600	-0.06862100	-1.89887200
H	-6.41360000	-1.97264700	-0.34627700
C	0.35288900	-0.55548800	0.40034800
Cu	2.26376700	-0.37675900	0.09136500
N	4.12328500	-0.30489900	-0.21301100
C	5.26580200	-0.29660700	-0.39587100
C	6.69041000	-0.28646300	-0.62288700
H	6.91087800	0.20920600	-1.57226700
H	7.19091700	0.25028400	0.18774500
H	7.06590100	-1.31284800	-0.65851100
H	-4.74580200	-2.43270600	1.43184100
C	-0.23395900	-1.91320900	0.54234700
H	-0.43357400	-2.07818500	1.61307600
H	-1.21706300	-1.92094800	0.04671000
C	0.63242000	-3.04721500	0.00893200
H	0.86124900	-2.86517200	-1.05301200
H	1.59951300	-3.05007800	0.53990600
C	-0.03437900	-4.40518700	0.16223500
H	-0.26861300	-4.57383000	1.22405400
H	-1.00109300	-4.39169700	-0.36284700

C	0.82940100	-5.53685600	-0.36715500
H	1.78765600	-5.58327000	0.16522500
H	0.33953300	-6.50899300	-0.25379400
H	1.05036600	-5.39801900	-1.43275400

SCF Done: E(RM06) = -1134.28933223
Zero-point correction= 0.360155 (Hartree/Particle)
Thermal correction to Energy= 0.398423
Thermal correction to Enthalpy= 0.399637
Thermal correction to Gibbs Free Energy= 0.275493
Sum of electronic and zero-point Energies= -1133.929178
Sum of electronic and thermal Energies= -1133.890909
Sum of electronic and thermal Enthalpies= -1133.889696
Sum of electronic and thermal Free Energies= -1134.013839
Frequency= -197.5468

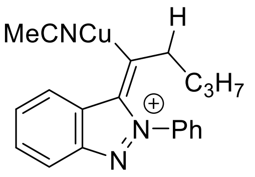

B

C	0.20787600	2.15112700	-0.13604800
C	-0.85373800	3.06519000	-0.32095700
C	-0.64592300	4.43435200	-0.55905500
C	0.65972800	4.86602000	-0.60532200
C	1.73003000	3.96120200	-0.42281600
C	1.52549700	2.61194000	-0.19565400
C	-0.41656200	0.86702700	0.03918000
H	-1.48923900	5.10409900	-0.69794200
H	0.87937800	5.91462600	-0.78396700
H	2.74722100	4.34261700	-0.46197000
H	2.37259000	1.94023200	-0.06332000
N	-2.05559400	2.43849400	-0.21303300
N	-1.80315000	1.17888200	-0.00835300
C	-2.90961800	0.31052600	0.26483500
C	-3.88518400	0.14015900	-0.71074900
C	-2.99150800	-0.31030200	1.50670000
C	-4.95594200	-0.70439600	-0.44119500
H	-3.79068500	0.65543600	-1.66292600
C	-4.06726300	-1.15081400	1.76200100
H	-2.22534400	-0.12805600	2.25717200
C	-5.04297200	-1.35268400	0.78799500
H	-5.72333000	-0.85660400	-1.19494500
H	-5.88104200	-2.01364500	0.99199000
C	0.18047900	-0.37793800	0.06420300
Cu	2.07907500	-0.52030700	0.22439600
N	3.95403300	-0.68654900	0.41010300
C	5.09892500	-0.80611700	0.52898500
C	6.52635900	-0.95557300	0.67765900
H	7.01114800	0.02210900	0.60834400
H	6.75223300	-1.39956500	1.65107900
H	6.91479100	-1.60549800	-0.11126500
H	-4.14939500	-1.64183200	2.72767100
C	-0.56490400	-1.66246000	-0.03751300
H	-0.81602800	-1.96391200	0.99702000
H	-1.53594700	-1.56022800	-0.54567600
C	0.23780100	-2.79059600	-0.68076600
H	0.56266000	-2.48318600	-1.68702700
H	1.15989500	-2.96101300	-0.09893700
C	-0.55202600	-4.08711300	-0.77198700
H	-0.88218900	-4.37945100	0.23634700
H	-1.47067200	-3.91009600	-1.35079600
C	0.25256000	-5.20967200	-1.40418800
H	1.15887800	-5.42065800	-0.82283300
H	-0.32457000	-6.13755500	-1.46750800
H	0.56776600	-4.94397500	-2.42087100

SCF Done: E(RM06) = -1134.33105100
Zero-point correction= 0.362959 (Hartree/Particle)
Thermal correction to Energy= 0.400763
Thermal correction to Enthalpy= 0.401976
Thermal correction to Gibbs Free Energy= 0.279230
Sum of electronic and zero-point Energies= -1133.968092
Sum of electronic and thermal Energies= -1133.930288
Sum of electronic and thermal Enthalpies= -1133.929075
Sum of electronic and thermal Free Energies= -1134.051821

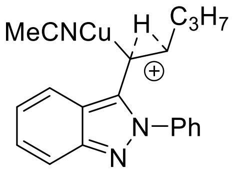

TS2

C	0.35679400	2.11280400	-0.57622200
C	-0.58376900	3.14576700	-0.29881500
C	-0.24661900	4.50517600	-0.49053400
C	1.01300900	4.78566300	-0.95299200
C	1.95288700	3.75507300	-1.23724100
C	1.64298900	2.42845900	-1.06540300
C	-0.33014700	0.92385200	-0.29053600
H	-0.97181200	5.28523400	-0.27695900
H	1.30904400	5.81919400	-1.11328200
H	2.93716000	4.03380900	-1.60521900
H	2.36352500	1.64752300	-1.30605300
N	-1.74747400	2.64427800	0.15592500
N	-1.57465900	1.31873500	0.13962800
C	-2.61117800	0.49389100	0.67236900
C	-3.91291500	0.67870200	0.21846400
C	-2.30889400	-0.47076600	1.63087500
C	-4.92443800	-0.13079400	0.72230900
H	-4.11551400	1.45224300	-0.51710400
C	-3.32755800	-1.28309900	2.11667800
H	-1.29074000	-0.56848400	2.00293900
C	-4.63308100	-1.11628200	1.66195800
H	-5.94463200	0.00689900	0.37441600
H	-5.42707400	-1.74962300	2.04849500
C	0.13664700	-0.44503000	-0.43368700
Cu	1.92734800	-0.76897000	0.20743000
N	3.66046900	-1.01998300	0.89644500
C	4.72450000	-1.15690500	1.33000300
C	6.05149500	-1.32788400	1.86955700
H	6.27025900	-0.52203200	2.57559300

H	6.11959900	-2.28803700	2.38837200
H	6.78593400	-1.30458700	1.05971700
H	-3.10233600	-2.03582600	2.86756800
C	-0.74292700	-1.45269600	-0.85720900
H	-1.76880400	-1.18817500	-1.14798900
H	0.23616800	-0.79231200	-1.62180500
C	-0.40304600	-2.89460500	-0.92050400
H	0.67560600	-3.03458400	-0.74741900
H	-0.90870900	-3.34641700	-0.04847000
C	-0.86775500	-3.59263400	-2.19499000
H	-1.94273800	-3.41222100	-2.33461700
H	-0.36438700	-3.13529200	-3.05875900
C	-0.58473300	-5.08380400	-2.15166100
H	-1.10565400	-5.55937900	-1.31202000
H	-0.91145500	-5.57888100	-3.07062700
H	0.48756100	-5.27946200	-2.03076000

SCF Done: E(RM06) = -1134.29811706
Zero-point correction= 0.358980 (Hartree/Particle)
Thermal correction to Energy= 0.396530
Thermal correction to Enthalpy= 0.397743
Thermal correction to Gibbs Free Energy= 0.275192
Sum of electronic and zero-point Energies= -1133.939137
Sum of electronic and thermal Energies= -1133.901587
Sum of electronic and thermal Enthalpies= -1133.900374
Sum of electronic and thermal Free Energies= -1134.022925
Frequency= -841.6362

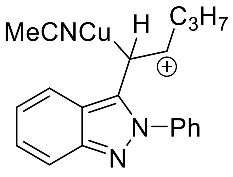

C

C	-2.81372900	-0.69926100	0.36531300
C	-3.54297400	0.30977900	-0.32887900
C	-4.90672500	0.12773100	-0.65444700
C	-5.49357500	-1.05237000	-0.27948800
C	-4.76813700	-2.06043300	0.41565400
C	-3.44568200	-1.90174300	0.74785700
C	-1.52637800	-0.15852700	0.52142500
H	-5.45273000	0.90575900	-1.18027400
H	-6.54034300	-1.23022700	-0.51205400
H	-5.28323000	-2.97709900	0.69139500
H	-2.90900300	-2.67815900	1.28879000
N	-2.77152400	1.37997000	-0.60430300
N	-1.58345200	1.07656200	-0.07717200
C	-0.46484700	1.93586900	-0.29943600
C	-0.23504300	3.01105000	0.54773200
C	0.42636500	1.61011800	-1.32141200
C	0.93639600	3.74861000	0.39520900
H	-0.95706100	3.24373700	1.32637100
C	1.60382500	2.34446000	-1.45481600
H	0.16913700	0.81782200	-2.02541500
C	1.86233300	3.40308700	-0.58646400
H	1.13156300	4.59015300	1.05411700
H	2.78039400	3.97510800	-0.68917000

C	-0.39372100	-0.74018600	1.20371700
Cu	1.25085900	-0.68231400	-0.23974300
N	2.19192000	-1.32992100	-1.77405800
C	2.76574900	-1.75035400	-2.68686000
C	3.47885000	-2.27786600	-3.82443900
H	2.92904500	-3.12561800	-4.24274100
H	3.58272900	-1.50419300	-4.59006300
H	4.47263500	-2.61302300	-3.51475500
H	2.30686600	2.09844700	-2.24702000
C	0.74104000	-0.14489800	1.71533100
H	0.86003200	0.94085100	1.64881200
C	1.71076900	-0.85536100	2.61120600
H	1.53904200	-0.50281400	3.64113800
H	1.50478800	-1.93601900	2.61595800
C	3.17039200	-0.59559500	2.24447400
H	3.37277700	-1.00760200	1.24015200
H	3.33388700	0.49001000	2.16427900
C	4.13278900	-1.20377300	3.24928300
H	3.98831700	-2.28816400	3.32641100
H	5.17534900	-1.02418500	2.97022500
H	3.97543500	-0.77773800	4.24733300
H	-0.53933400	-1.79825500	1.44201600

SCF Done: E(RM06) = -1134.36286548
Zero-point correction= 0.363031 (Hartree/Particle)
Thermal correction to Energy= 0.401090
Thermal correction to Enthalpy= 0.402303
Thermal correction to Gibbs Free Energy= 0.277032
Sum of electronic and zero-point Energies= -1133.999834
Sum of electronic and thermal Energies= -1133.961775
Sum of electronic and thermal Enthalpies= -1133.960562
Sum of electronic and thermal Free Energies= -1134.085834

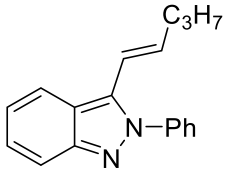

2a

C	2.39276000	1.17133800	0.19332600
C	1.07098200	1.63821700	-0.07032100
C	0.82177100	3.02159500	-0.21708600
C	1.87302400	3.88922600	-0.06290000
C	3.18466000	3.42100800	0.23141100
C	3.46147900	2.08498000	0.35580300
C	0.29563600	0.47323600	-0.17398800
H	-0.17286000	3.38886100	-0.45929600
H	1.70945300	4.95865500	-0.17097500
H	3.98464400	4.14837300	0.34947700
H	4.46344100	1.72037800	0.56632900
C	0.88969600	-1.94892800	0.03291900
C	-0.25127200	-2.43576100	0.66698900
C	1.79048700	-2.81789200	-0.57885400
C	-0.50135600	-3.80350000	0.66214700

H	-0.92433200	-1.75012900	1.17493900
C	1.53474400	-4.18216200	-0.56777600
H	2.67954800	-2.40923100	-1.04970100
C	0.38718400	-4.67812600	0.04585300
H	-1.39001500	-4.18582000	1.15757100
H	2.23478100	-4.86207400	-1.04642800
H	0.18950900	-5.74689100	0.04889000
N	2.44962500	-0.16850400	0.24996500
N	1.17477700	-0.55596100	0.02724800
C	-1.09866000	0.28699100	-0.51925800
H	-1.35888300	-0.63860100	-1.03761400
C	-2.06543200	1.17833500	-0.26281800
H	-1.82043400	2.08011200	0.30444400
C	-3.49304600	1.01306000	-0.65758700
H	-3.78288700	1.82231200	-1.34807200
H	-3.62108200	0.07302000	-1.21494100
C	-4.44180600	1.04021600	0.53990700
H	-4.17270300	0.22659700	1.22835900
H	-4.28696100	1.97392500	1.10039500
C	-5.89646100	0.91755400	0.12180500
H	-6.07267200	-0.02006000	-0.41992400
H	-6.57132100	0.93569700	0.98375900
H	-6.18438300	1.73980900	-0.54514100

SCF Done: E(RM06) = -805.648616050
Zero-point correction= 0.315509 (Hartree/Particle)
Thermal correction to Energy= 0.343404
Thermal correction to Enthalpy= 0.344617
Thermal correction to Gibbs Free Energy= 0.248927
Sum of electronic and zero-point Energies= -805.333107
Sum of electronic and thermal Energies= -805.305212
Sum of electronic and thermal Enthalpies= -805.303999
Sum of electronic and thermal Free Energies= -805.399689

NMR spectra

(*E*)-3-(Pent-1-enyl)-2-phenyl-2*H*-indazole (2a)

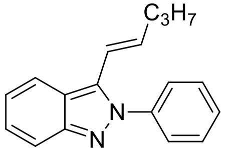

^{1}H-NMR (300 MHz, $CDCl_3$)

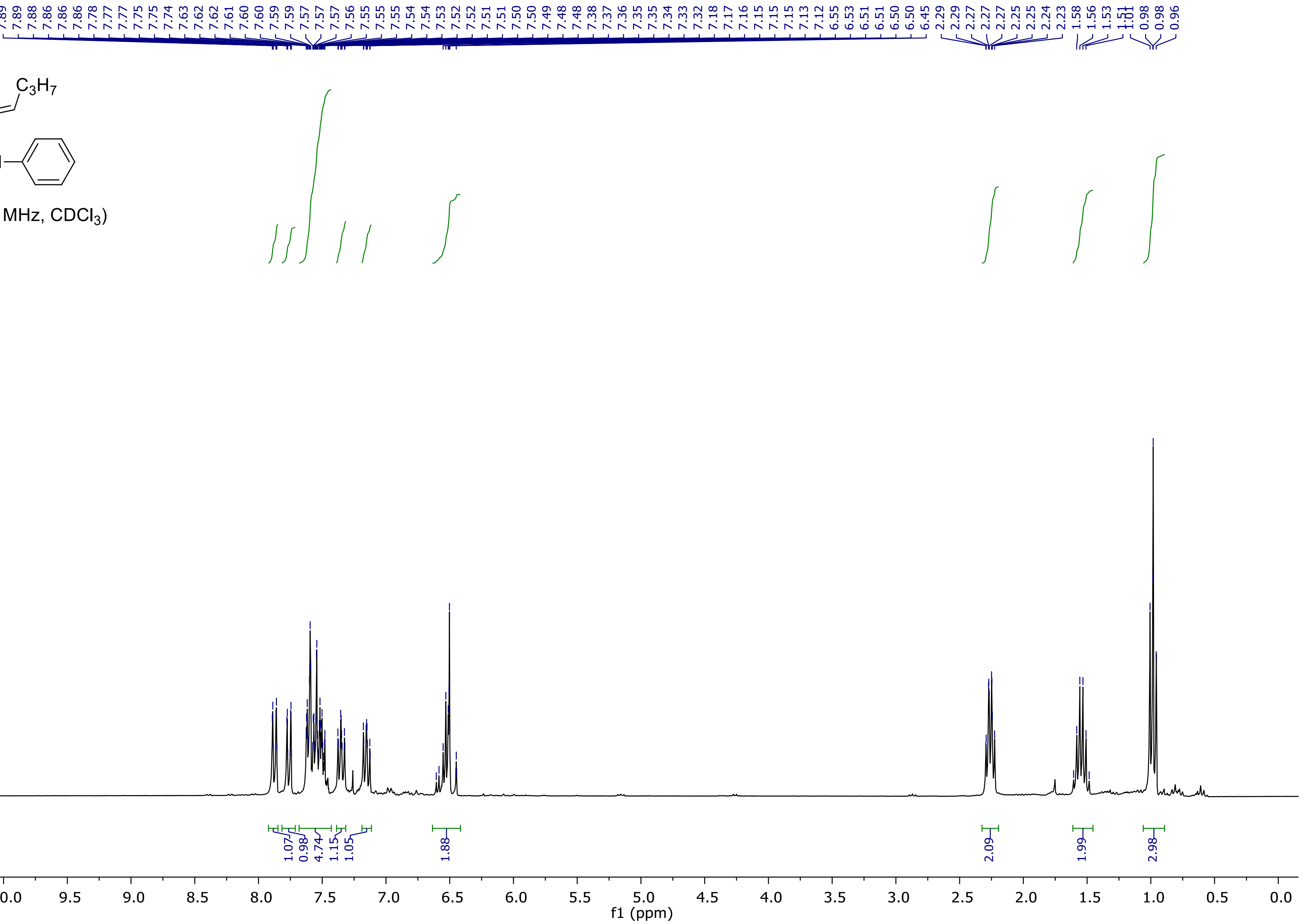

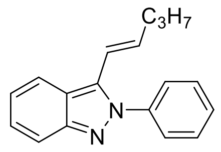

$^{13}C\{^1H\}$ NMR (75 MHz, $CDCl_3$)

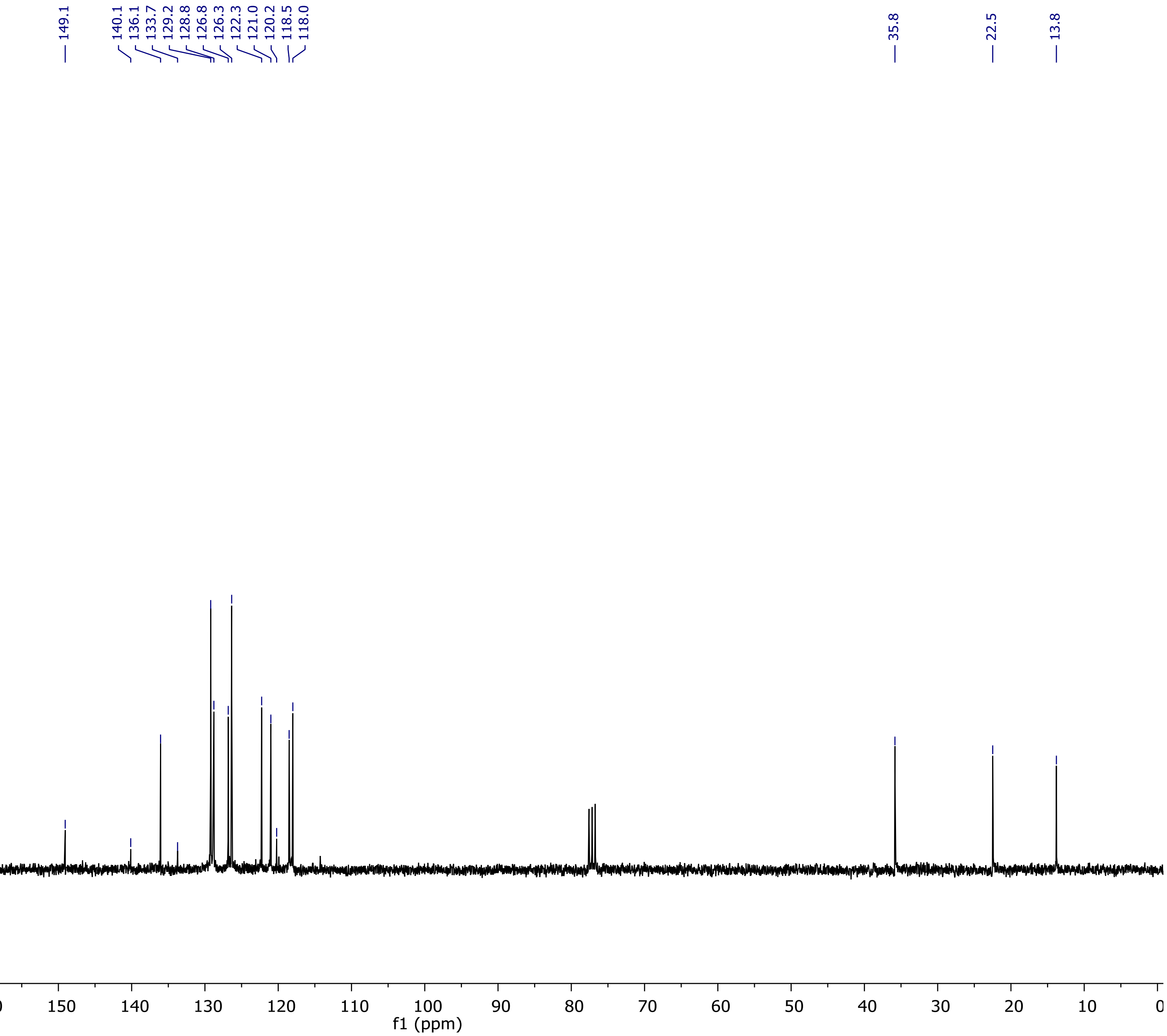

(*E*)-2-(4-Bromophenyl)-3-(pent-1-enyl)-2*H*-indazole (2b)

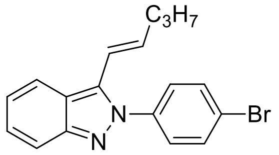

^{1}H-NMR (300 MHz, $CDCl_3$)

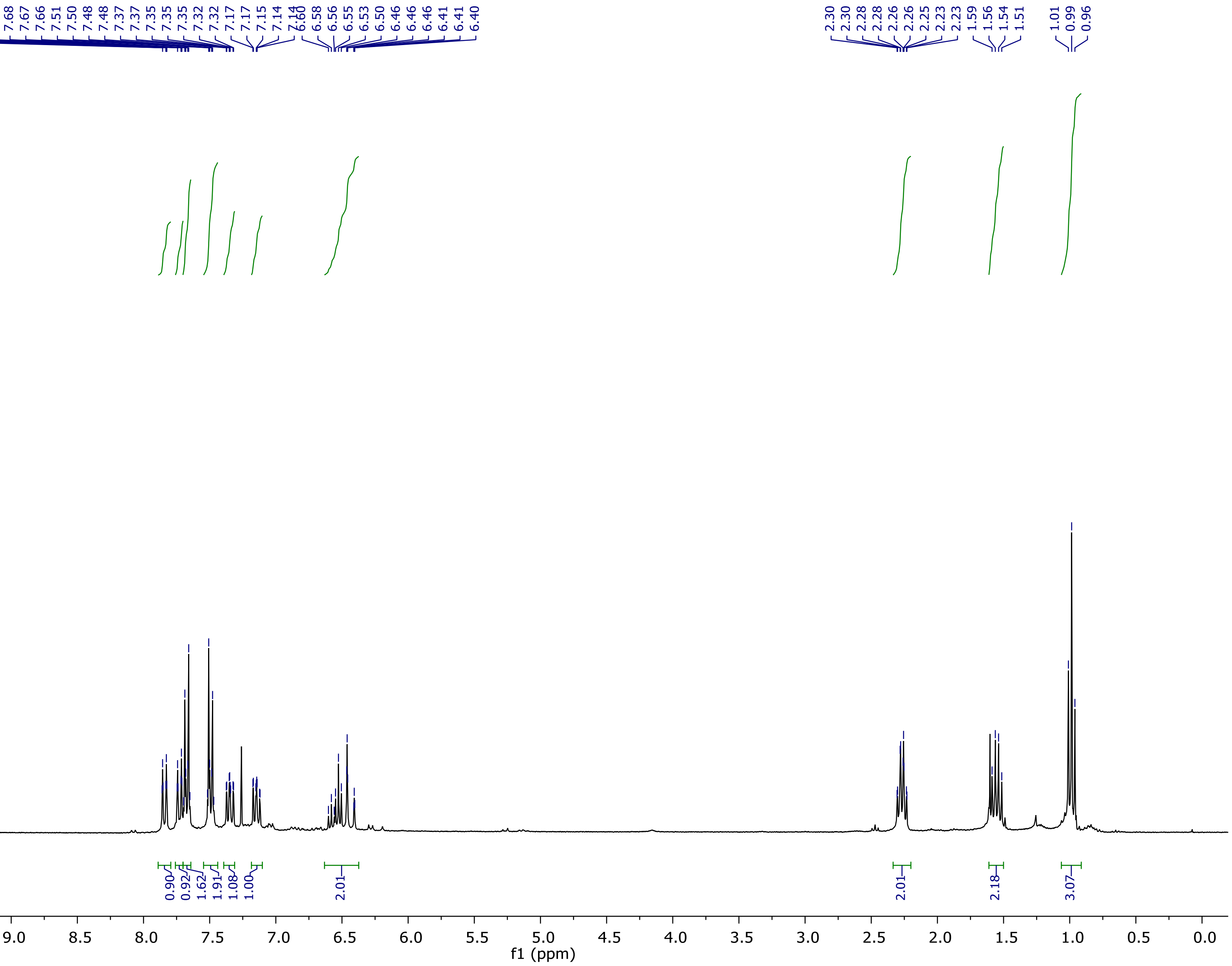

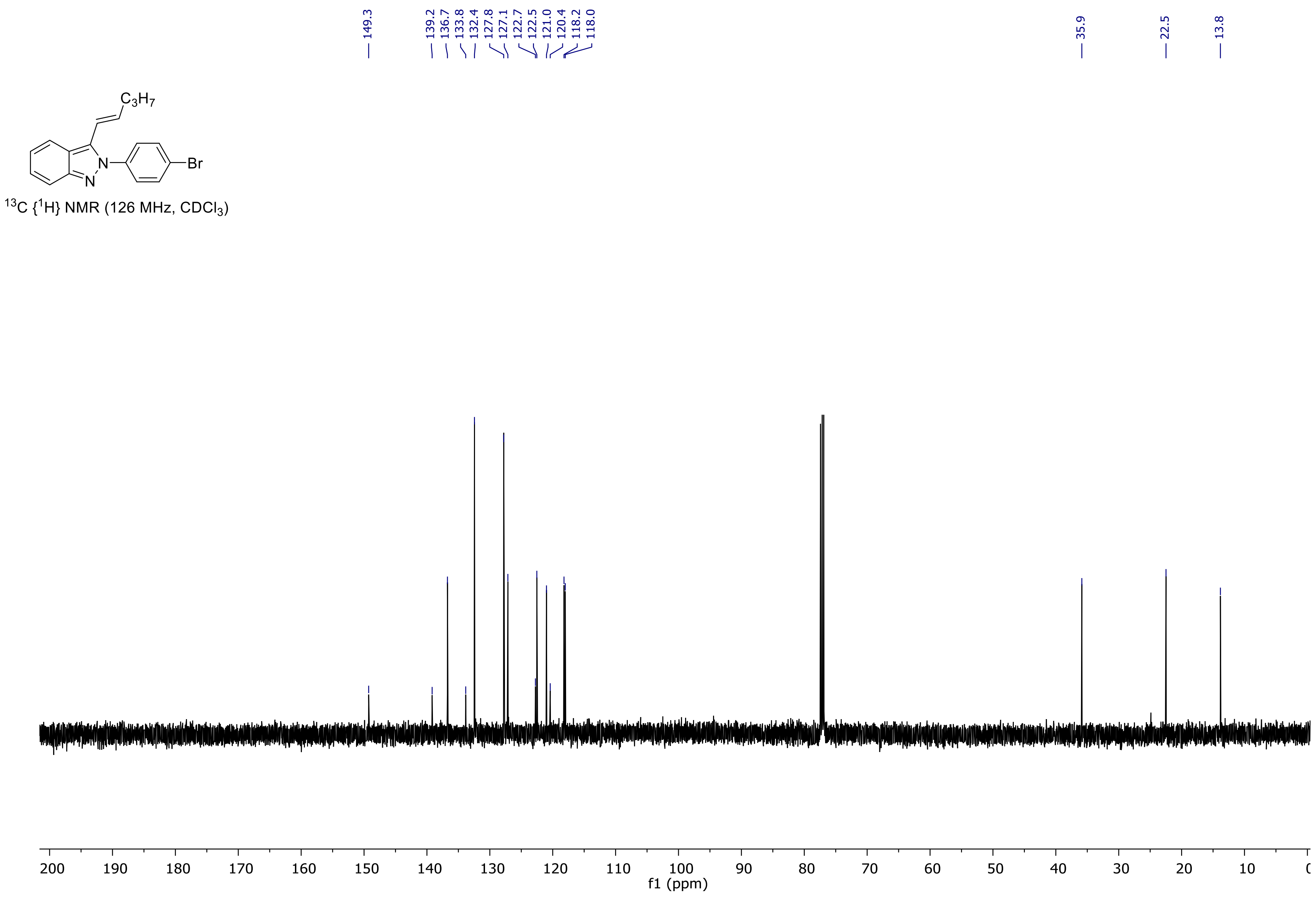

149.3
139.2
136.7
133.8
132.4
127.8
127.1
122.7
122.5
121.0
120.4
118.2
118.0
35.9
22.5
13.8
C3H7
N
N
Br
13C {1H} NMR (126 MHz, CDCl3)
200
190
180
170
160
150
140
130
120
110
100
90
80
70
60
50
40
30
20
10
f1 (ppm)

(*E*)-2-(3-Bromophenyl)-3-(pent-1-enyl)-2*H*-indazole (2c)

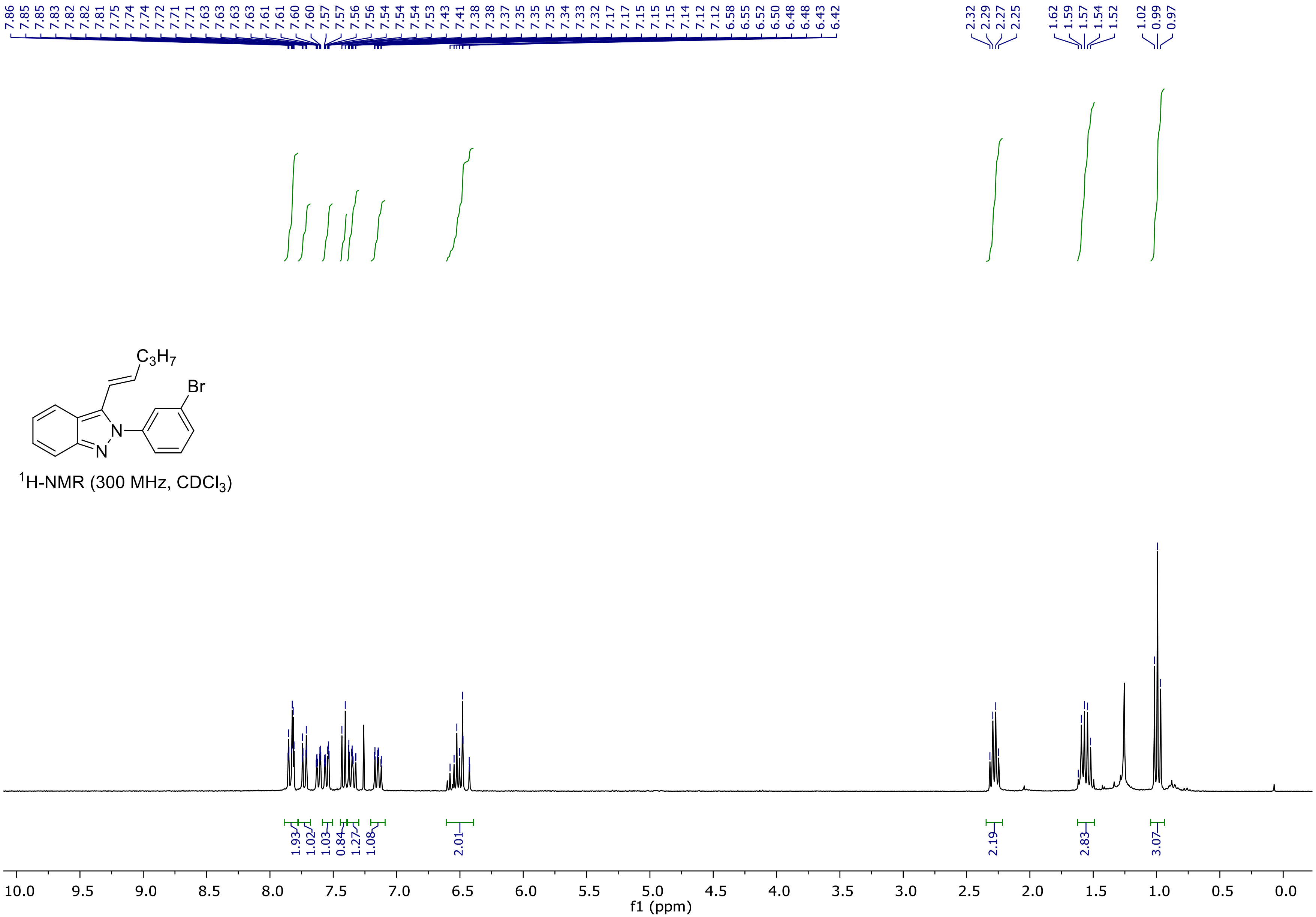

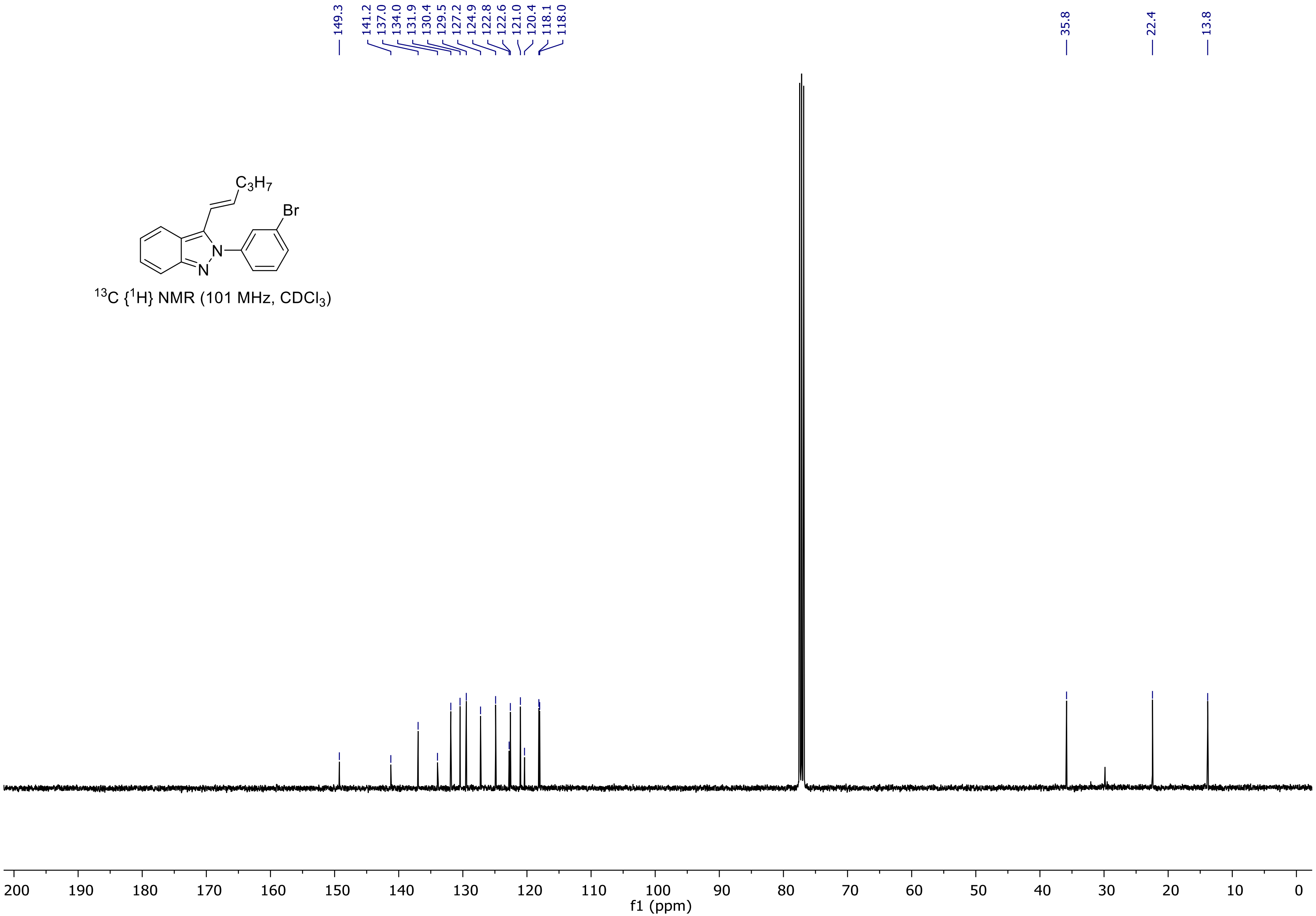

$^{13}C\ \{^{1}H\}$ NMR (101 MHz, $CDCl_3$)

(*E*)-2-(3-Fluorophenyl)-3-(pent-1-enyl)-2*H*-indazole (2d)

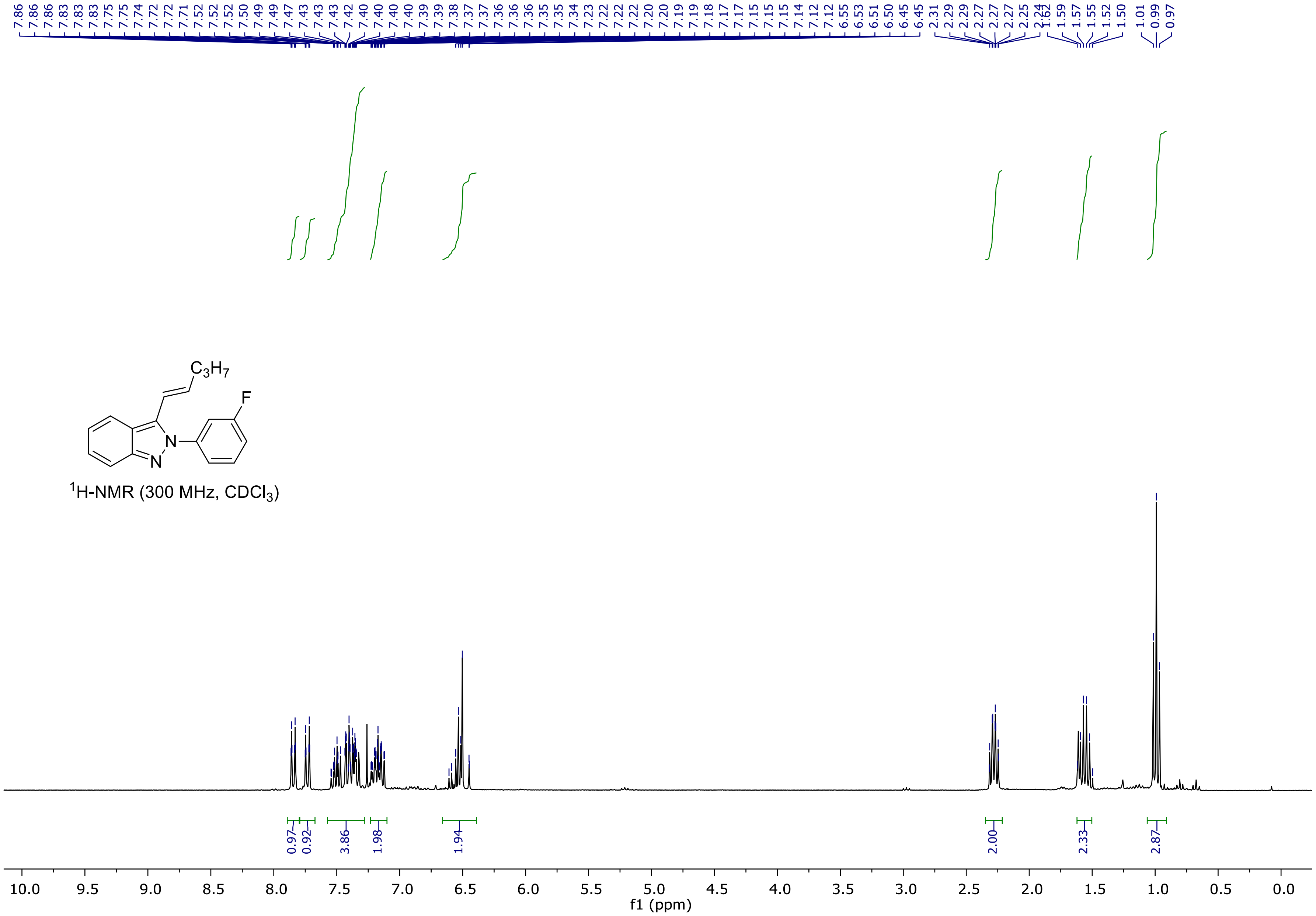

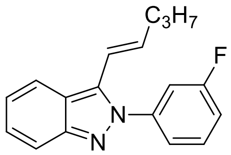

^{19}F-NMR (376 MHz, $CDCl_3$)

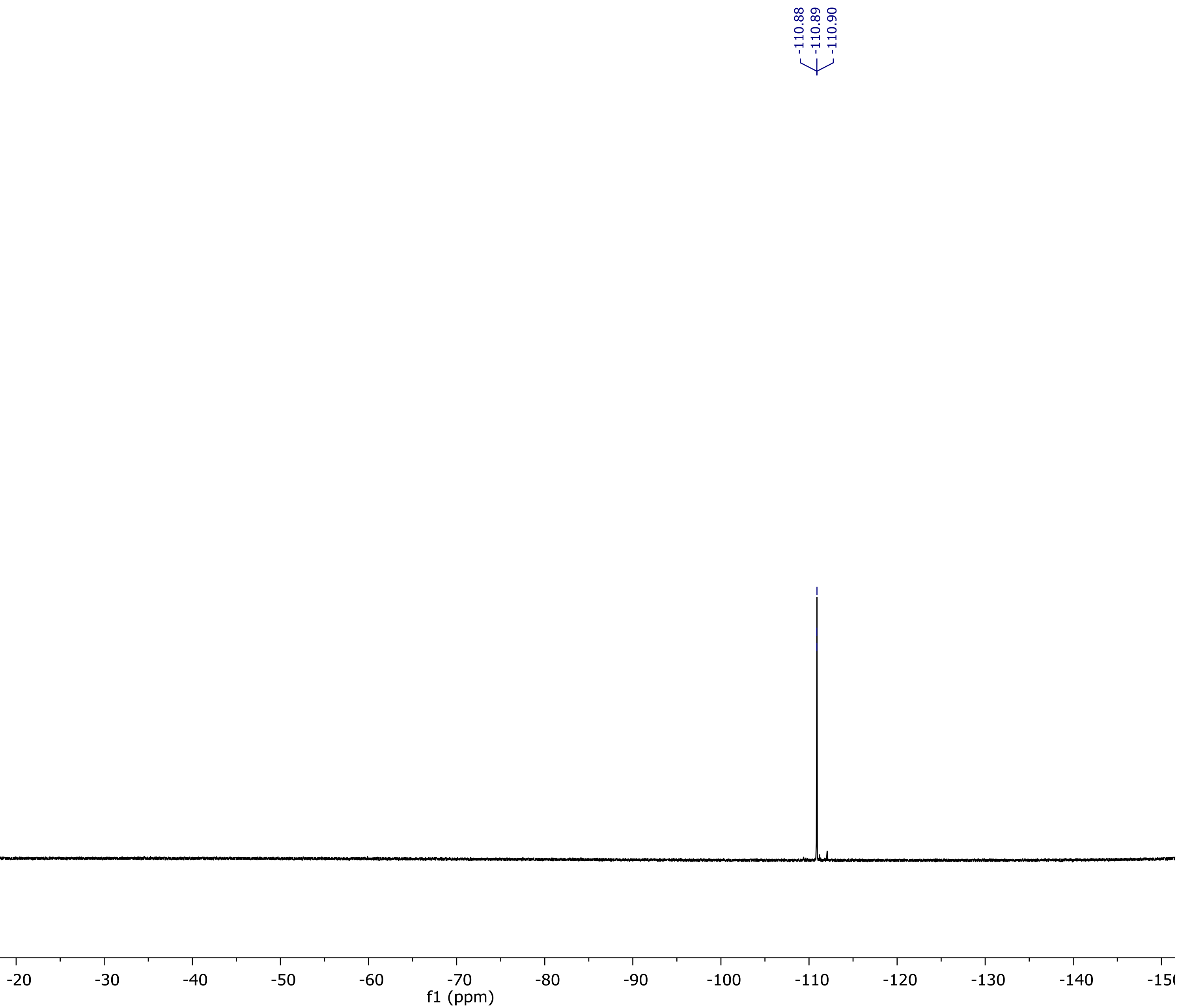

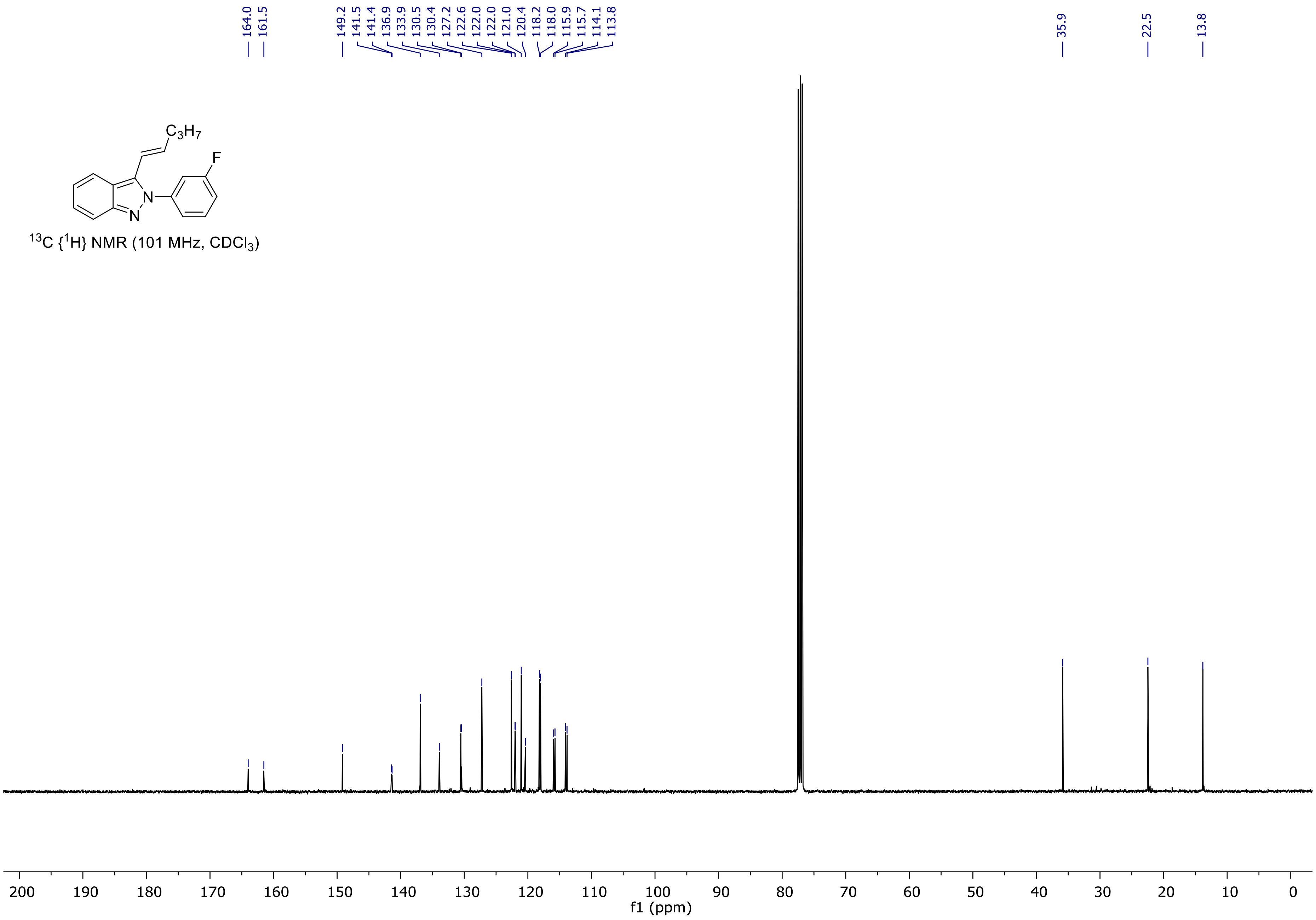

164.0
161.5
149.2
141.5
141.4
136.9
133.9
130.5
130.4
127.2
122.6
122.0
122.0
121.0
120.4
118.2
118.0
115.9
115.7
114.1
113.8
35.9
22.5
13.8
C3H7
F
N
N
13C {1H} NMR (101 MHz, CDCl3)
200 190 180 170 160 150 140 130 120 110 100 90 80 70 60 50 40 30 20 10 0
f1 (ppm)

(*E*)-2-(2-Fluorophenyl)-3-(pent-1-enyl)-2*H*-indazole (2e)

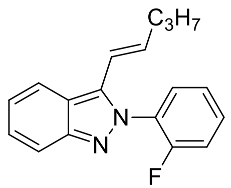

^{1}H-NMR (300 MHz, $CDCl_3$)

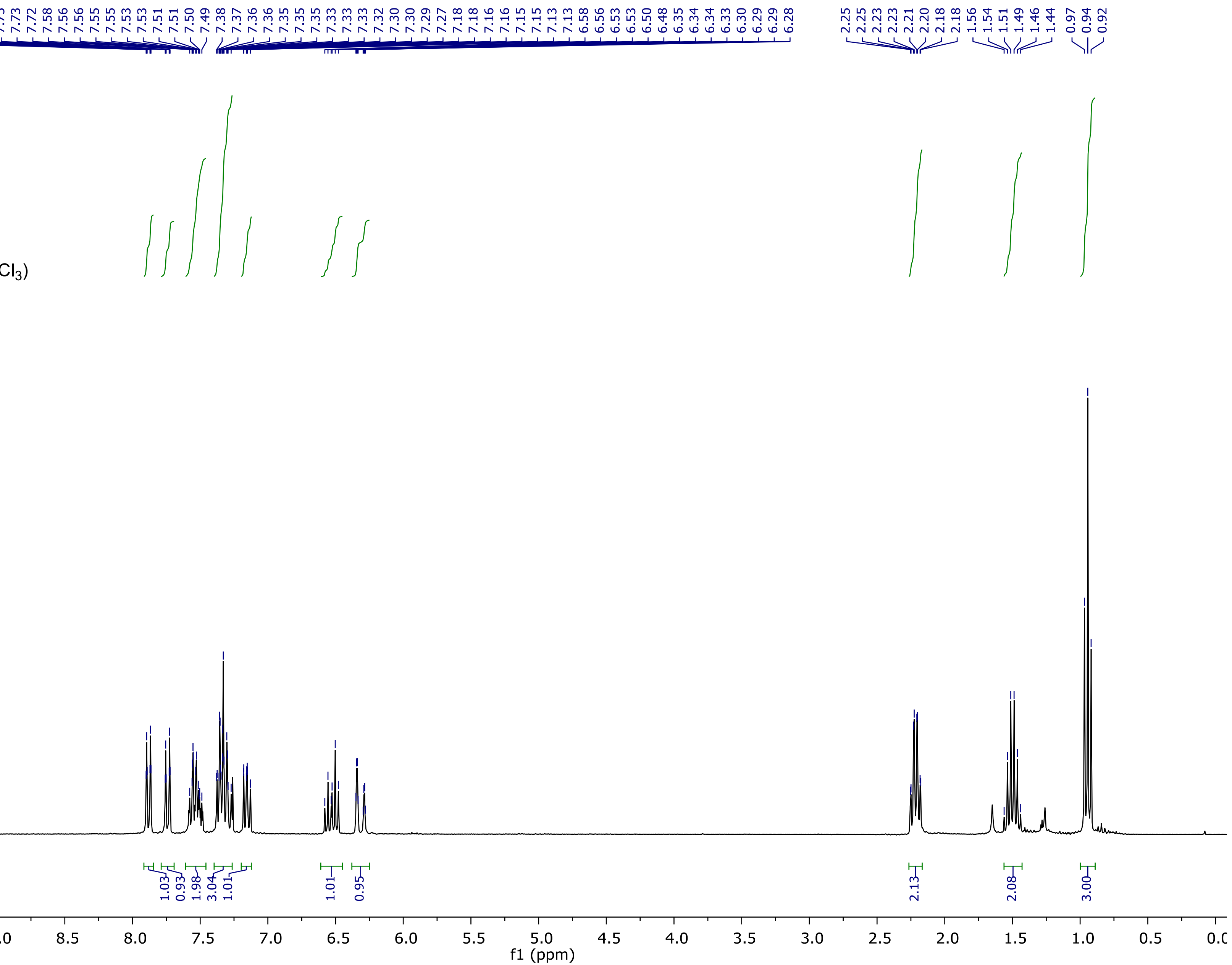

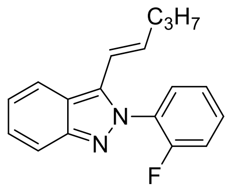

^{19}F-NMR (376 MHz, $CDCl_3$)

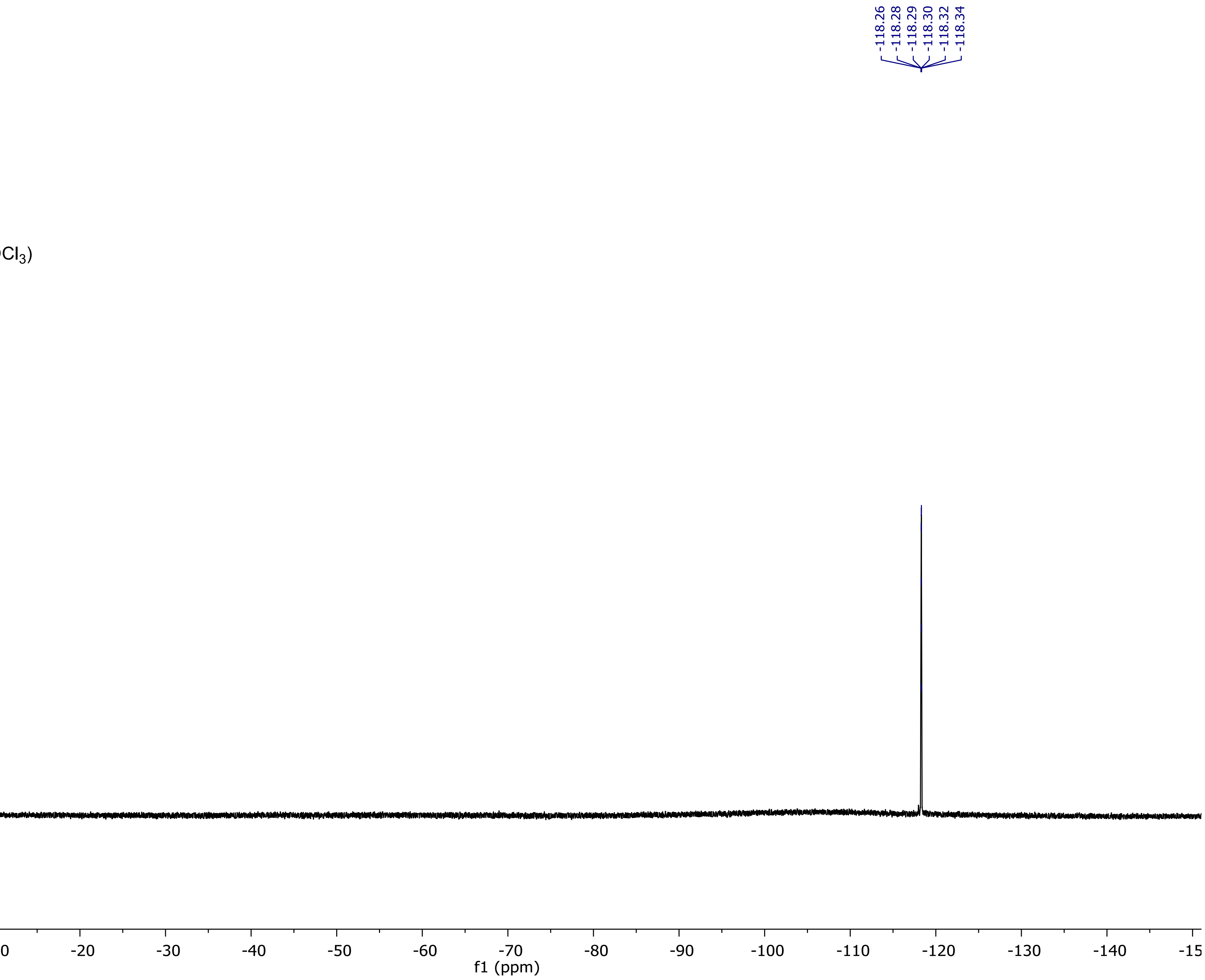

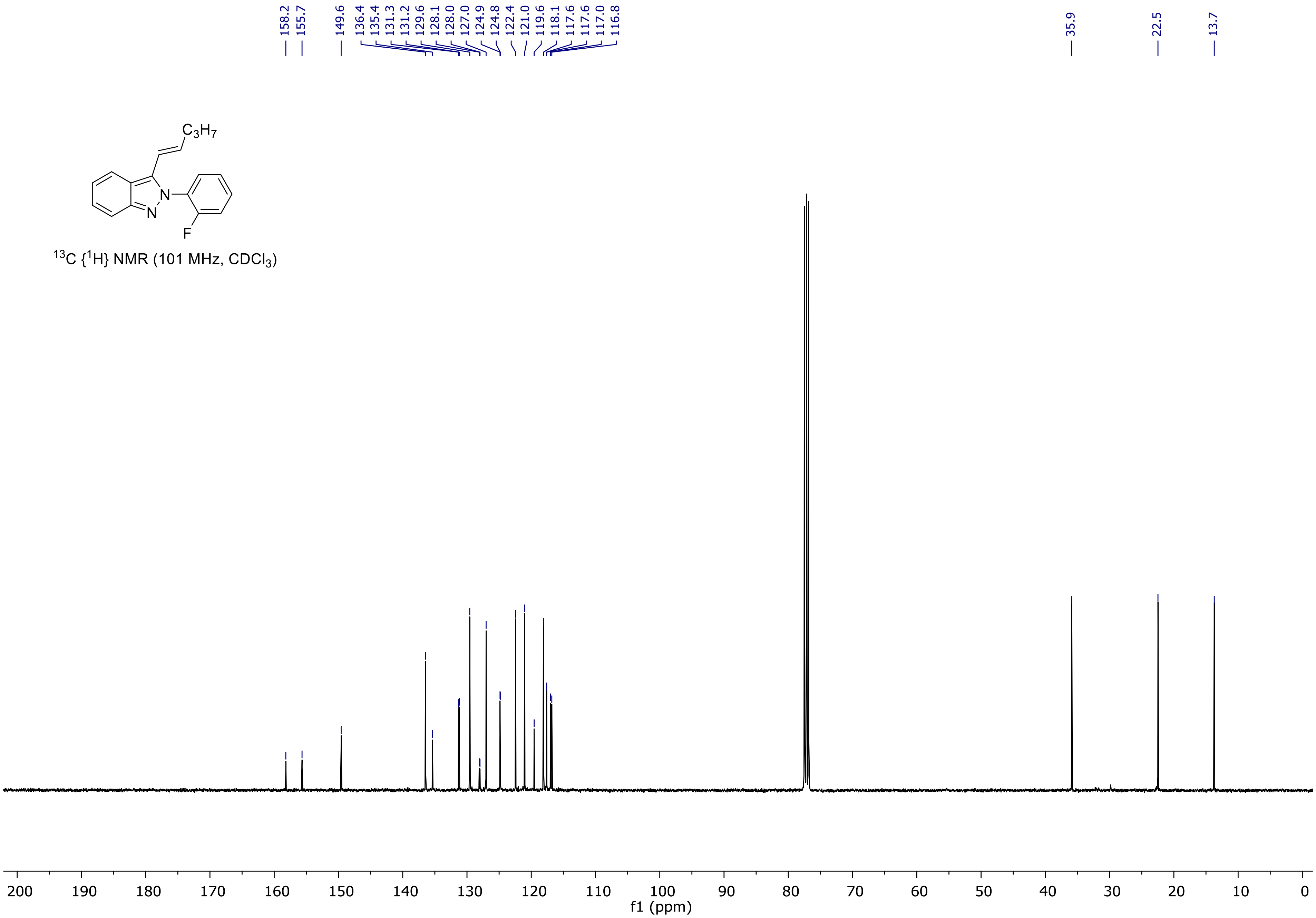
158.2
155.7
149.6
136.4
135.4
131.3
131.2
129.6
128.1
128.0
127.0
124.9
124.8
122.4
121.0
119.6
118.1
117.6
117.6
117.0
116.8
35.9
22.5
13.7
C_3H_7
N
N
F
^{13}C {^1H} NMR (101 MHz, $CDCl_3$)
200 190 180 170 160 150 140 130 120 110 100 90 80 70 60 50 40 30 20 10 0
f1 (ppm)

(*E*)-4-[3-(Pent-1-enyl)-2*H*-indazol-2-yl]benzonitrile (2f)

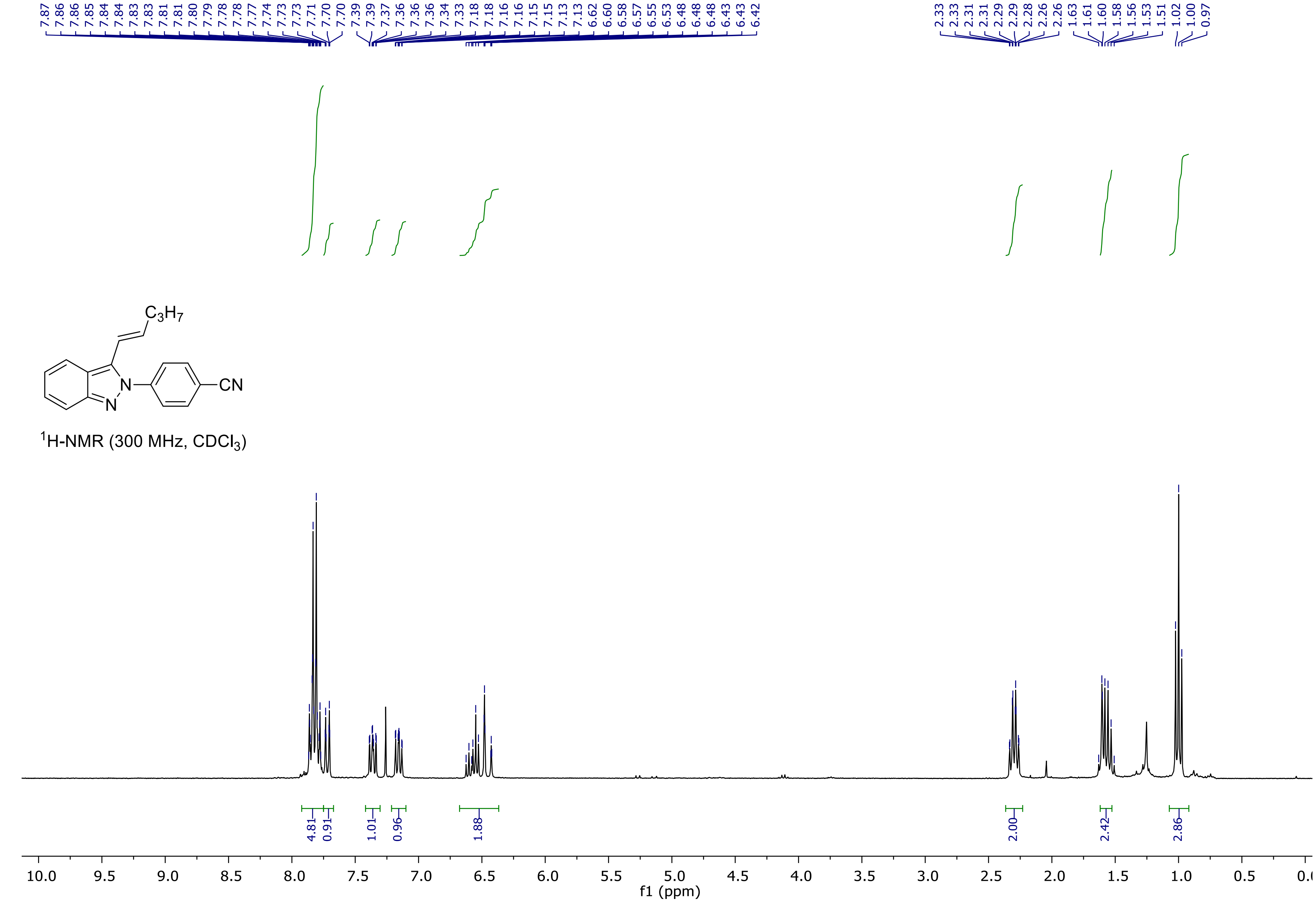

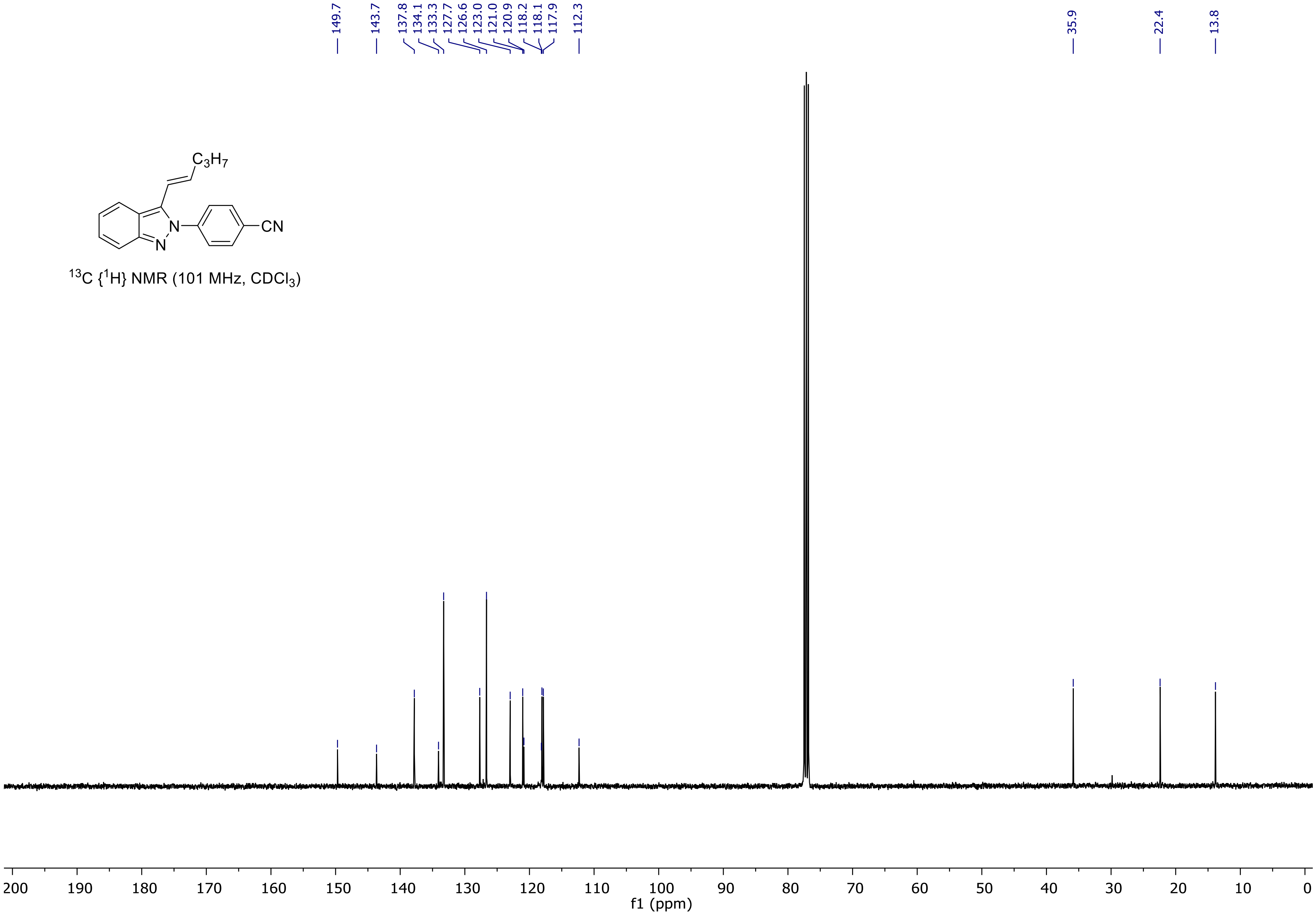
149.7
143.7
137.8
134.1
133.3
127.7
126.6
123.0
121.0
120.9
118.2
118.1
117.9
112.3
35.9
22.4
13.8
C3H7
N
N
CN
13C {1H} NMR (101 MHz, CDCl3)
200 190 180 170 160 150 140 130 120 110 100 90 80 70 60 50 40 30 20 10 0
f1 (ppm)

(*E*)-2-(3-Nitrophenyl)-3-(pent-1-en-1-yl)-2*H*-indazole (2g)

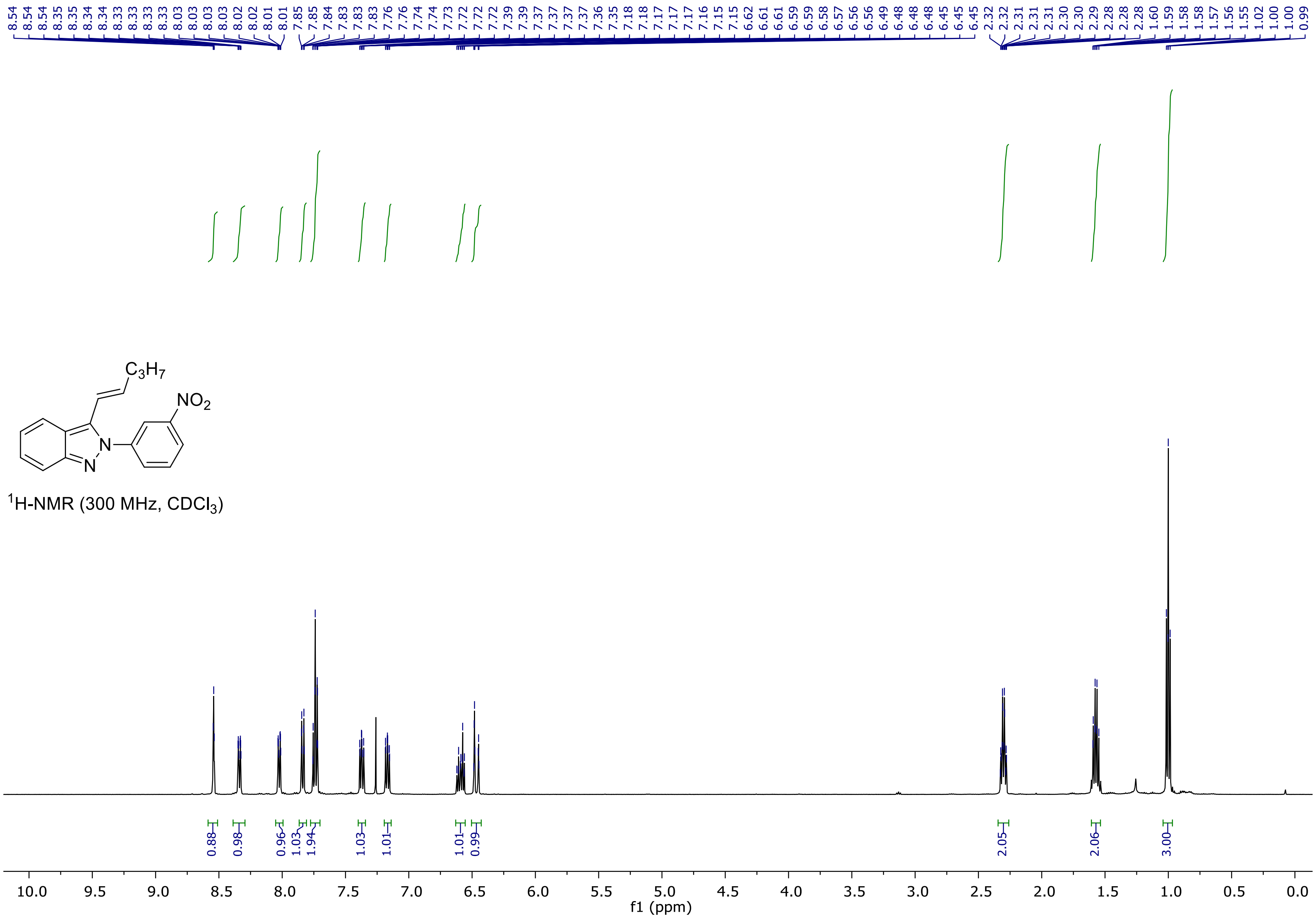

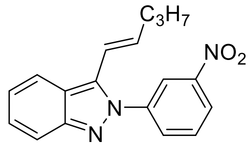

^{13}C {^1H} NMR (126 MHz, $CDCl_3$)

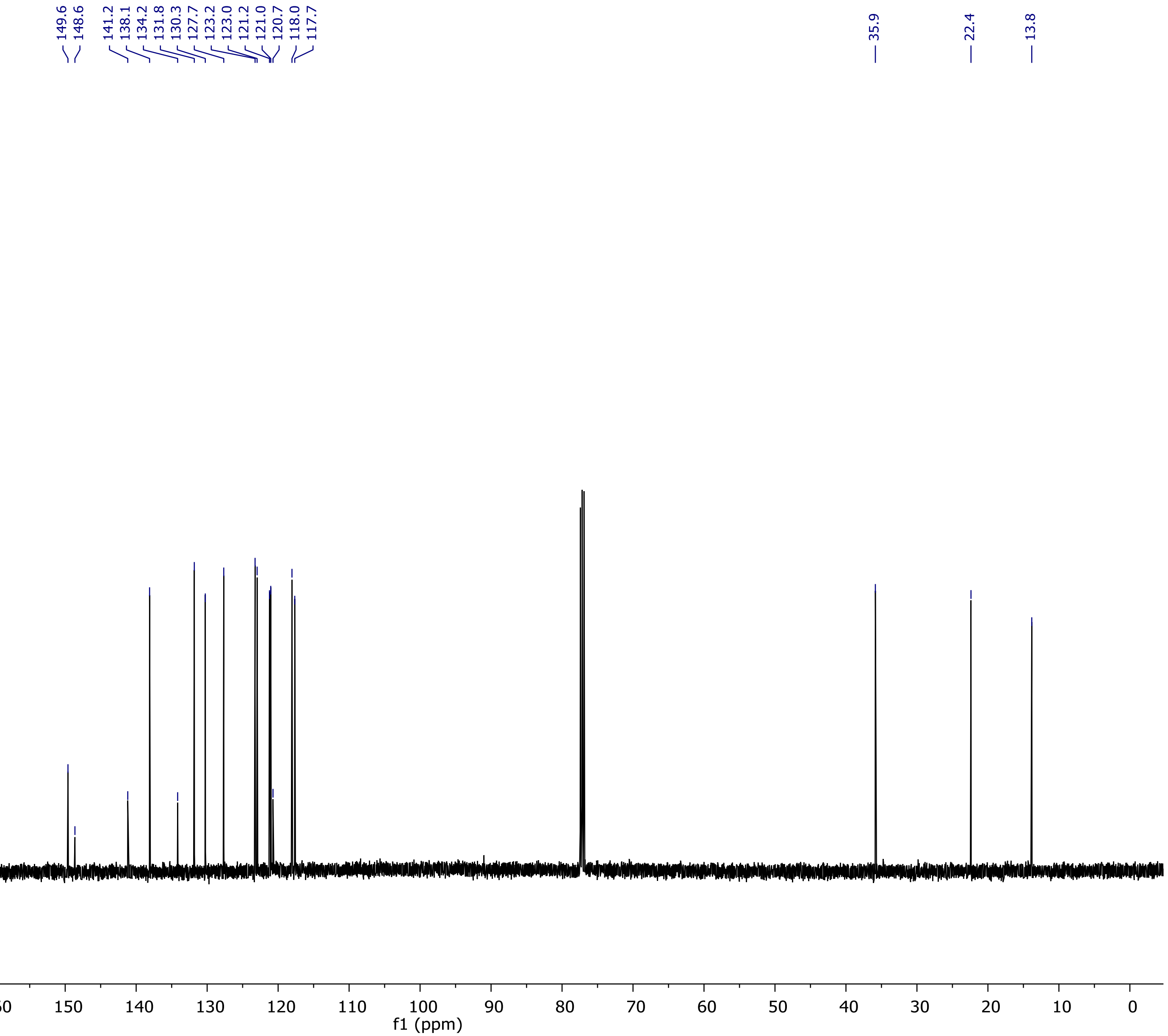

(*E*)-1-[4-(3-(Pent-1-enyl)-2*H*-indazol-2-yl)phenyl]ethan-1-one (2h)

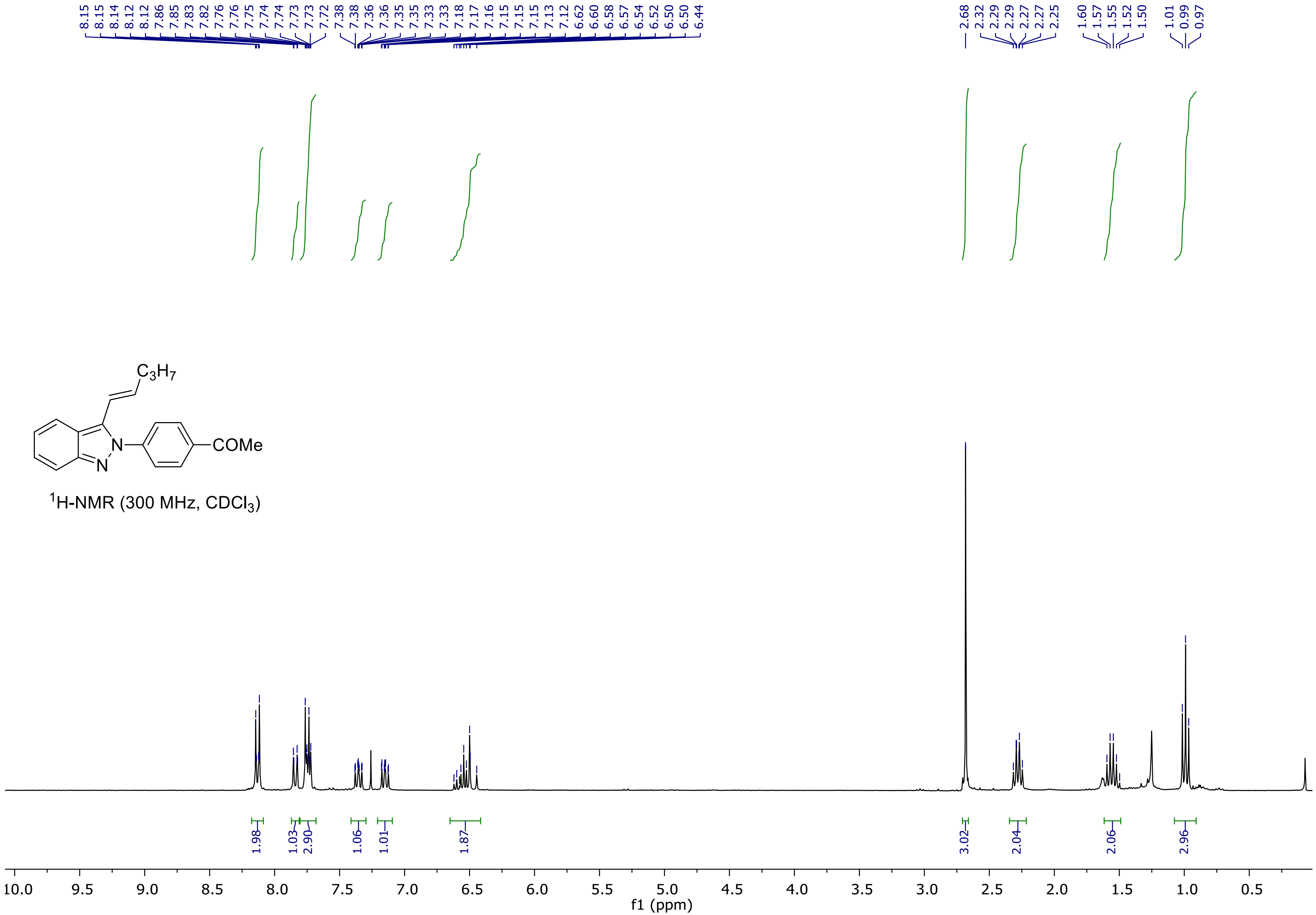

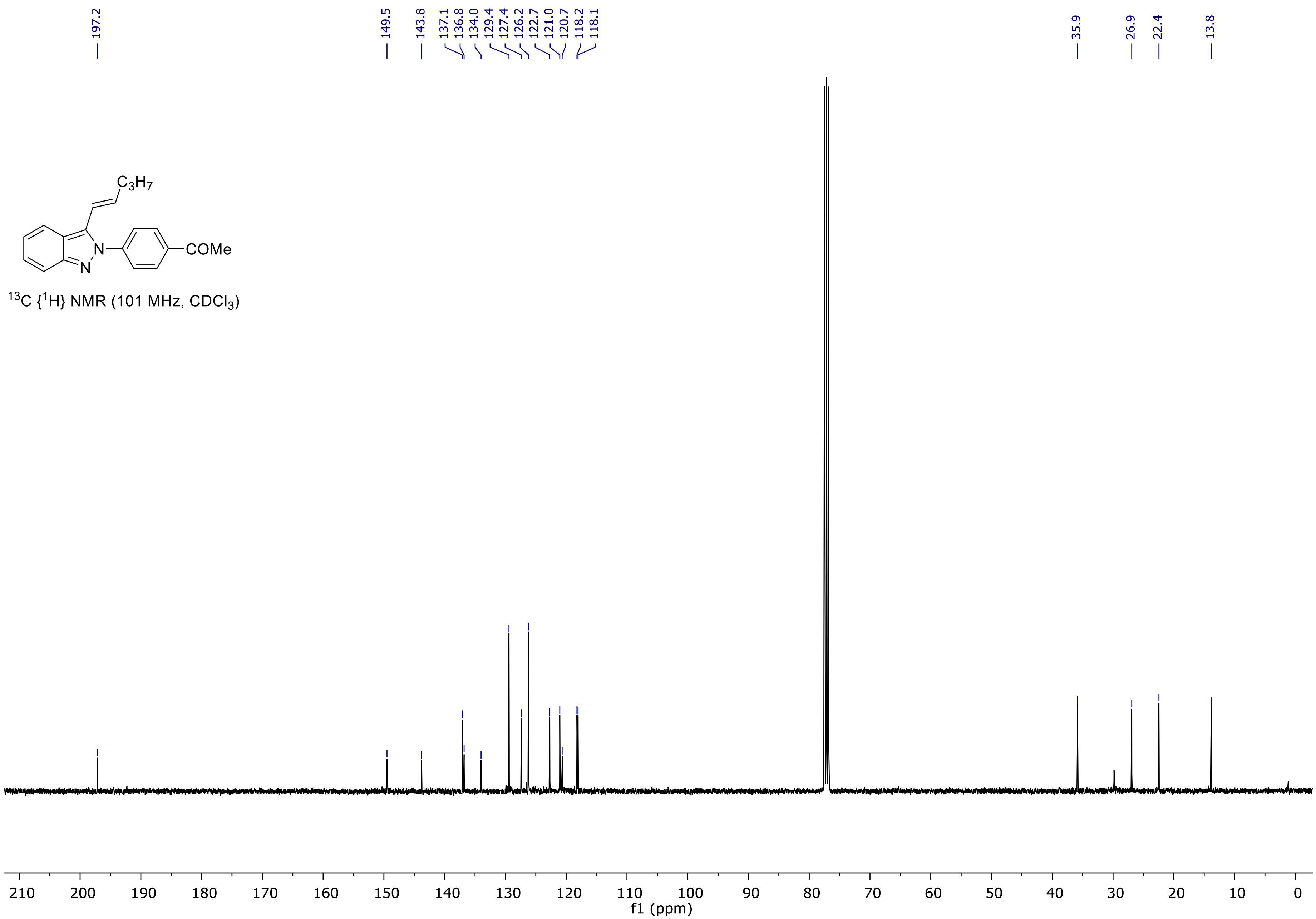

197.2
149.5
143.8
137.1
136.8
134.0
129.4
127.4
126.2
122.7
121.0
120.7
118.2
118.1
35.9
26.9
22.4
13.8
C3H7
N
N
COMe
13C {1H} NMR (101 MHz, CDCl3)
210 200 190 180 170 160 150 140 130 120 110 100 90 80 70 60 50 40 30 20 10 0
f1 (ppm)

(*E*)-Dimethyl 5-[3-(pent-1-en-1-yl)-2*H*-indazol-2-yl]isophthalate (2i)

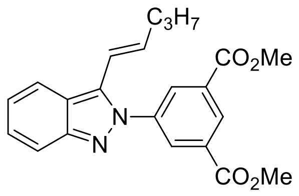

^{1}H-NMR (500 MHz, $CDCl_3$)

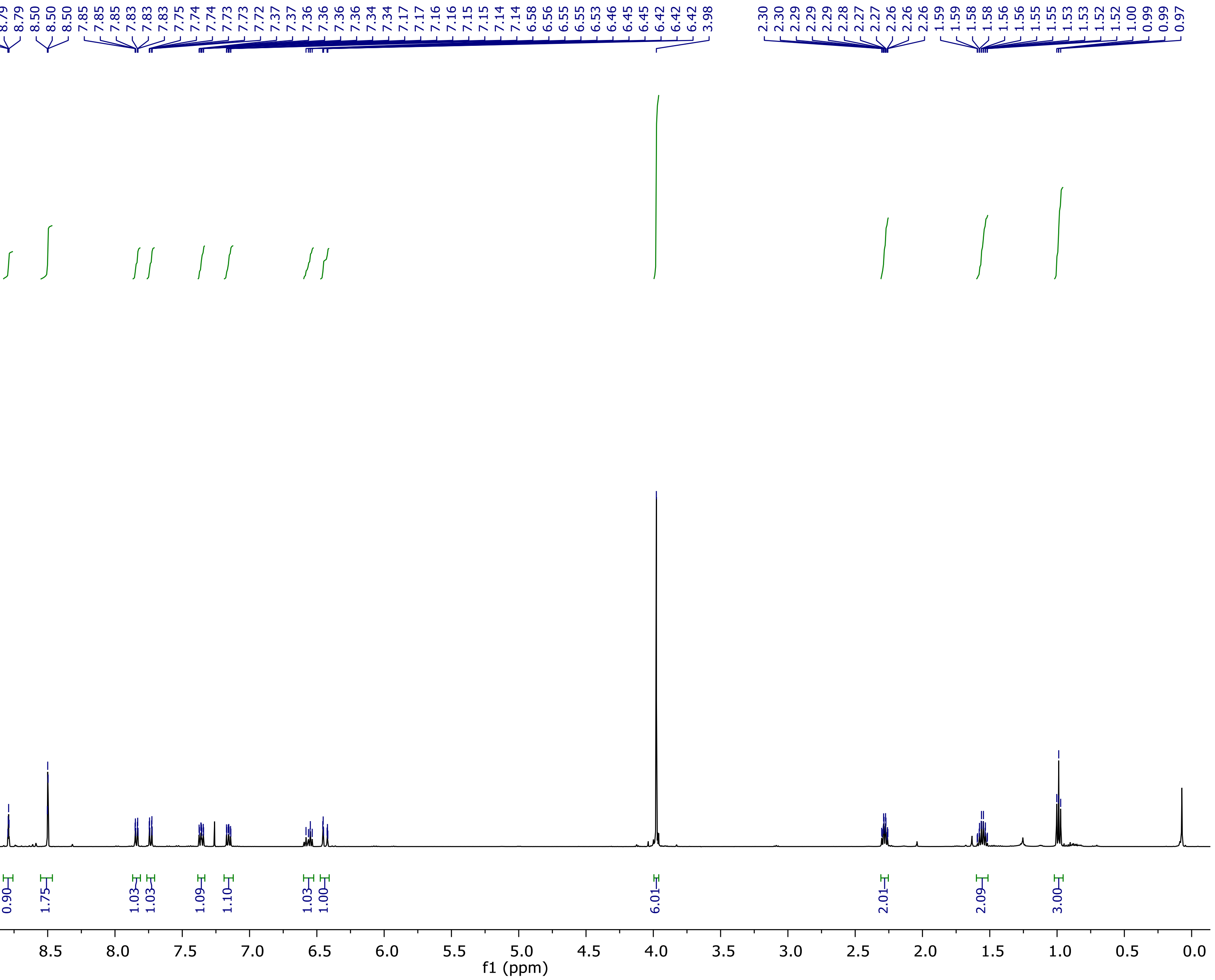

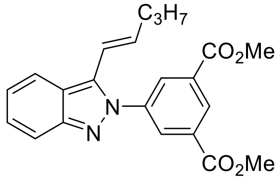

^{13}C {^{1}H} NMR (126 MHz, $CDCl_3$)

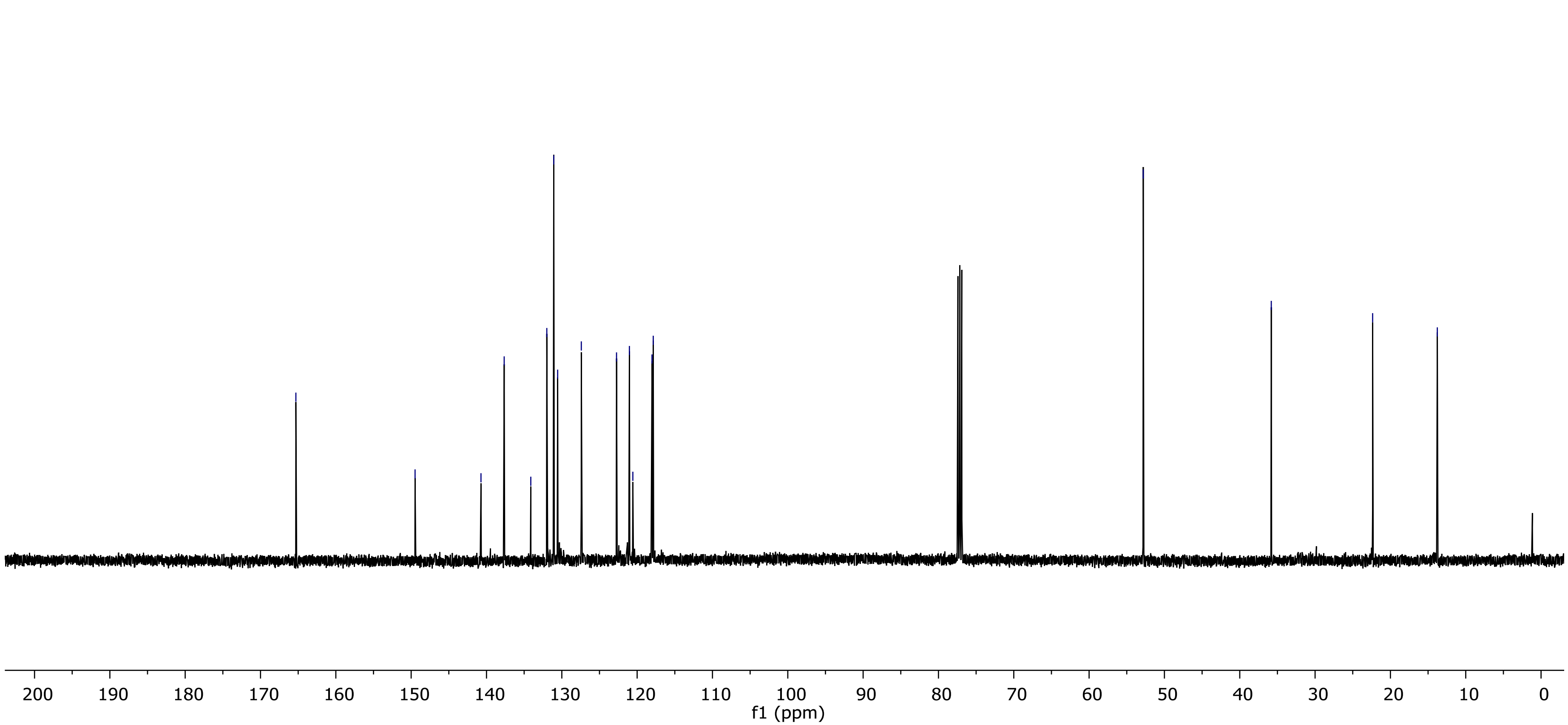

(*E*)-2-(2-Bromo-4-methylphenyl)-3-(pent-1-enyl)-2*H*-indazole (2j)

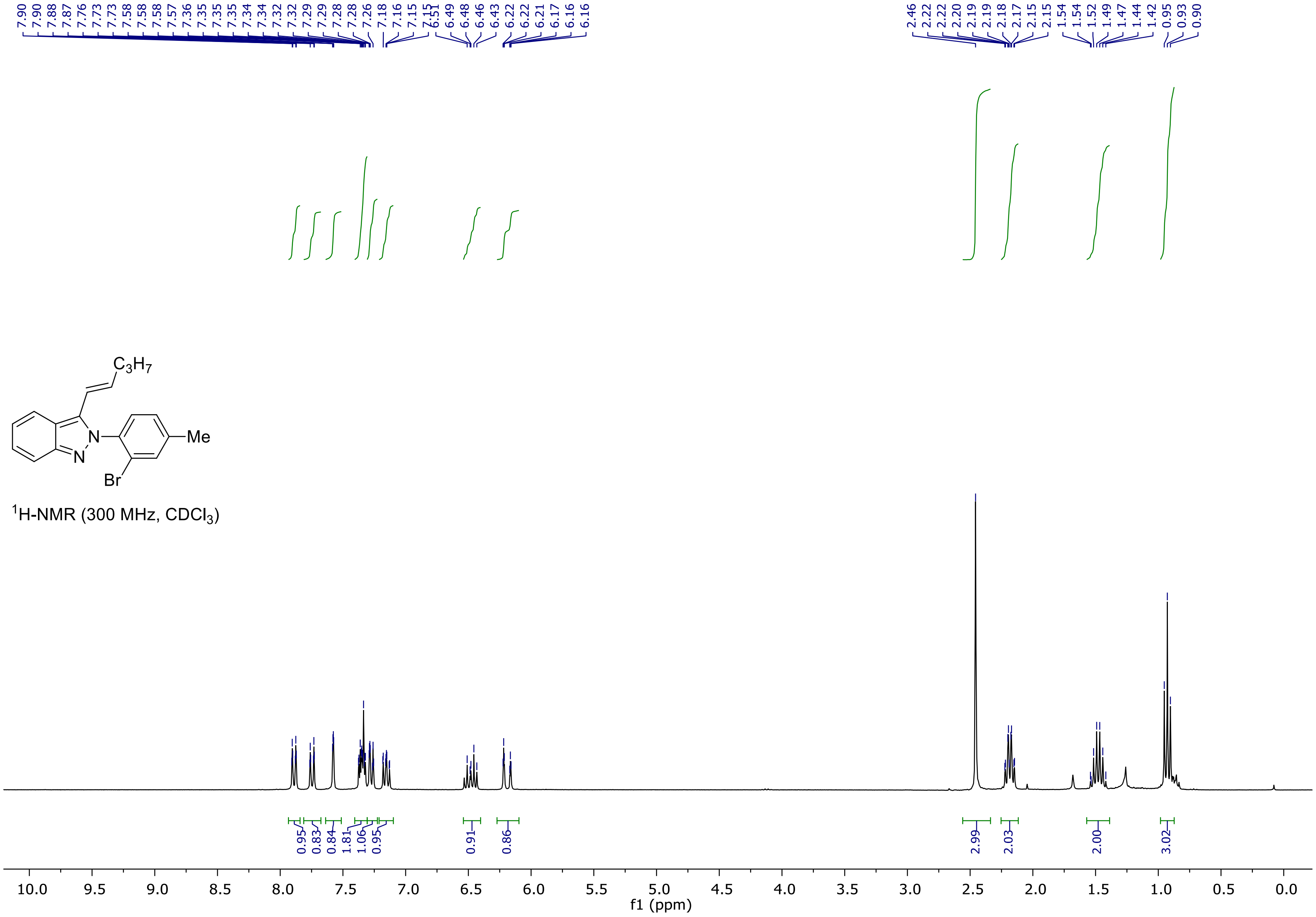

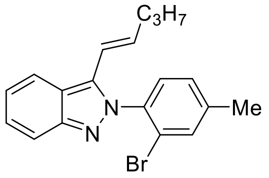

$^{13}C\ \{^{1}H\}$ NMR (126 MHz, $CDCl_3$)

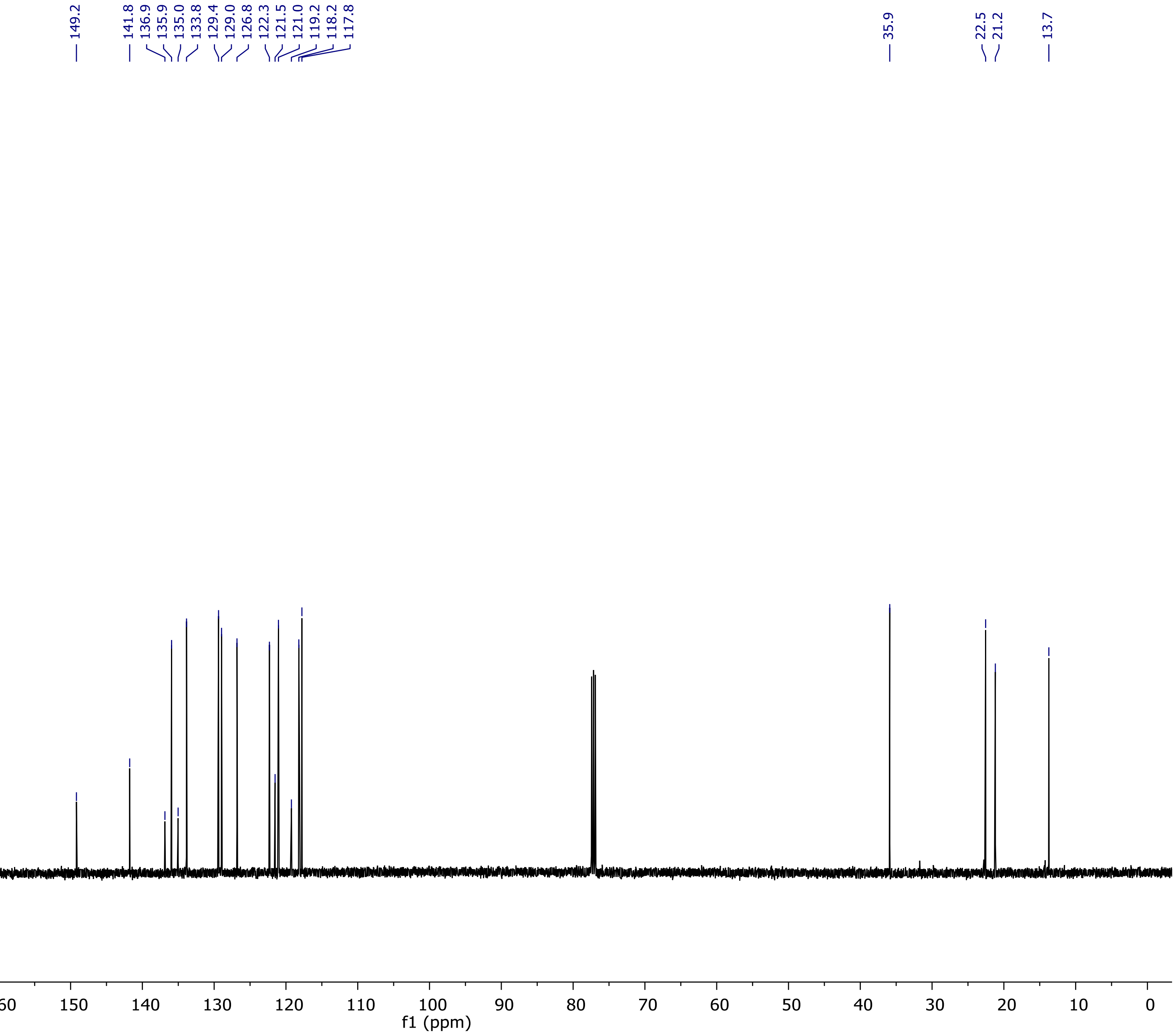

(*E*)-3-(Pent-1-en-1-yl)-2-(perfluorophenyl)-2*H*-indazole (2k)

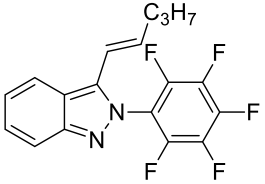

^{1}H-NMR (300 MHz, $CDCl_3$)

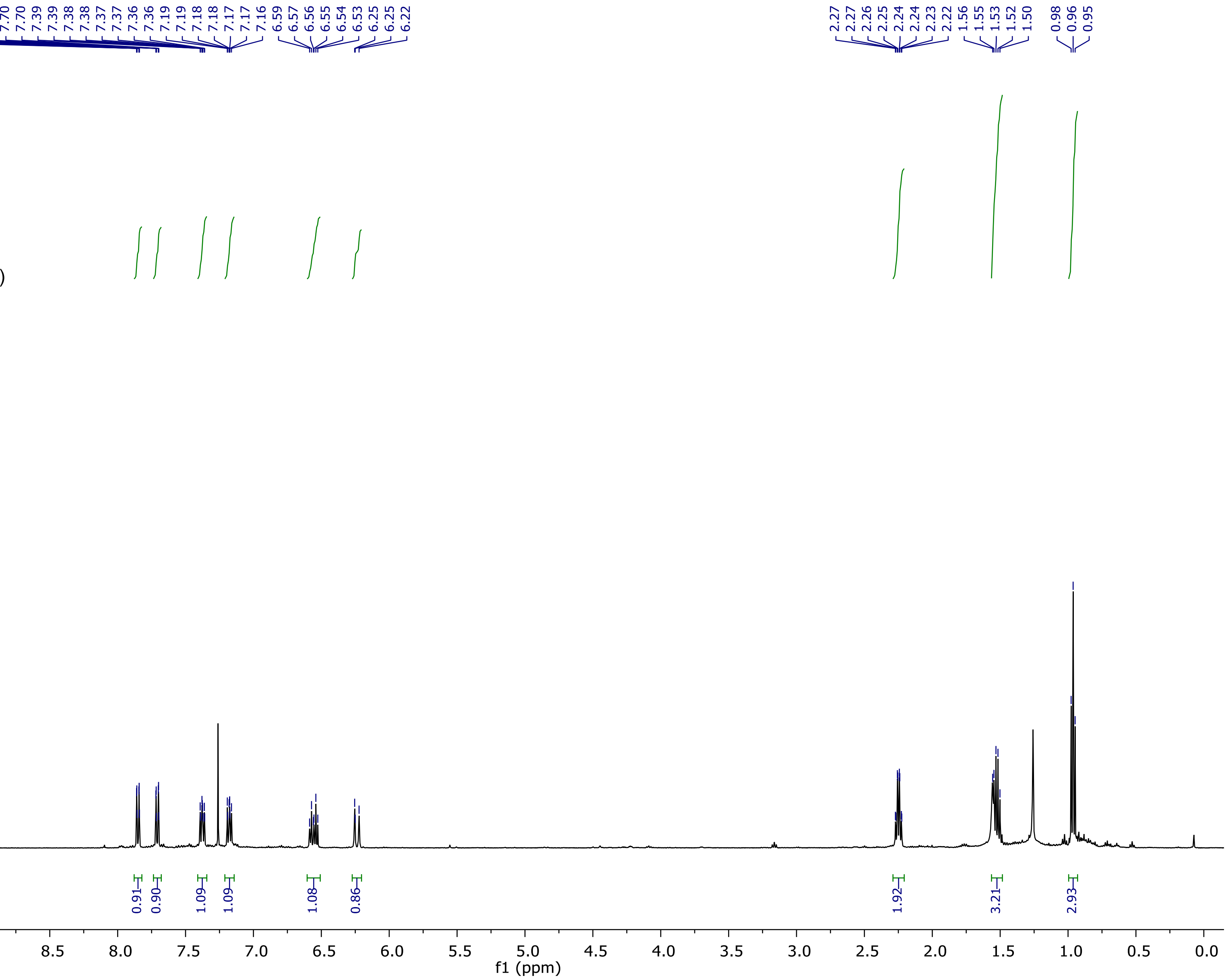

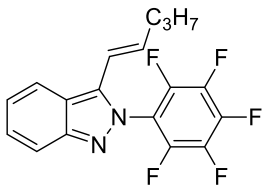

^{19}F-NMR (282 MHz, $CDCl_3$)

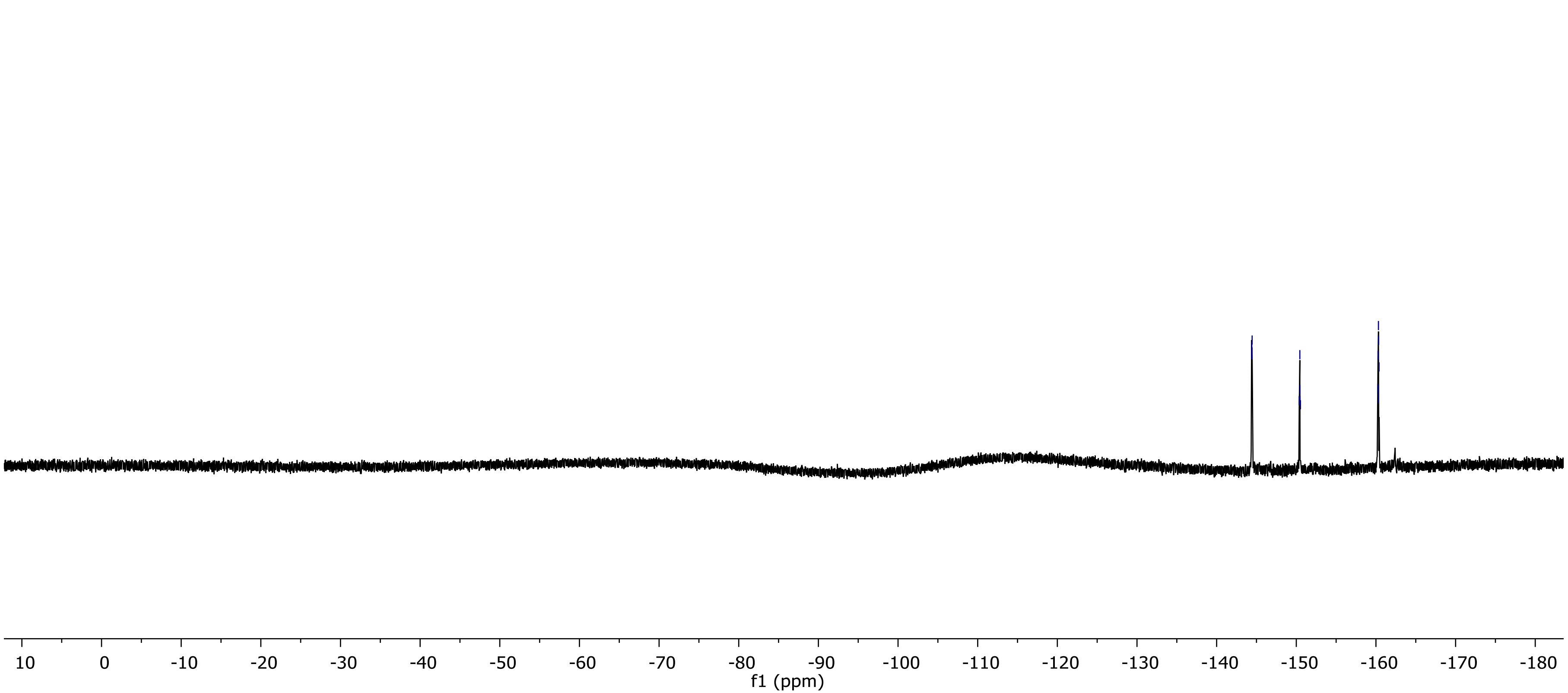

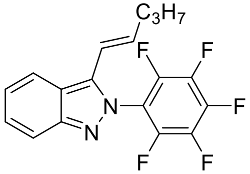

$^{13}C\{^1H\}$ NMR (126 MHz, $CDCl_3$)

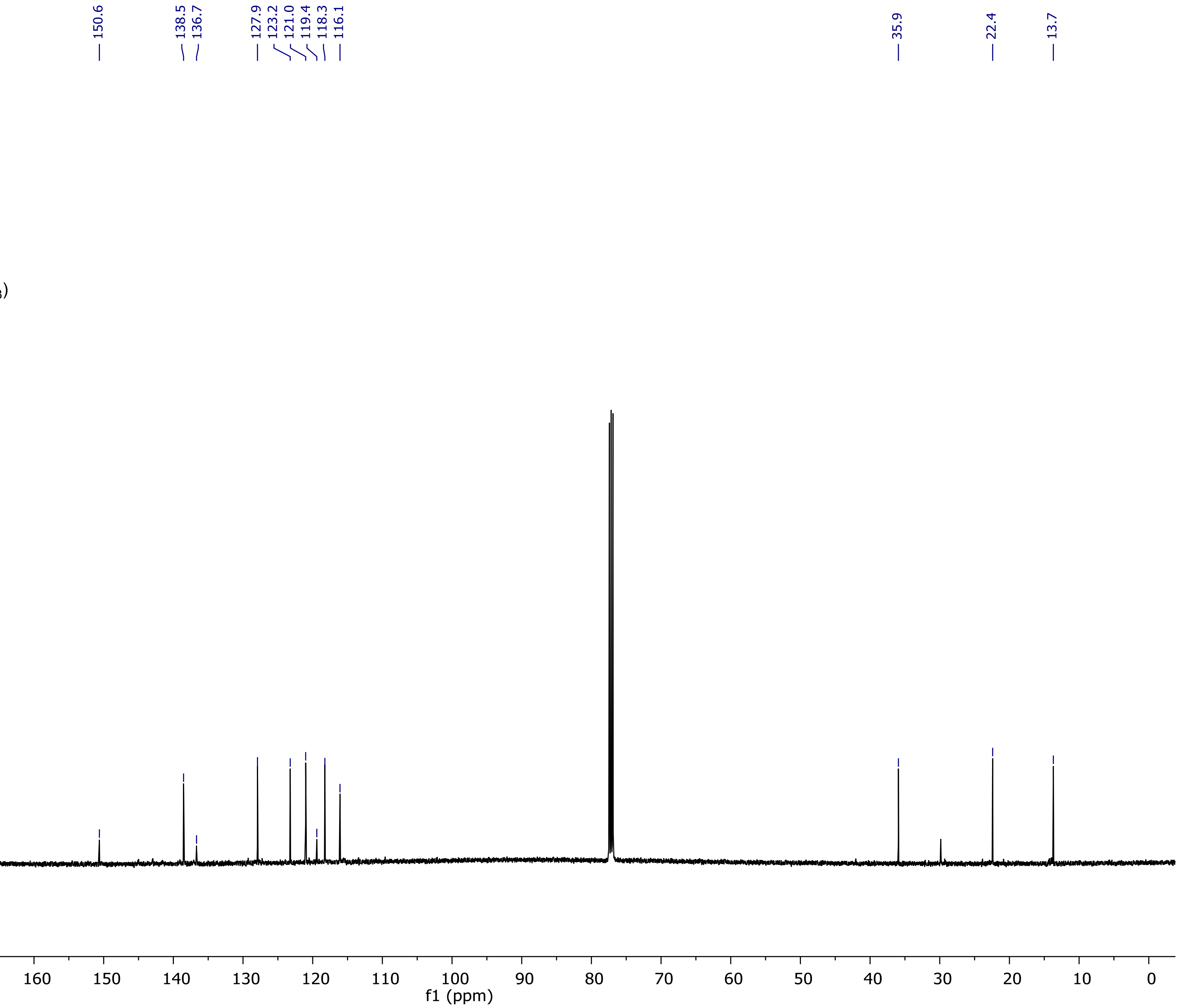

(*E*)-3-(Pent-1-enyl)-2-(*p*-tolyl)-2*H*-indazole (2l)

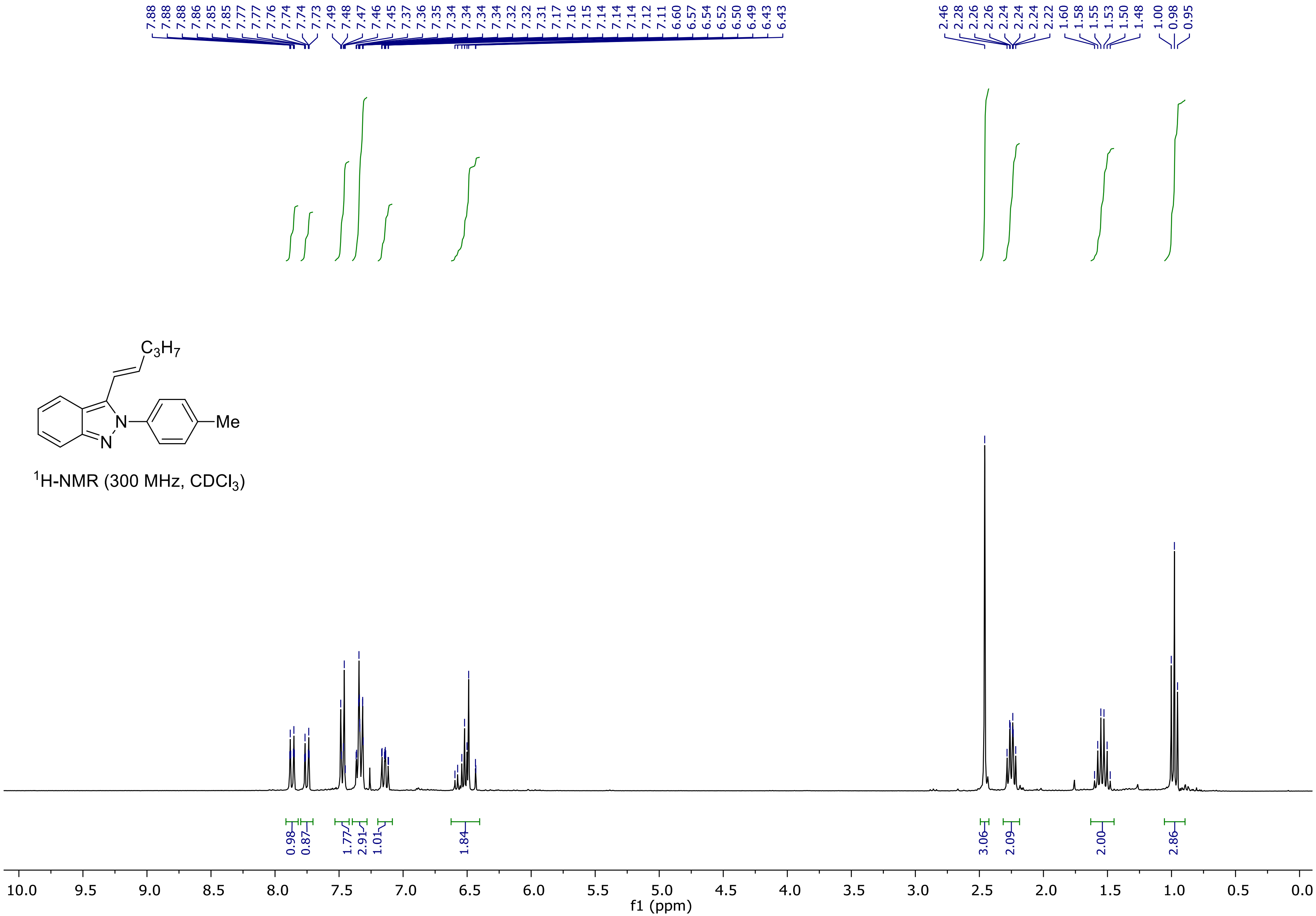

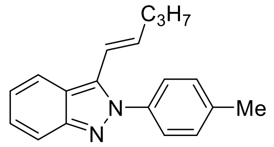

$^{13}C\ \{^{1}H\}$ NMR (101 MHz, $CDCl_3$)

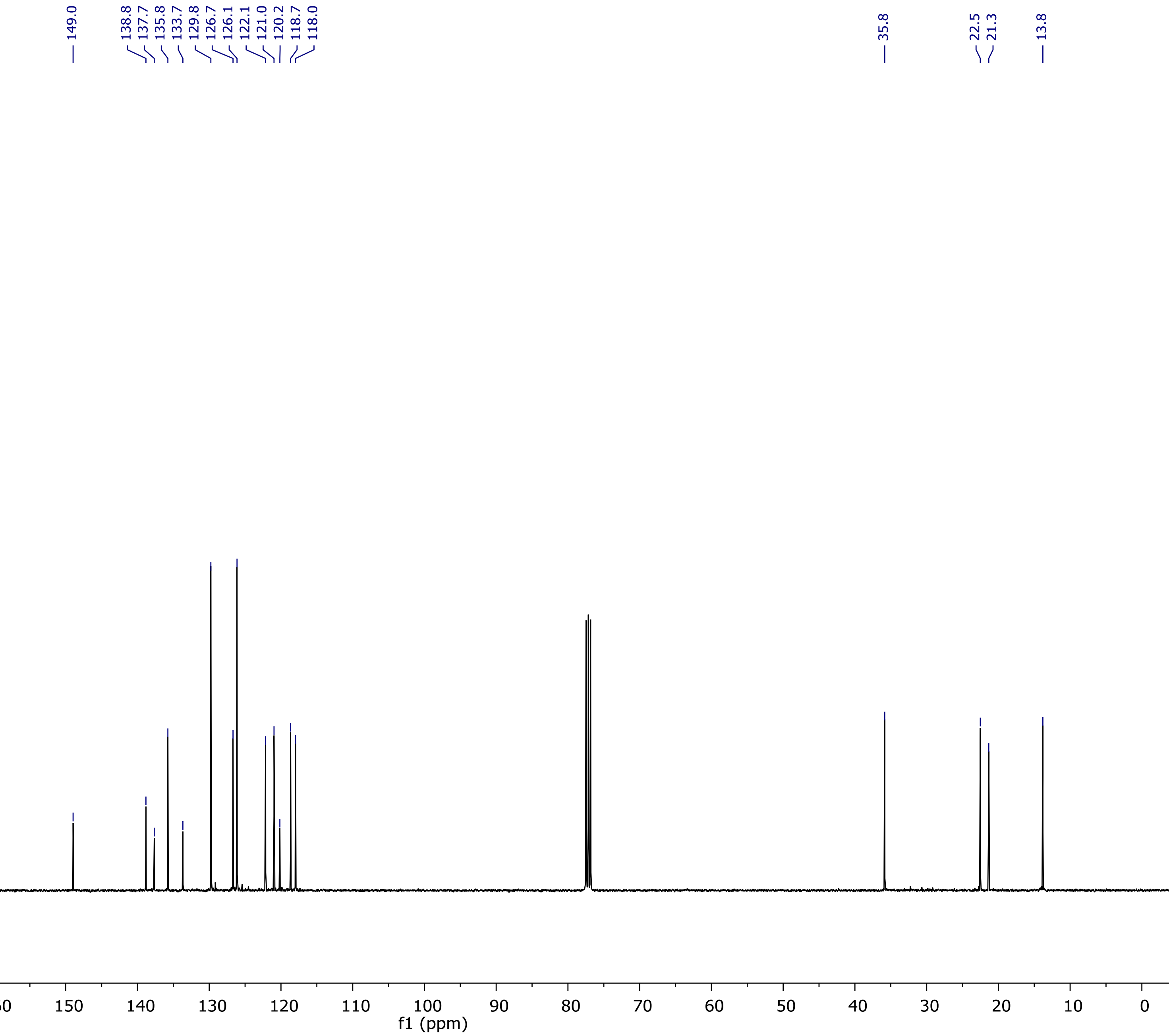

(*E*)-*N*,*N*-Dimethyl-4-[3-(pent-1-enyl)-2*H*-indazol-2-yl]aniline (2m)

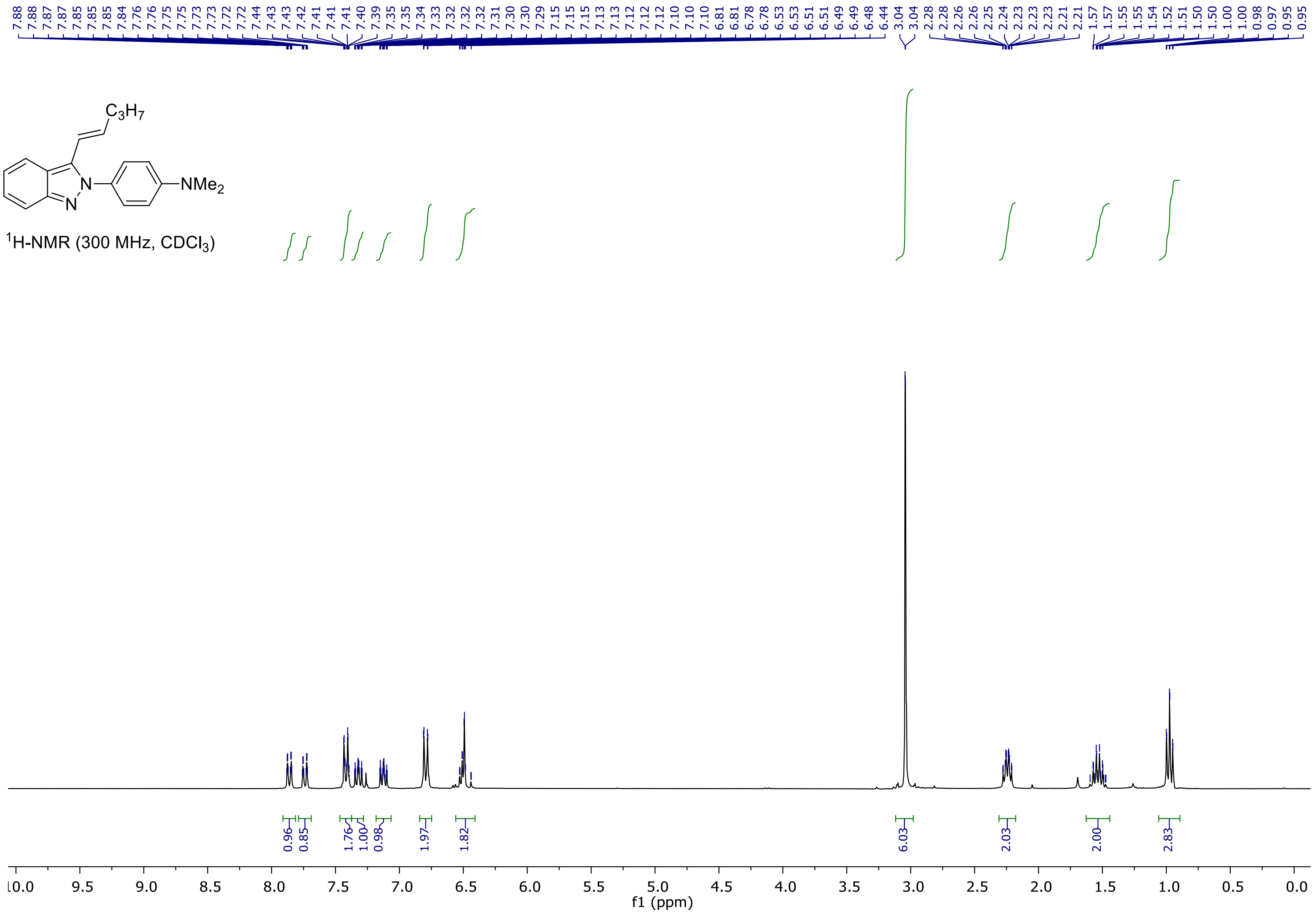

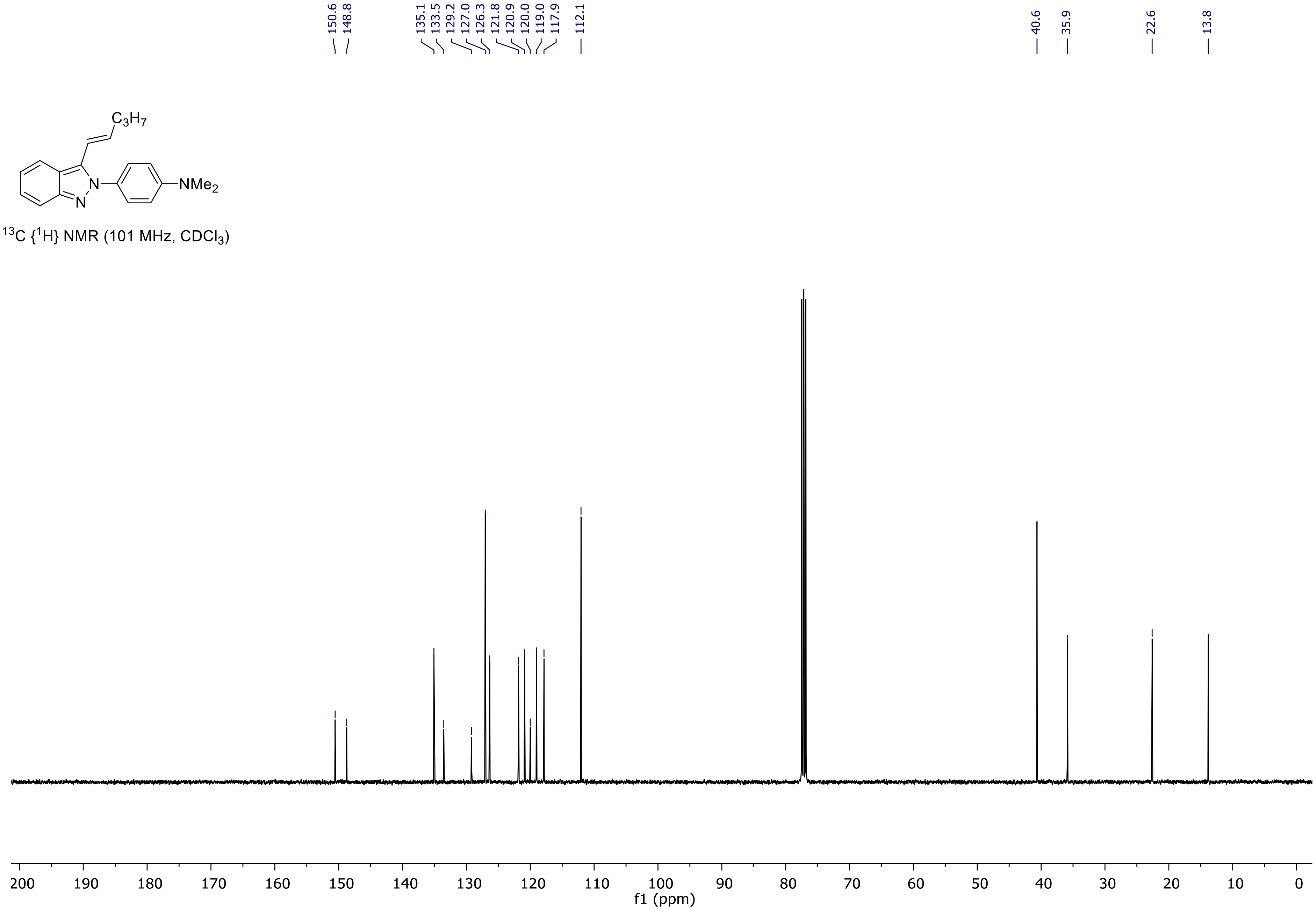

150.6
148.8
135.1
133.5
129.2
127.0
126.3
121.8
120.9
120.0
119.0
117.9
112.1
40.6
35.9
22.6
13.8
C_3H_7
N
N
NMe_2
$^{13}C\ \{^1H\}$ NMR (101 MHz, $CDCl_3$)
200 190 180 170 160 150 140 130 120 110 100 90 80 70 60 50 40 30 20 10 0
f1 (ppm)

(*E*)-*N*-Methyl-*N*-[4-(3-(pent-1-enyl)-2*H*-indazol-2-yl)phenyl]acetamide (2n)

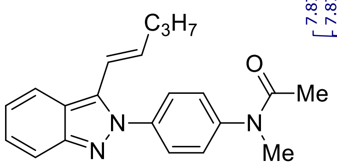

^{1}H-NMR (300 MHz, $CDCl_3$)

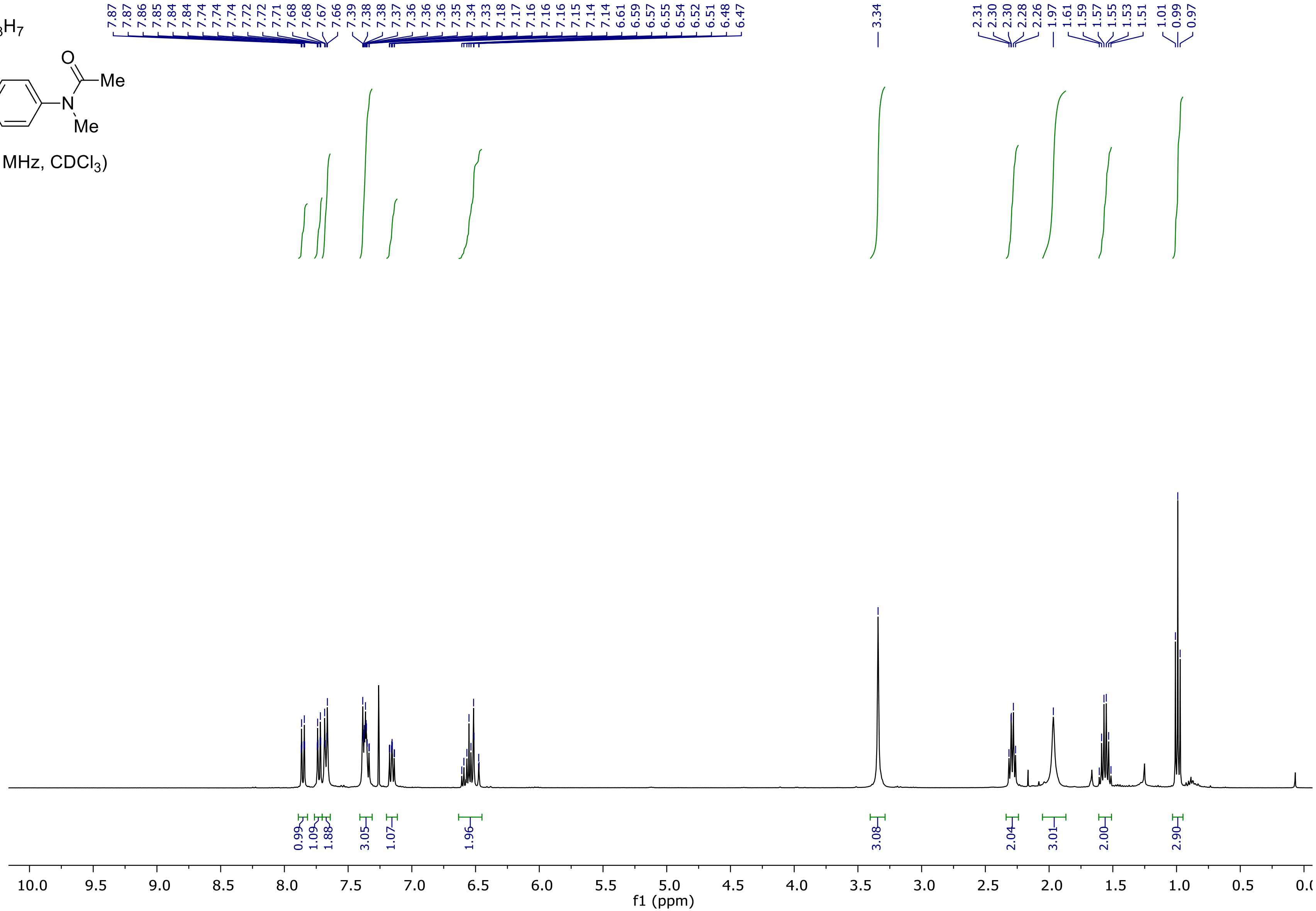

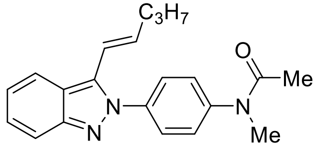

^{13}C {^{1}H} NMR (101 MHz, $CDCl_3$)

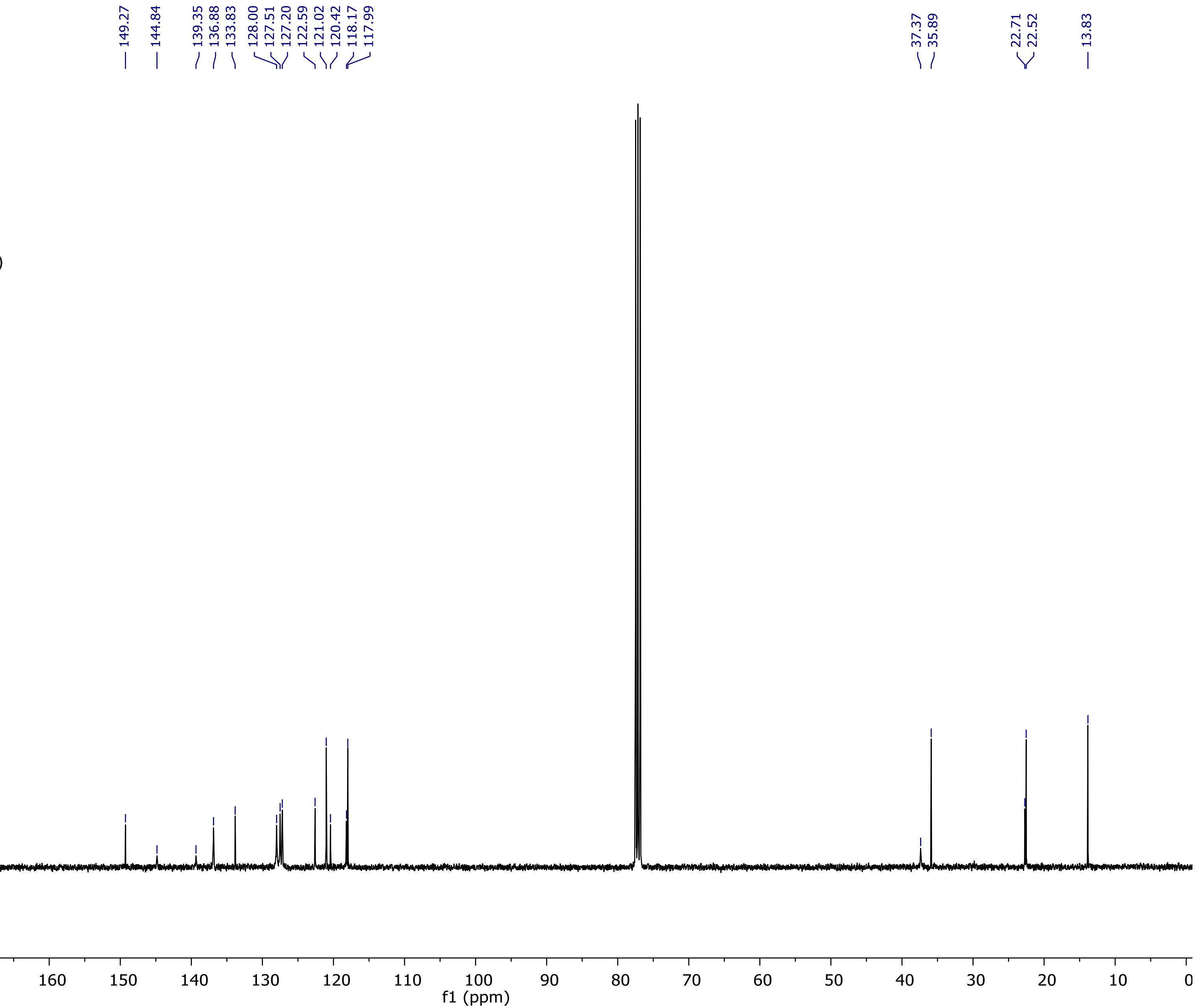

(*E*)-2-(4-Methoxyphenyl)-3-(pent-1-enyl)-2*H*-indazole (2o)

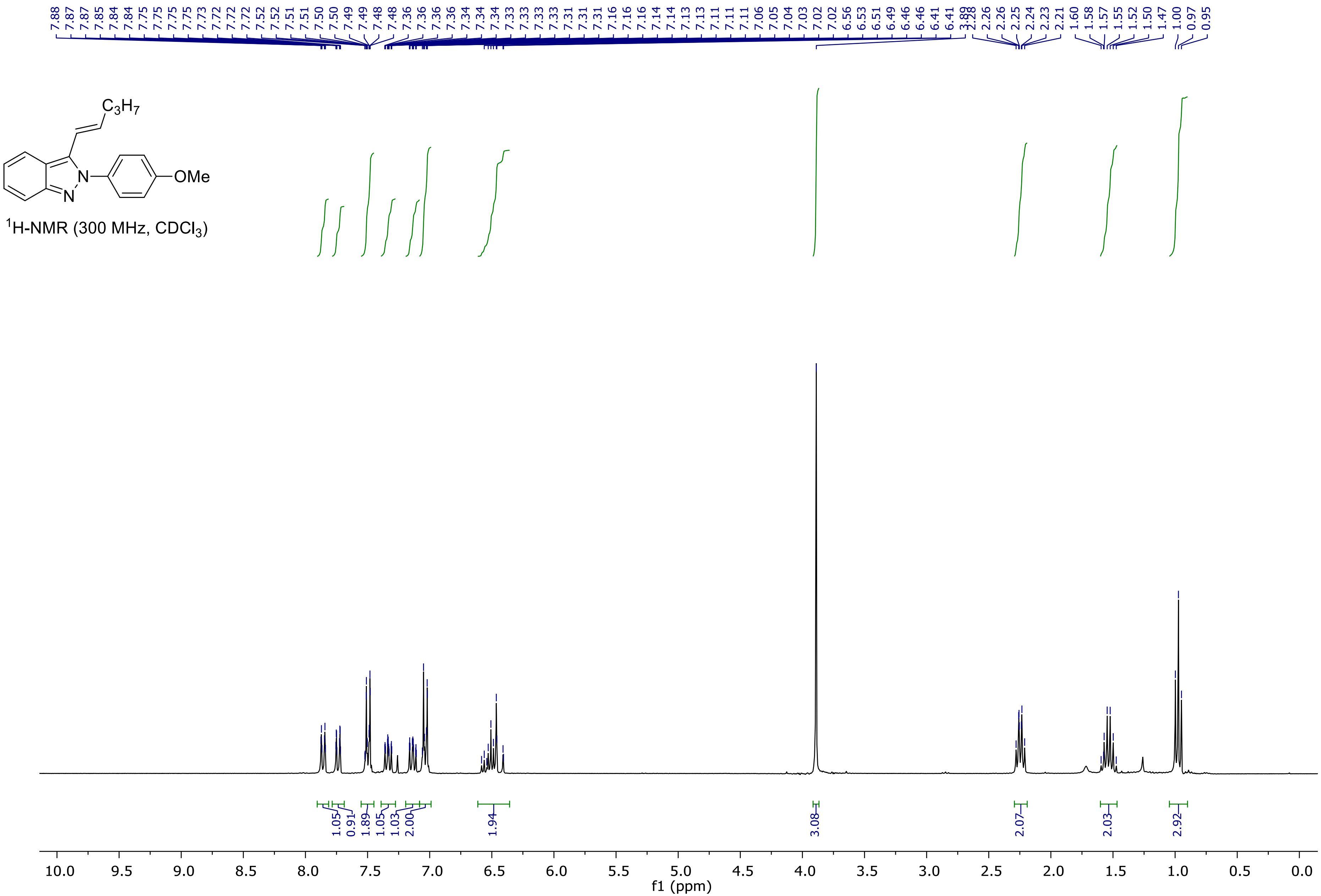

^{1}H-NMR (300 MHz, $CDCl_3$)

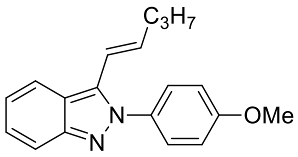

$^{13}C\ \{^1H\}$ NMR (75 MHz, $CDCl_3$)

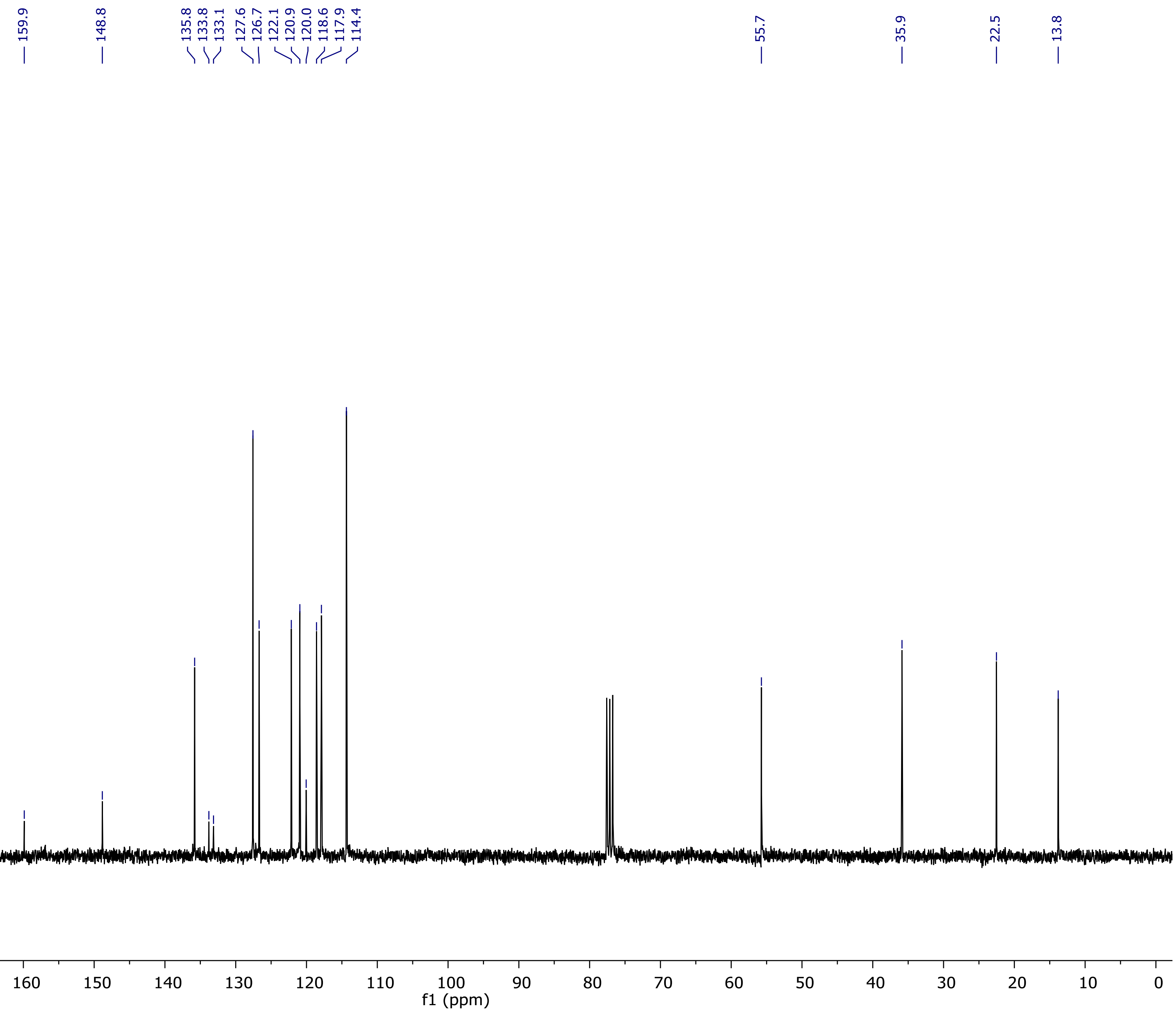

(*E*)-6-Chloro-3-(pent-1-enyl)-2-phenyl-2*H*-indazole (2p)

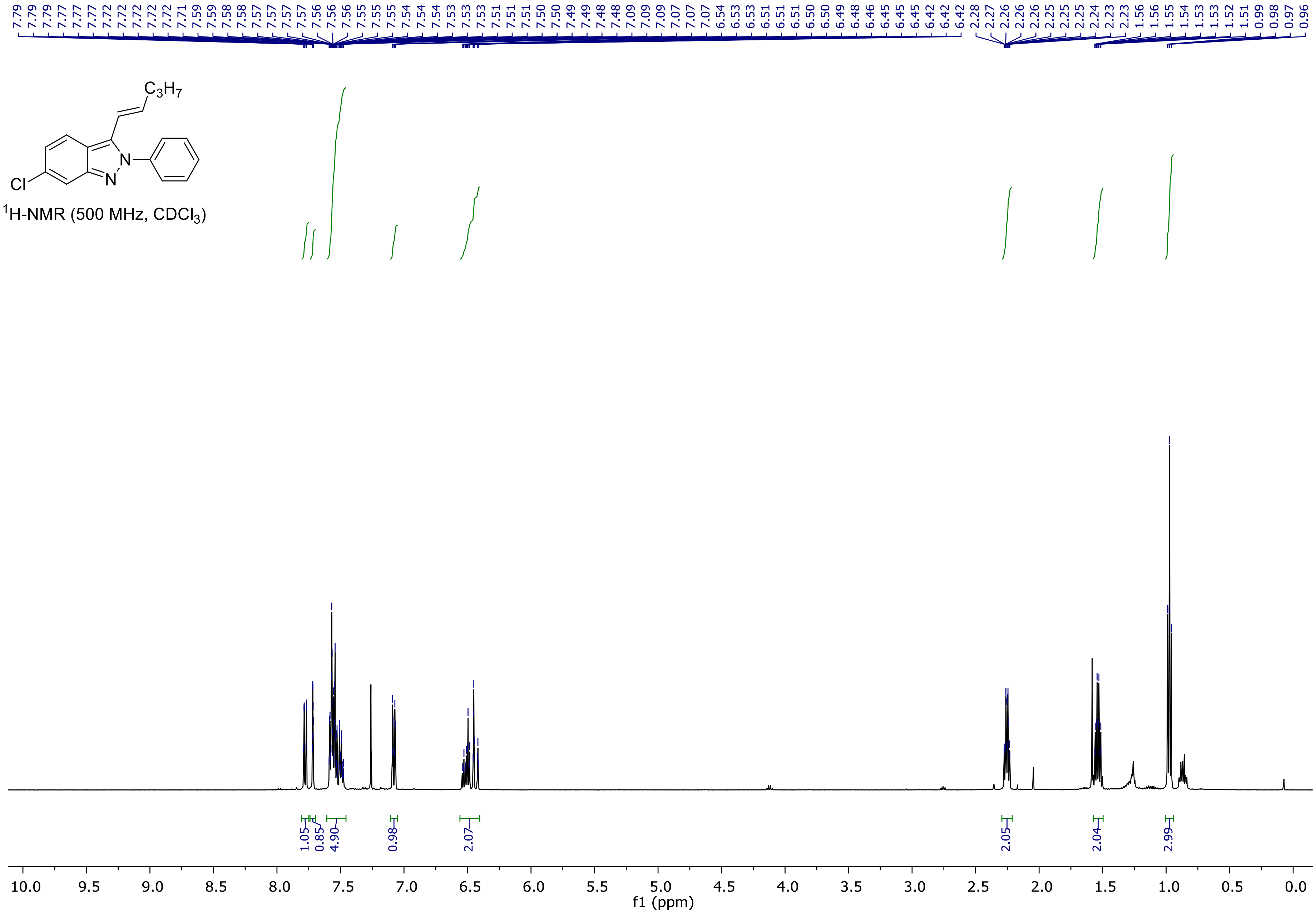

^{1}H-NMR (500 MHz, $CDCl_3$)

$^{13}C\{^1H\}$ NMR (126 MHz, $CDCl_3$)

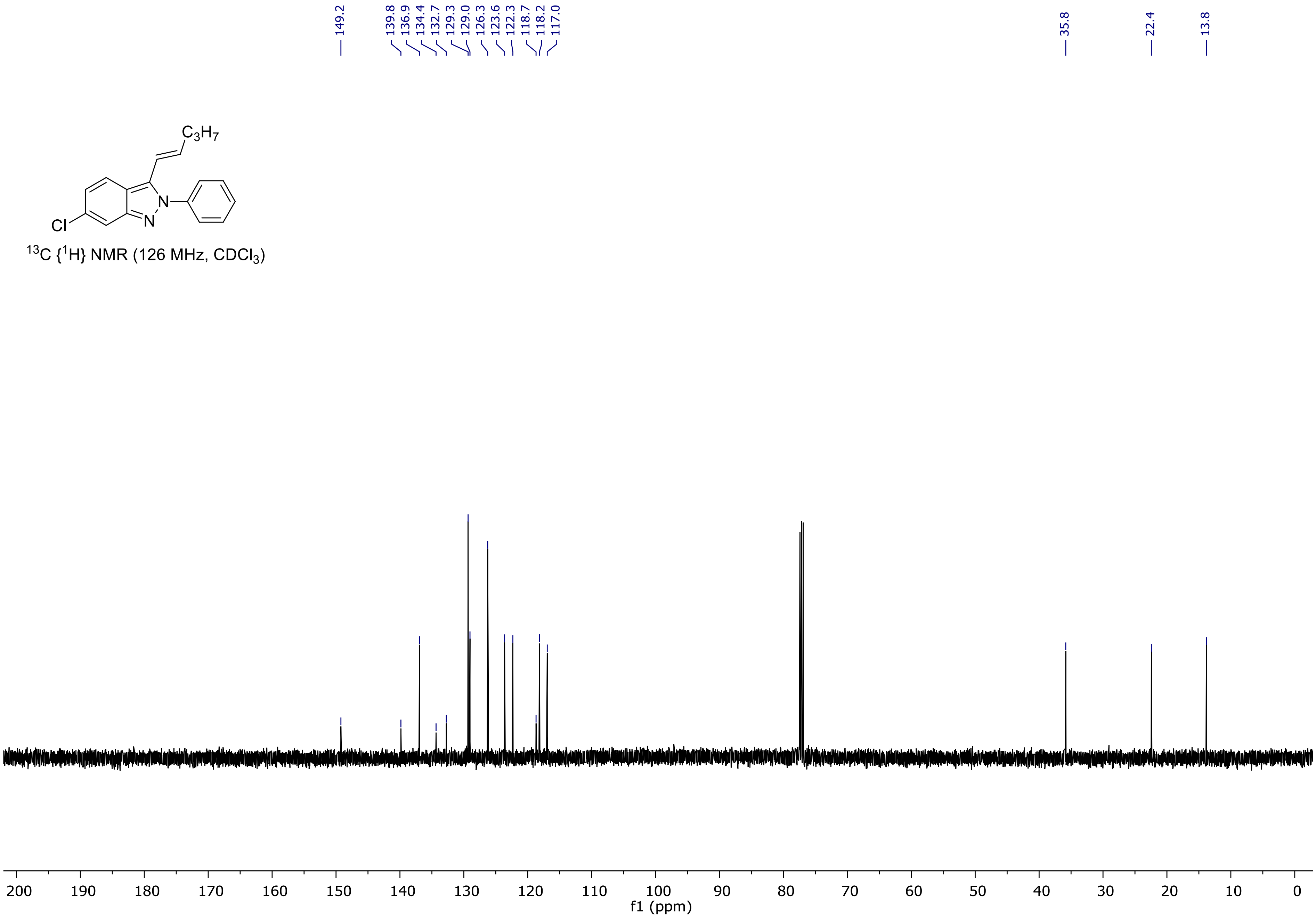

(*E*)-3-(Pent-1-enyl)-2-phenyl-5-(trifluoromethyl)-2*H*-indazole (2q)

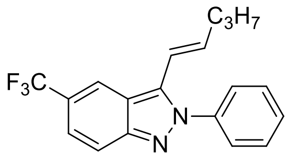

^{1}H-NMR (400 MHz, $CDCl_3$)

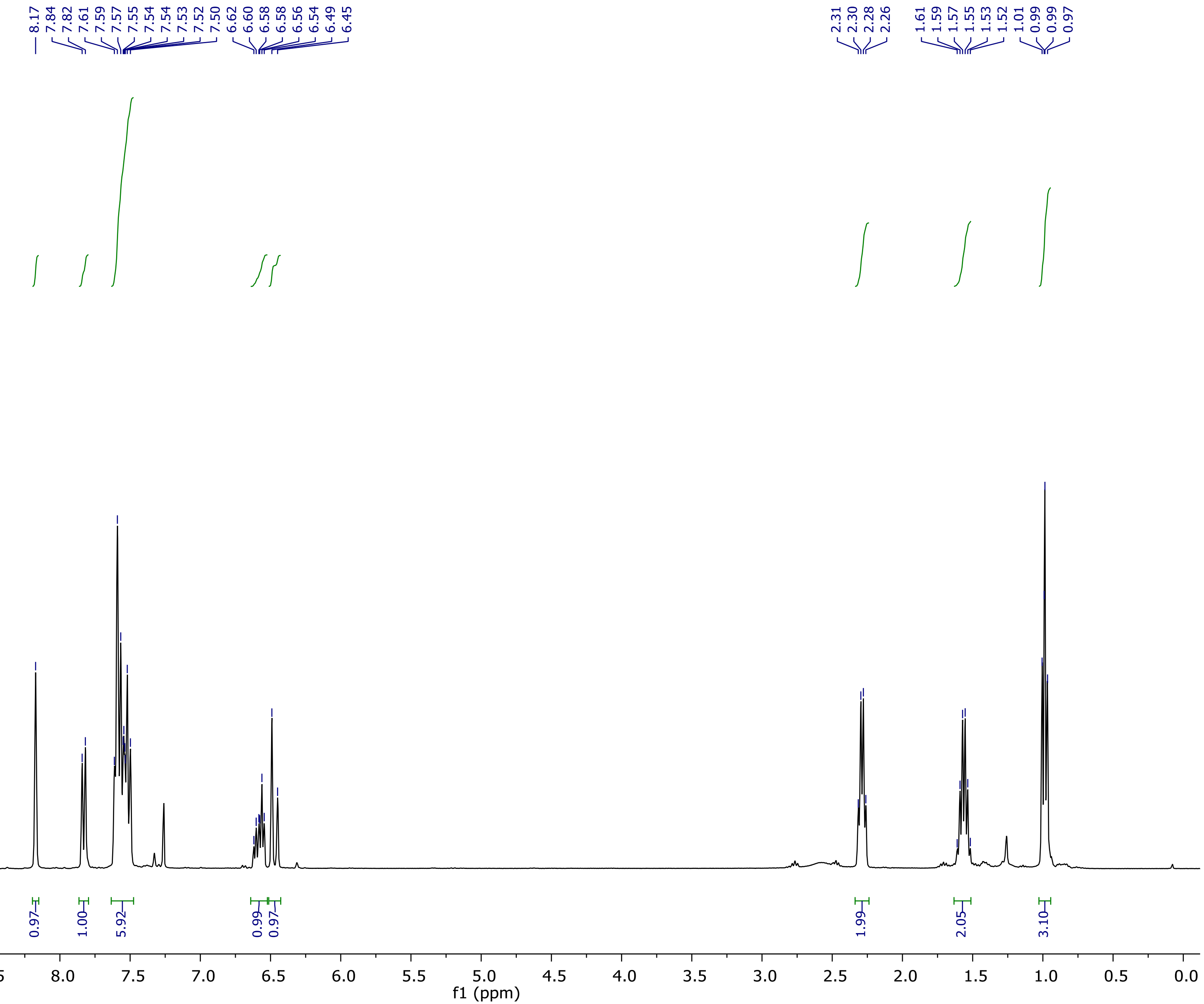

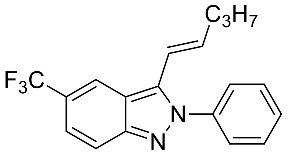

^{19}F-NMR (376 MHz, $CDCl_3$)

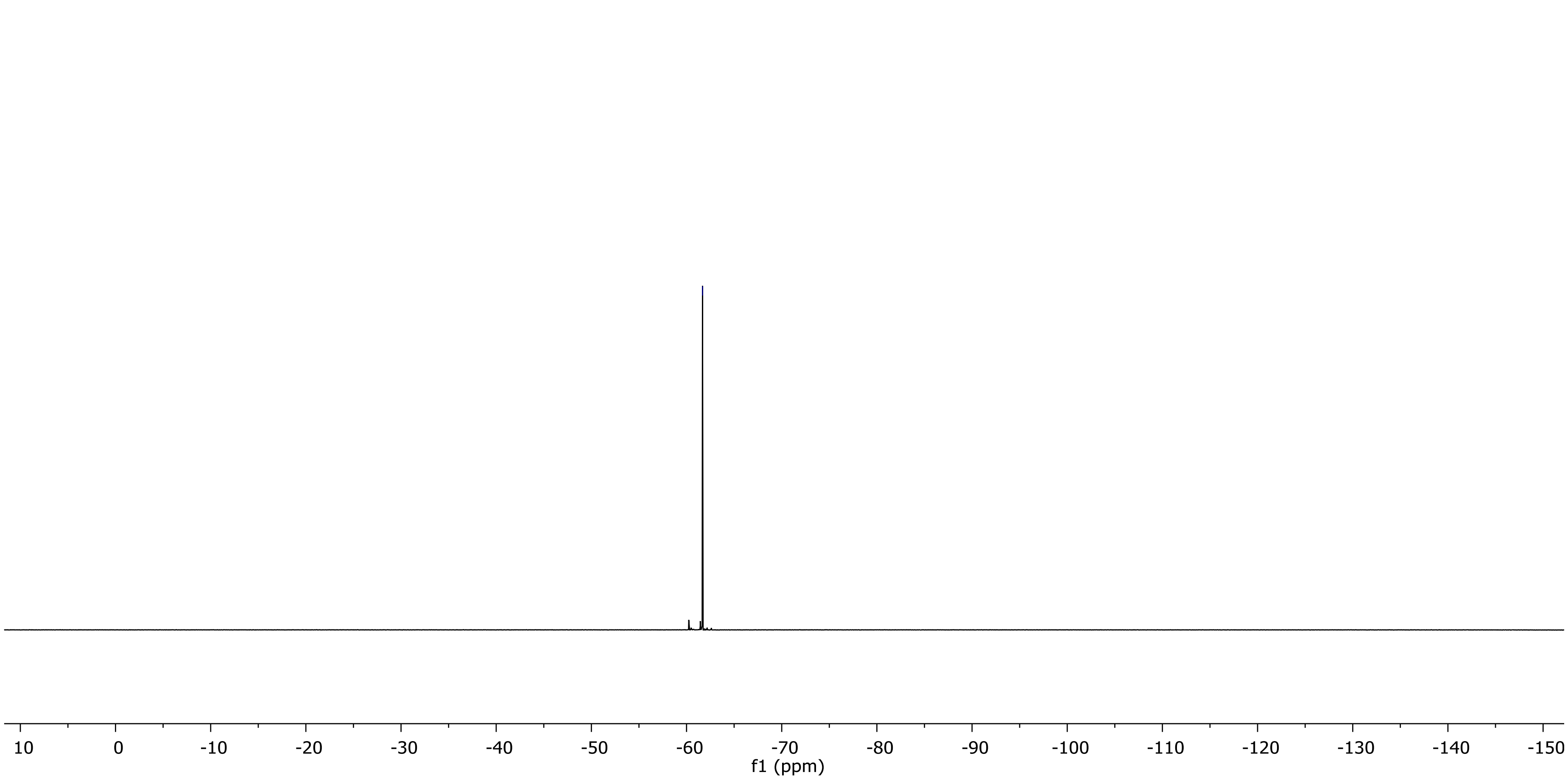

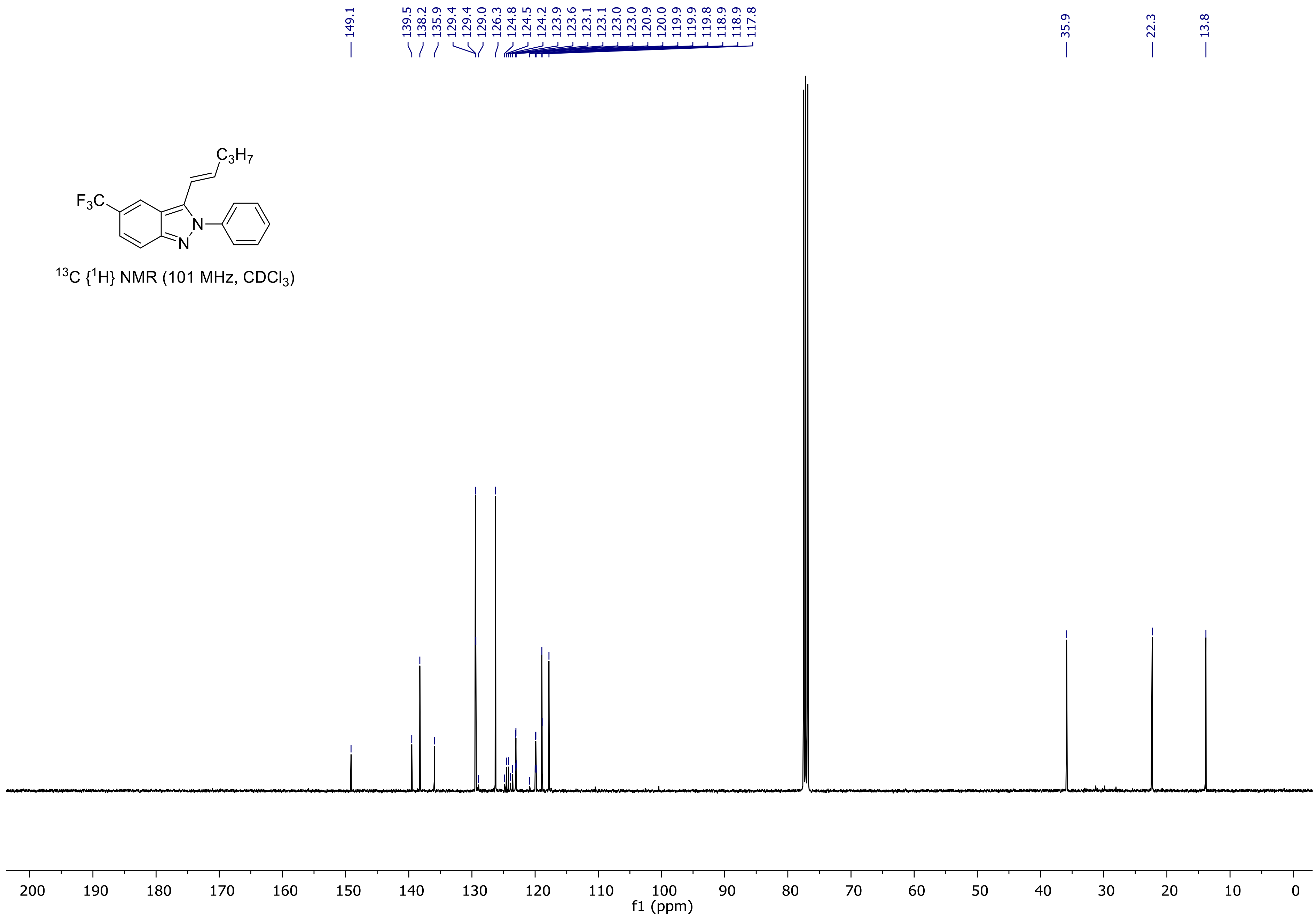
149.1
139.5
138.2
135.9
129.4
129.4
129.0
126.3
124.8
124.5
124.2
123.9
123.6
123.1
123.1
123.0
123.0
120.9
120.0
119.9
119.9
119.8
118.9
118.9
117.8
35.9
22.3
13.8
C_3H_7
F_3C
N
N
^{13}C {^1H} NMR (101 MHz, $CDCl_3$)
200 190 180 170 160 150 140 130 120 110 100 90 80 70 60 50 40 30 20 10 0
f1 (ppm)

Methyl (*E*)-3-(pent-1-enyl)-2-phenyl-2*H*-indazole-5-carboxylate (2r)

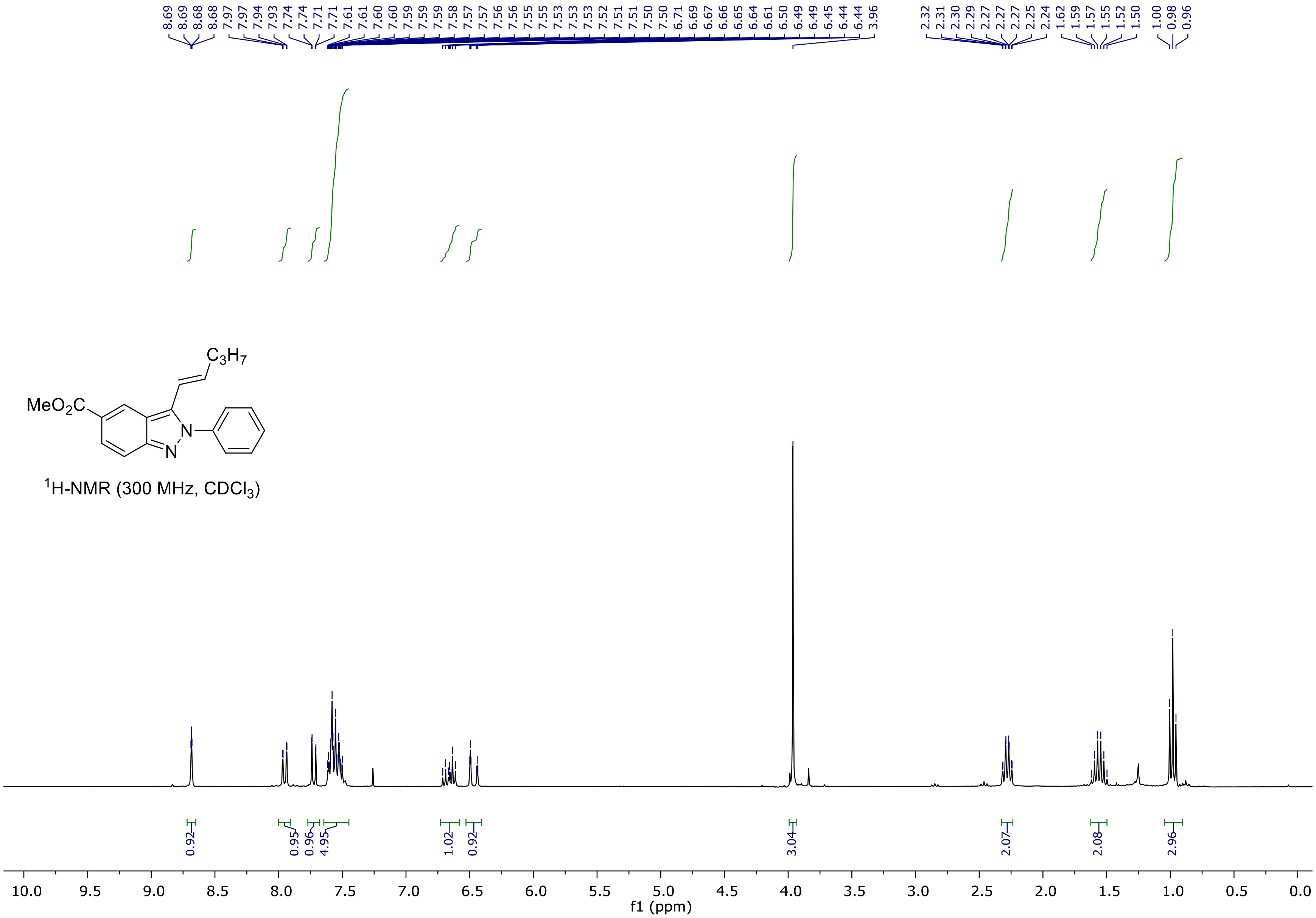

^{1}H-NMR (300 MHz, $CDCl_3$)

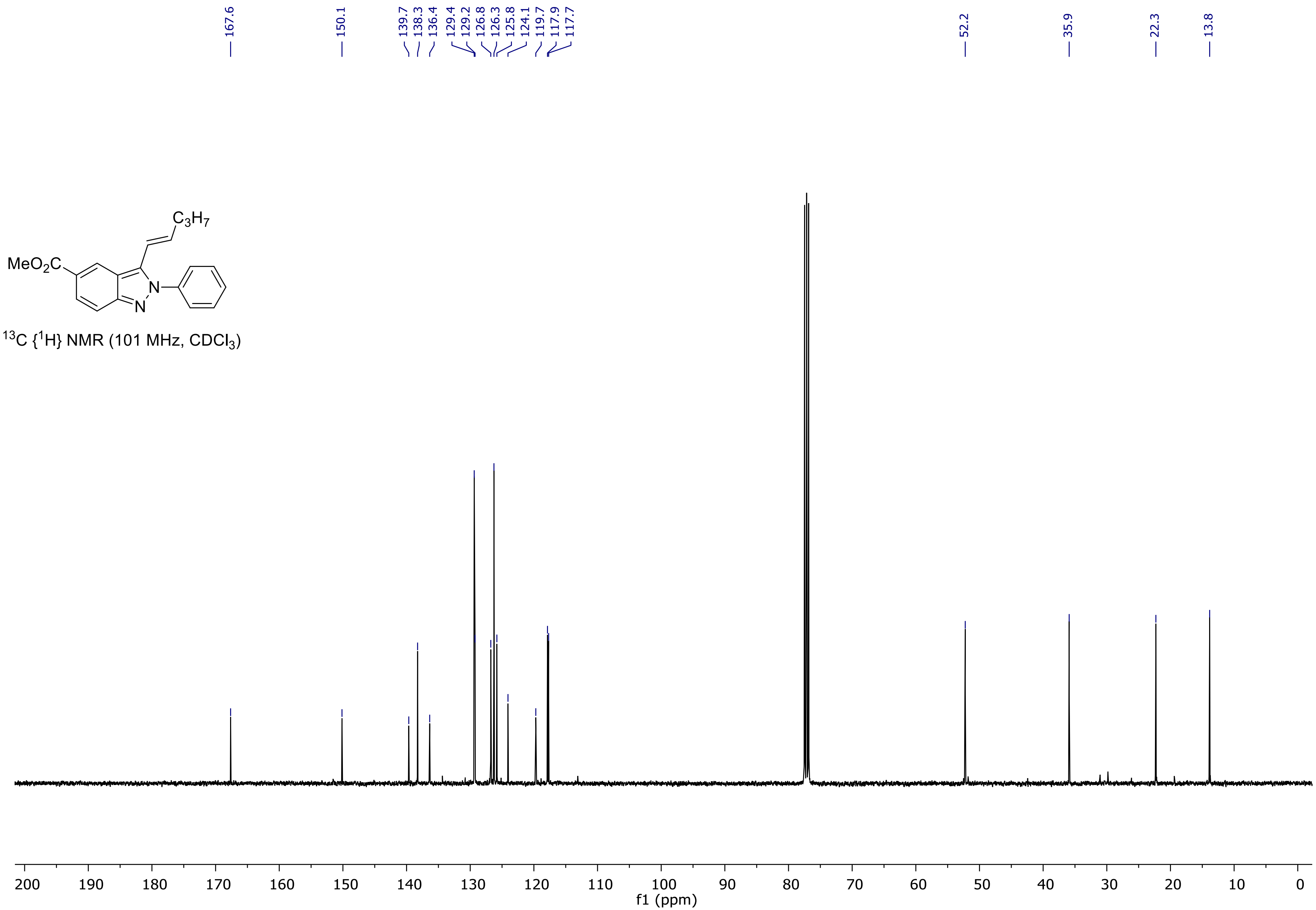

167.6
150.1
139.7
138.3
136.4
129.4
129.2
126.8
126.3
125.8
124.1
119.7
117.9
117.7
52.2
35.9
22.3
13.8
C_3H_7
MeO_2C
N
N
^{13}C {^1H} NMR (101 MHz, $CDCl_3$)
200
190
180
170
160
150
140
130
120
110
100
90
80
70
60
50
40
30
20
10
0
f1 (ppm)

(*E*)-5-Methyl-3-(pent-1-enyl)-2-phenyl-2*H*-indazole (2s)

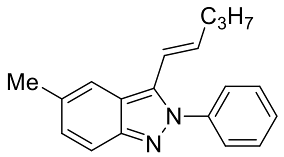

^{1}H-NMR (300 MHz, $CDCl_3$)

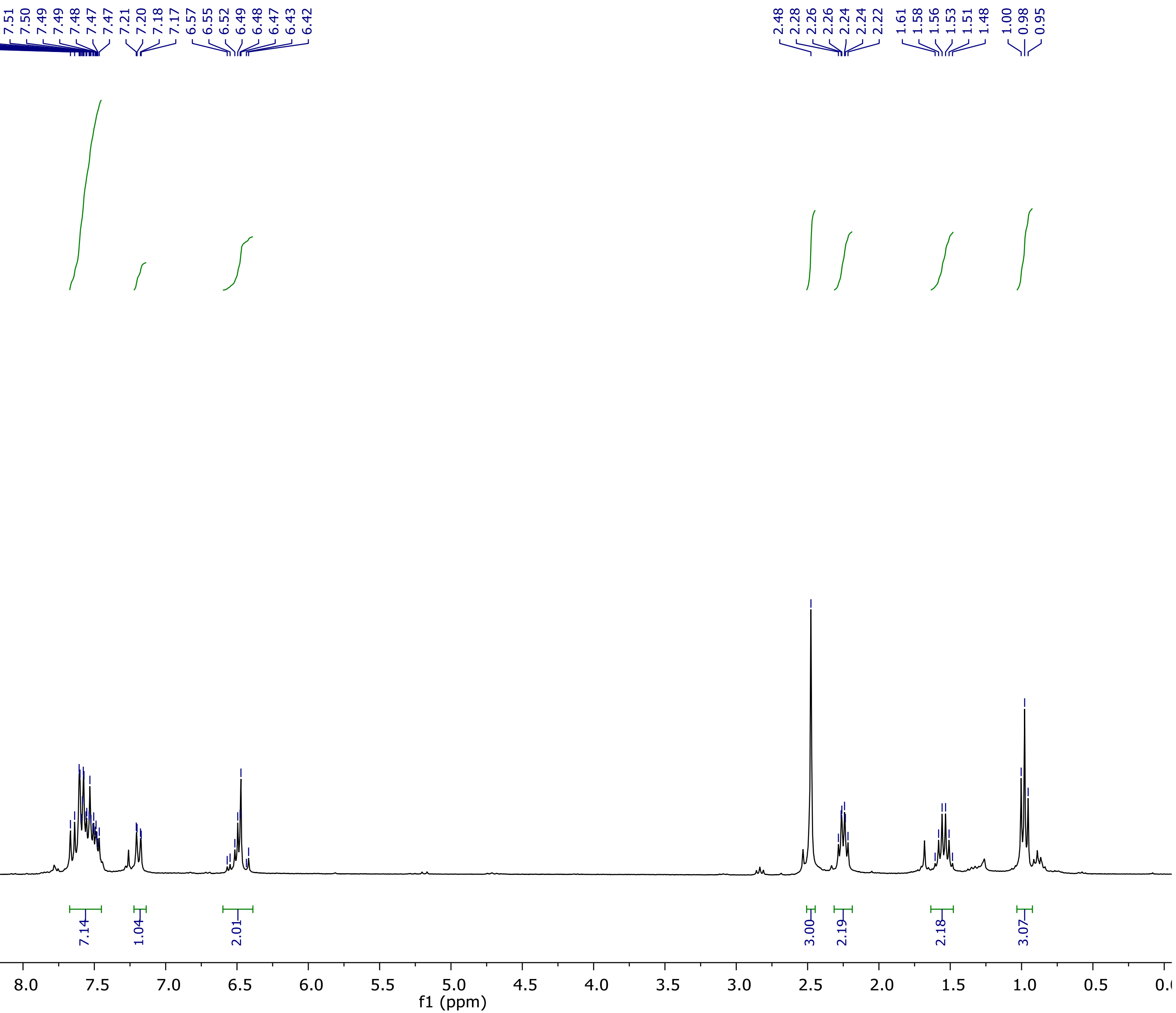

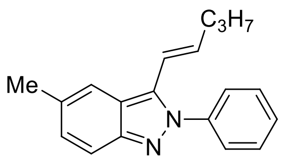

$^{13}C\{^1H\}$ NMR (101 MHz, $CDCl_3$)

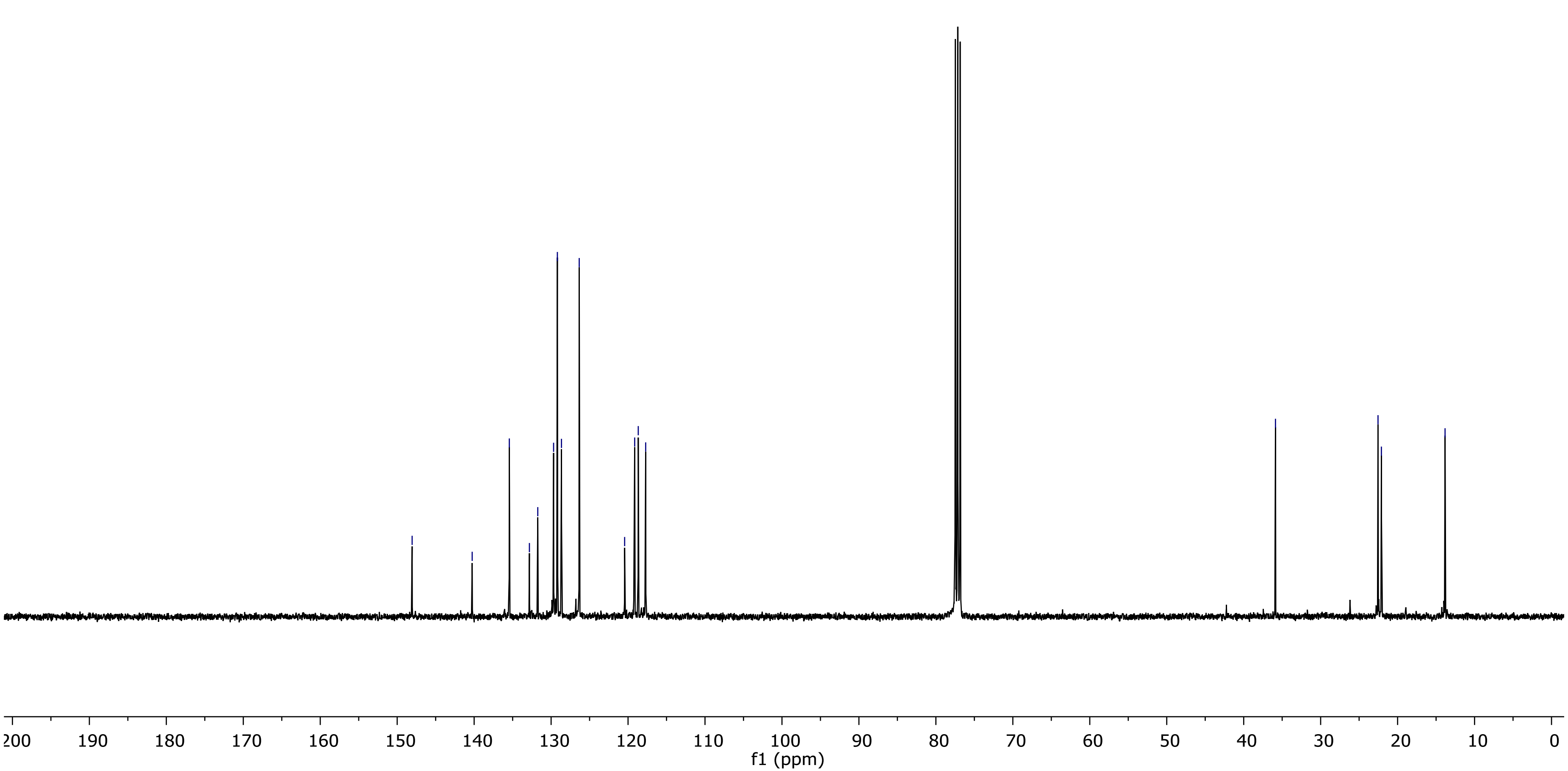

(*E*)-4-Methyl-3-(pent-1-enyl)-2-phenyl-2*H*-indazole (2t)

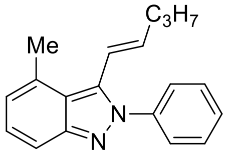

^{1}H-NMR (400 MHz, $CDCl_3$)

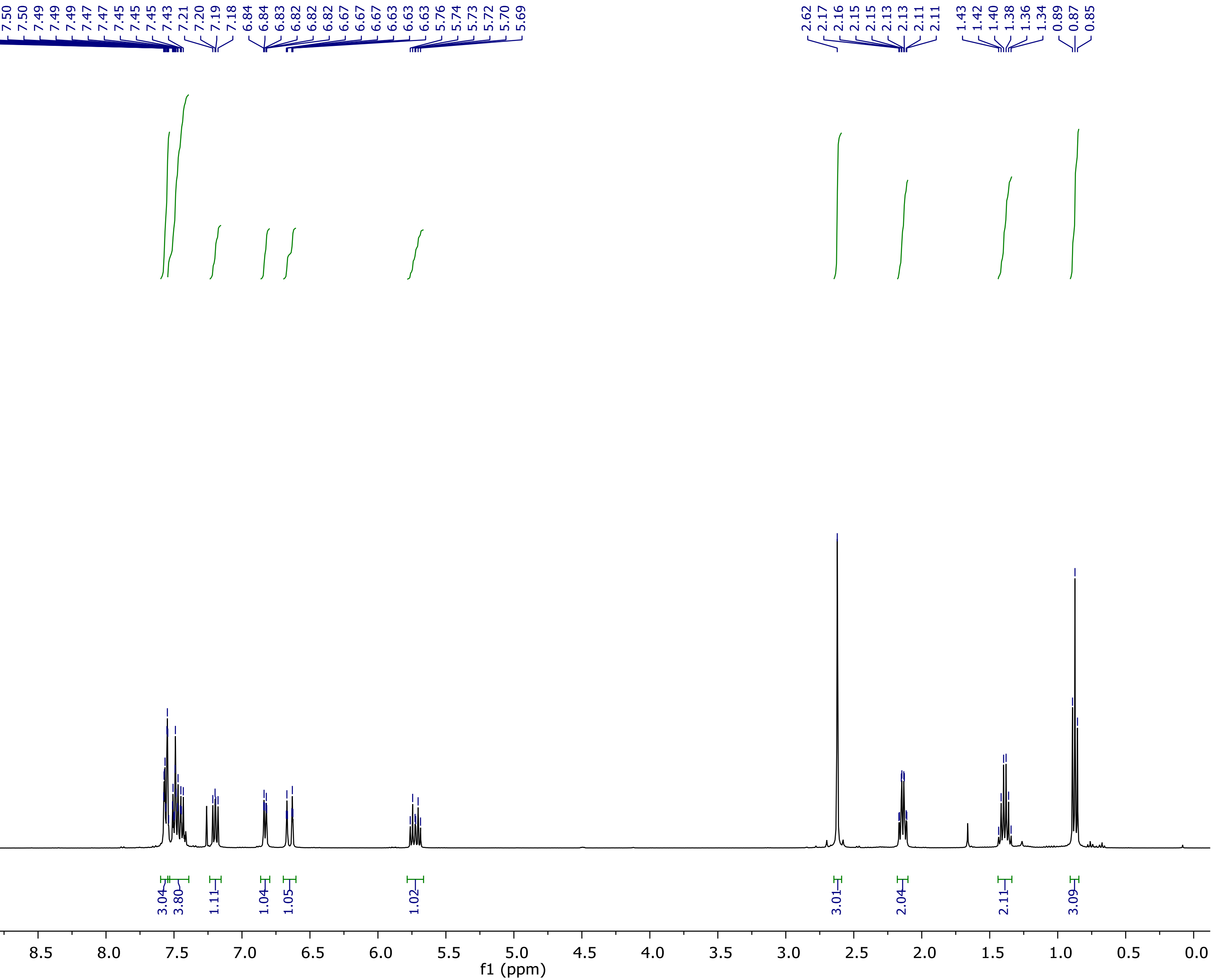

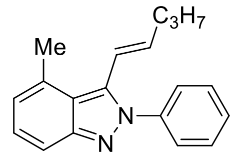

$^{13}C\{^1H\}$ NMR (101 MHz, $CDCl_3$)

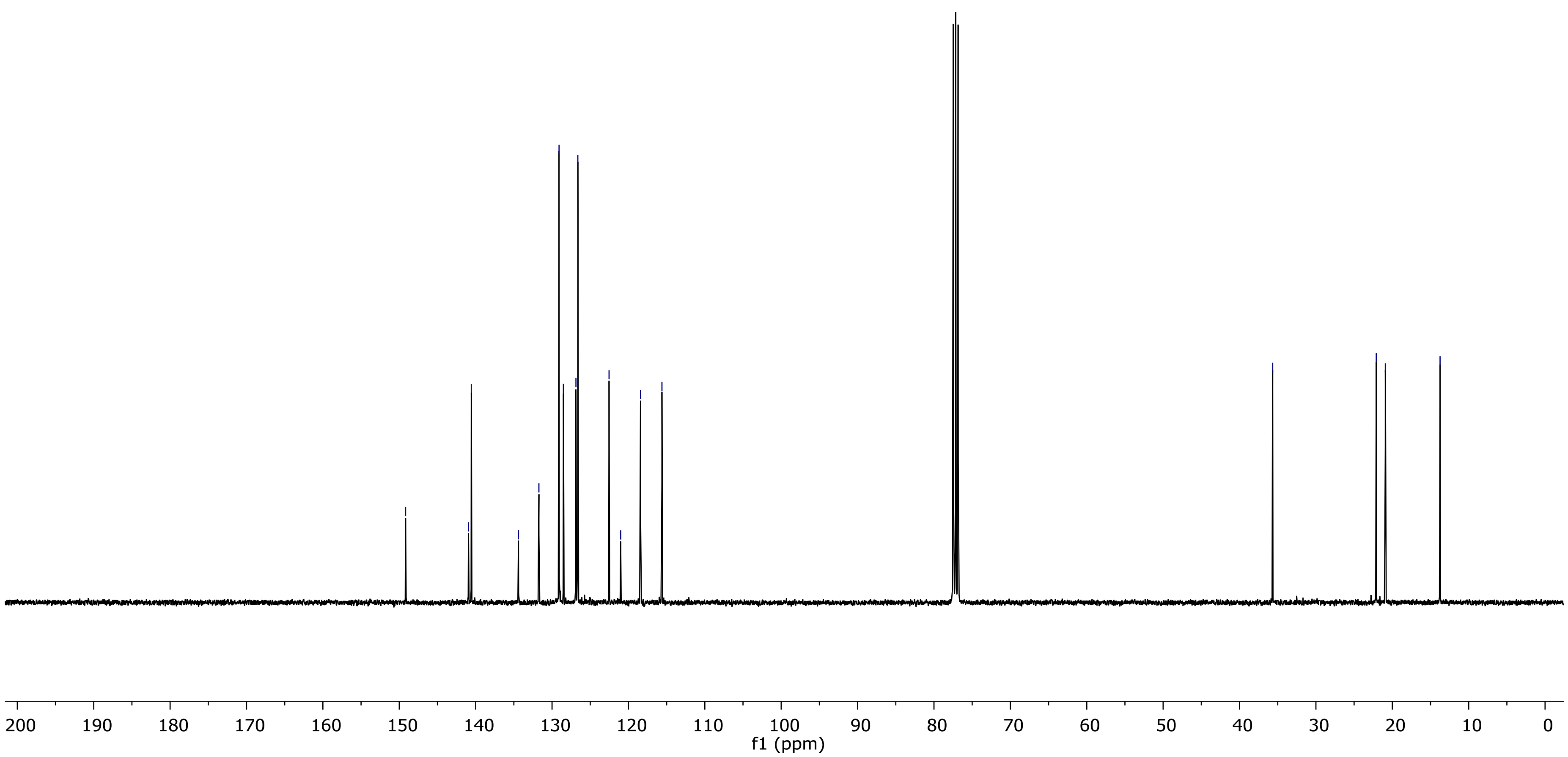

(*E*)-3-(4-Methylpent-1-en-1-yl)-2-phenyl-2*H*-indazole (2u)

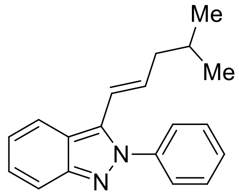

^{1}H-NMR (400 MHz, $CDCl_3$)

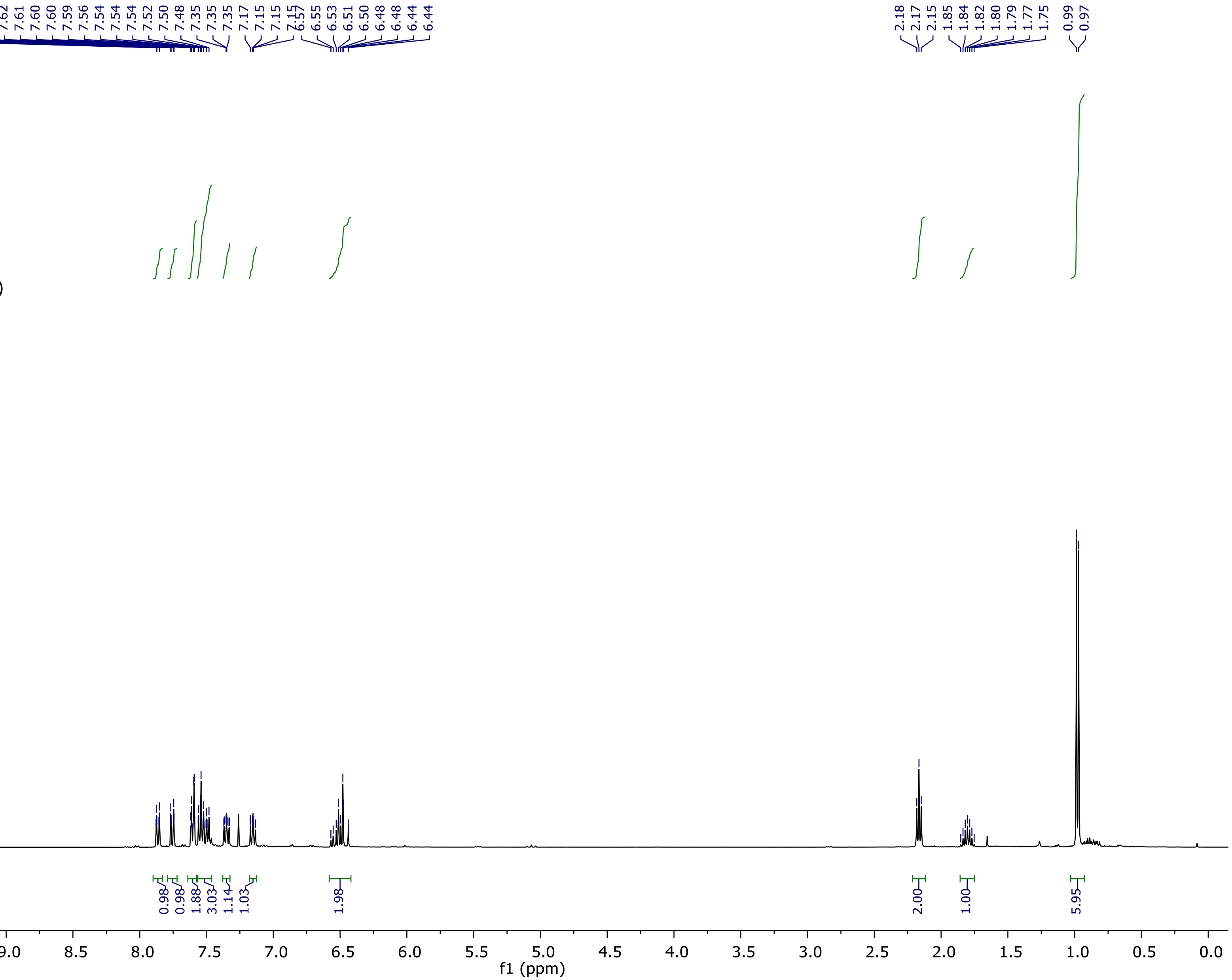

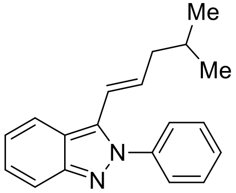

^{13}C {^{1}H} NMR (101 MHz, $CDCl_3$)

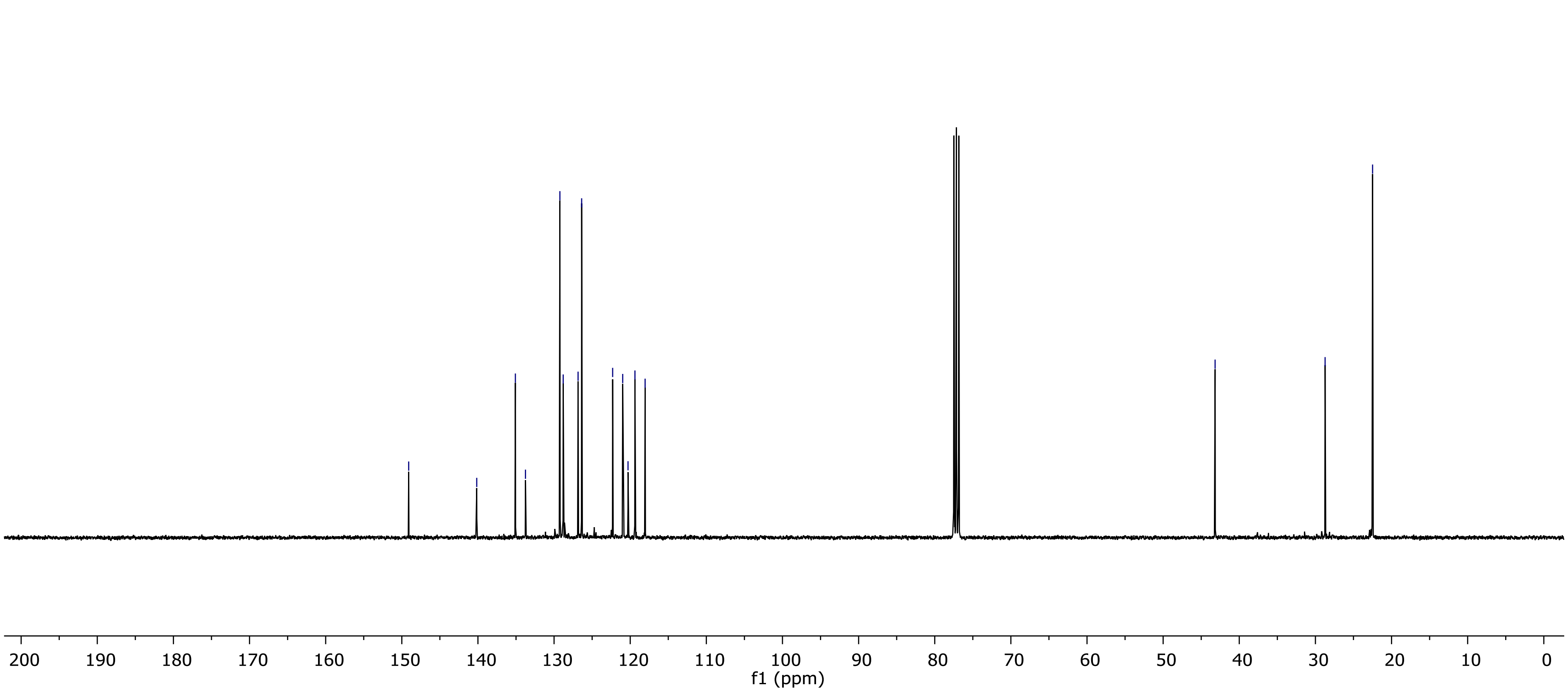

(*E*)-3-(4-Chlorobut-1-enyl)-2-phenyl-2*H*-indazole (2v)

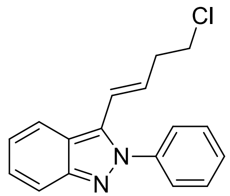

^{1}H-NMR (400 MHz, $CDCl_3$)

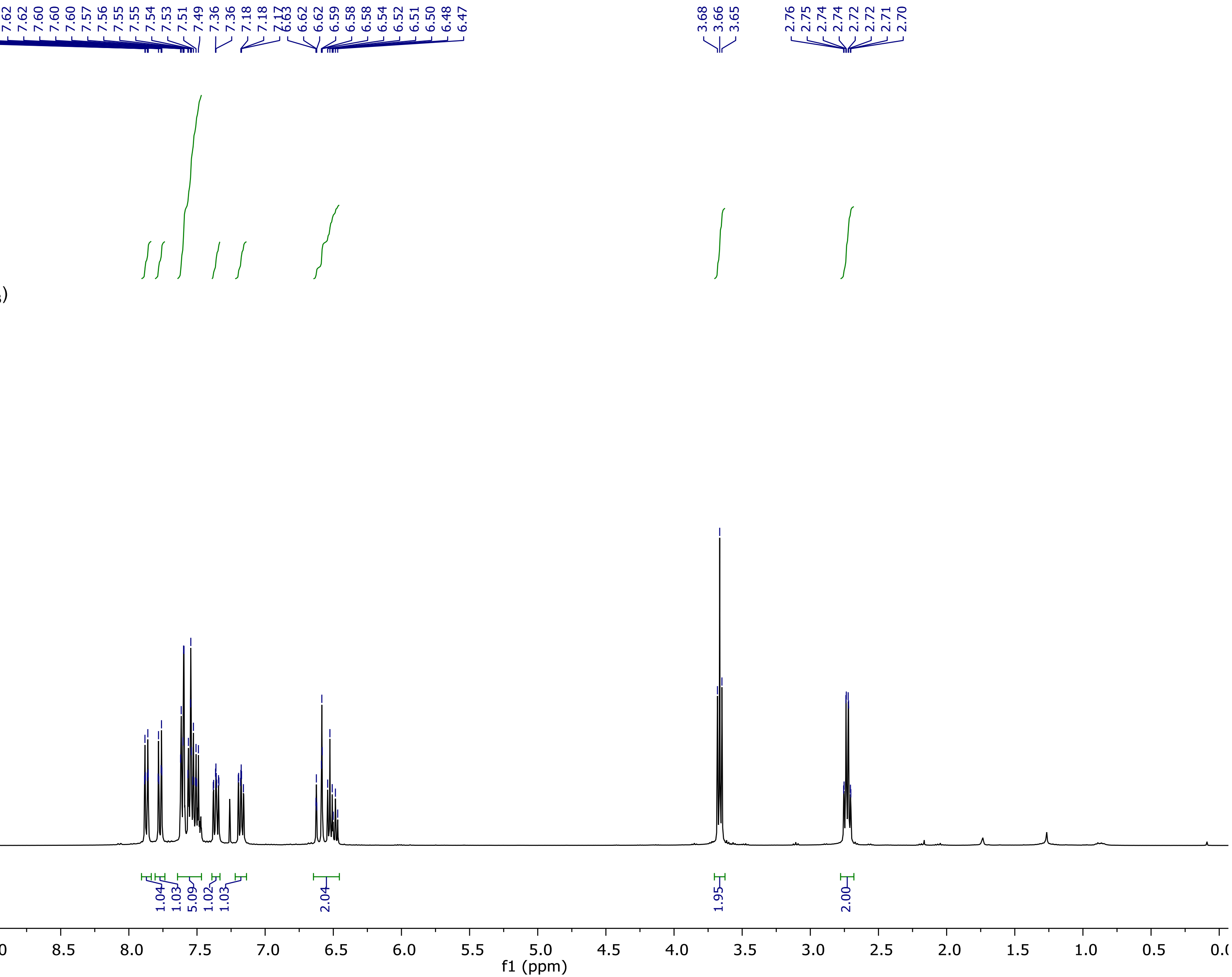

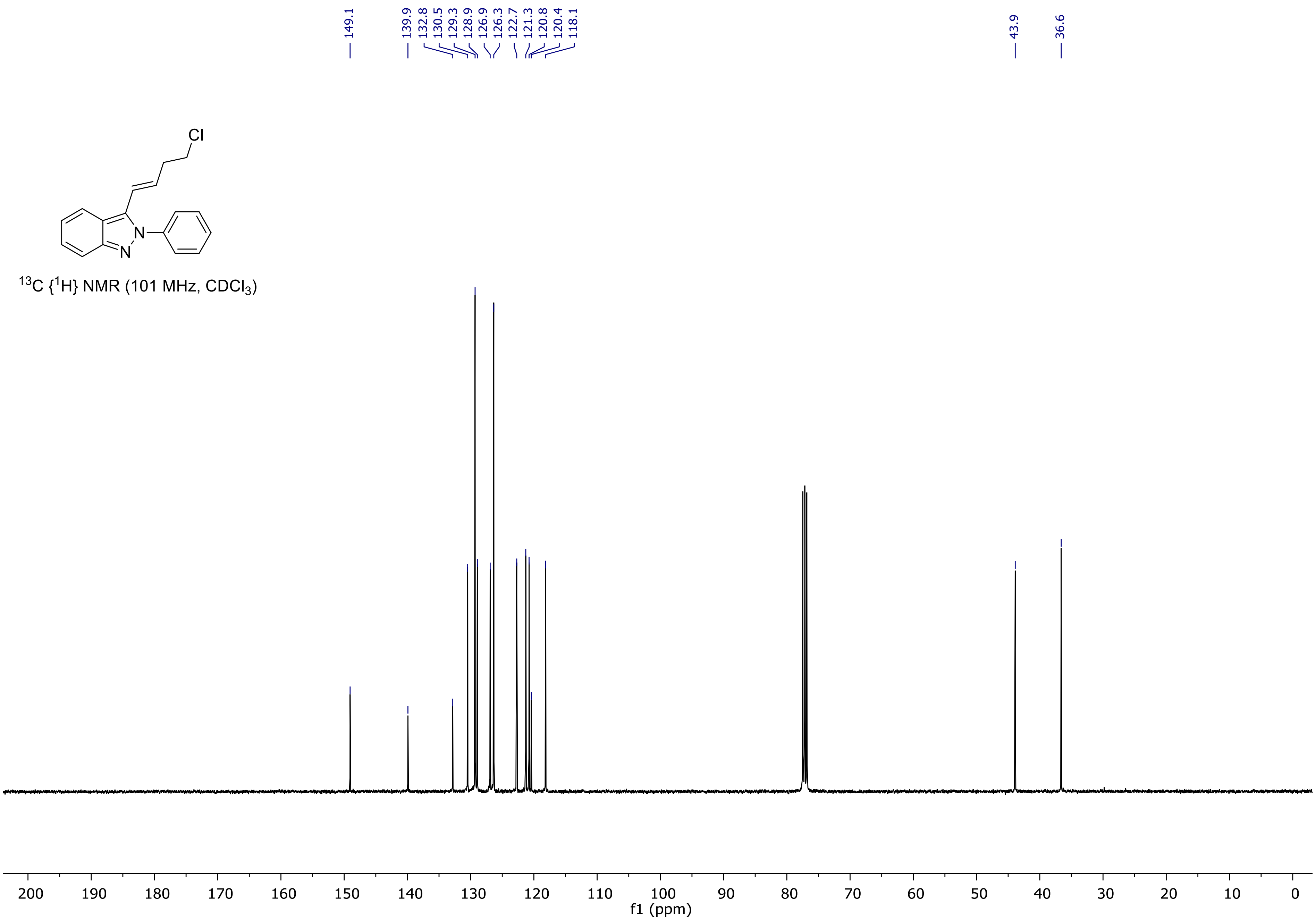

$^{13}C\ \{^1H\}$ NMR (101 MHz, $CDCl_3$)

(*E*)-3-(2-Cyclohexylvinyl)-2-phenyl-2*H*-indazole (2w)

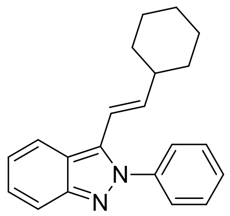

^{1}H-NMR (300 MHz, $CDCl_3$)

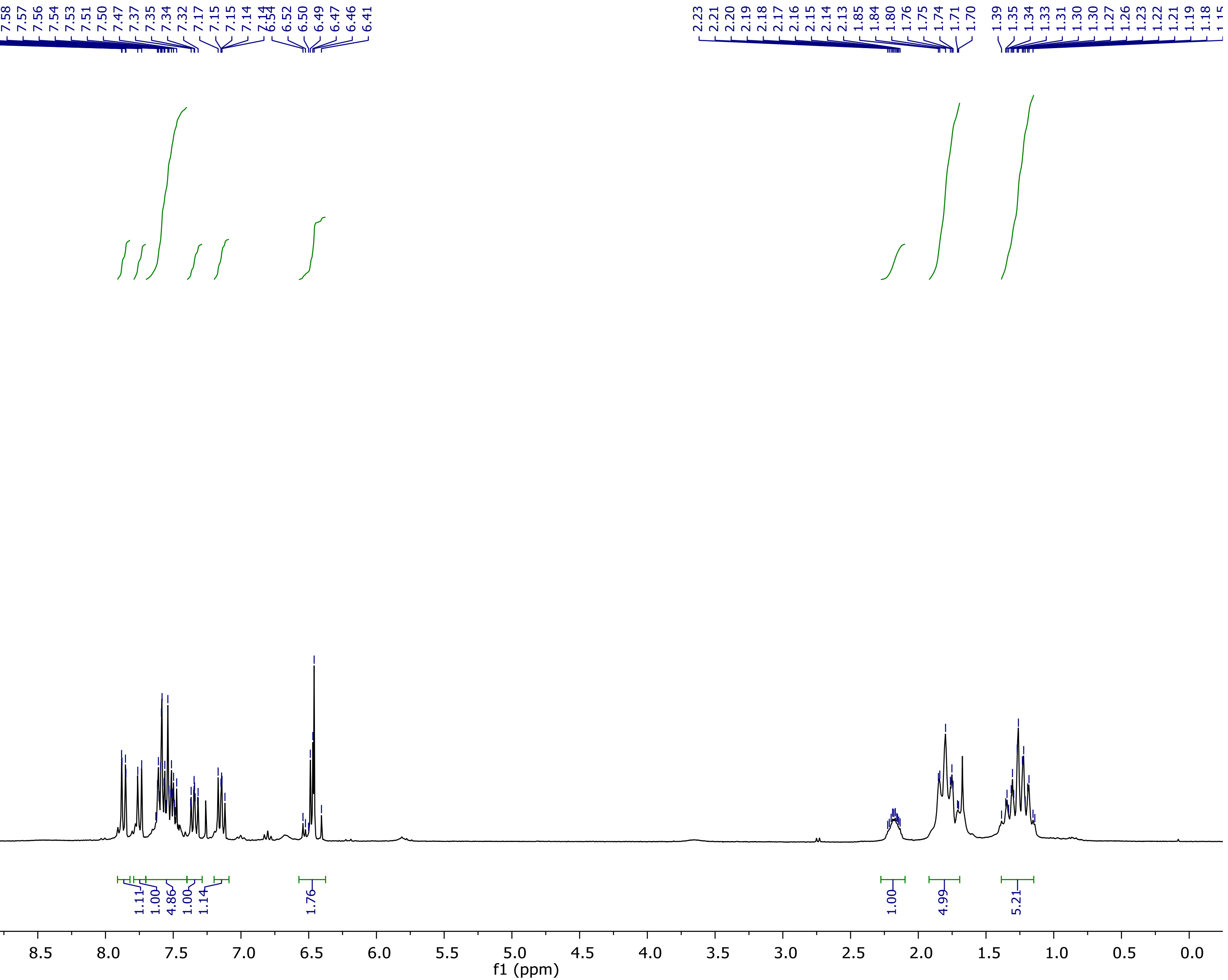

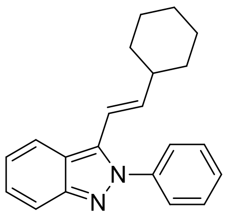

^{13}C {^{1}H} NMR (101 MHz, $CDCl_3$)

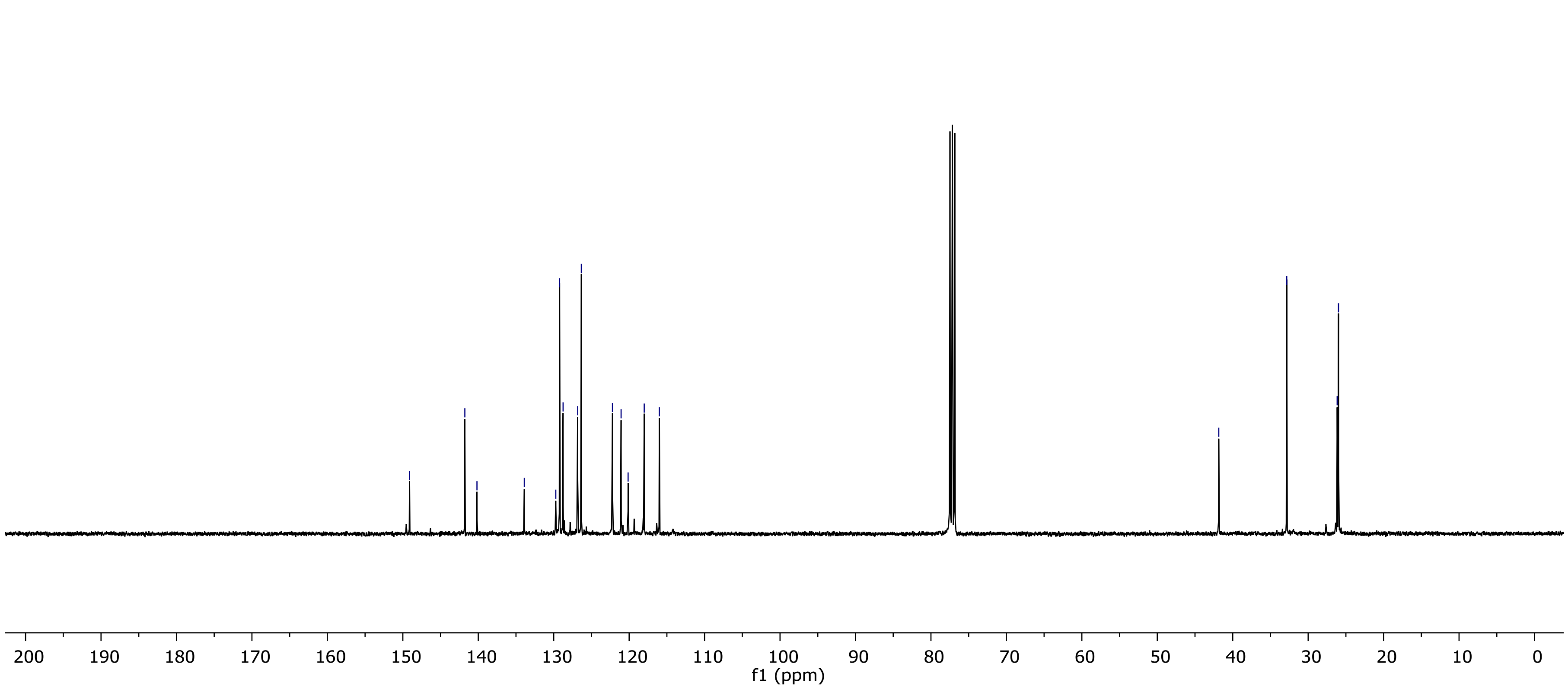

(*E*)-2-Phenyl-3-styryl-2*H*-indazole (2x)

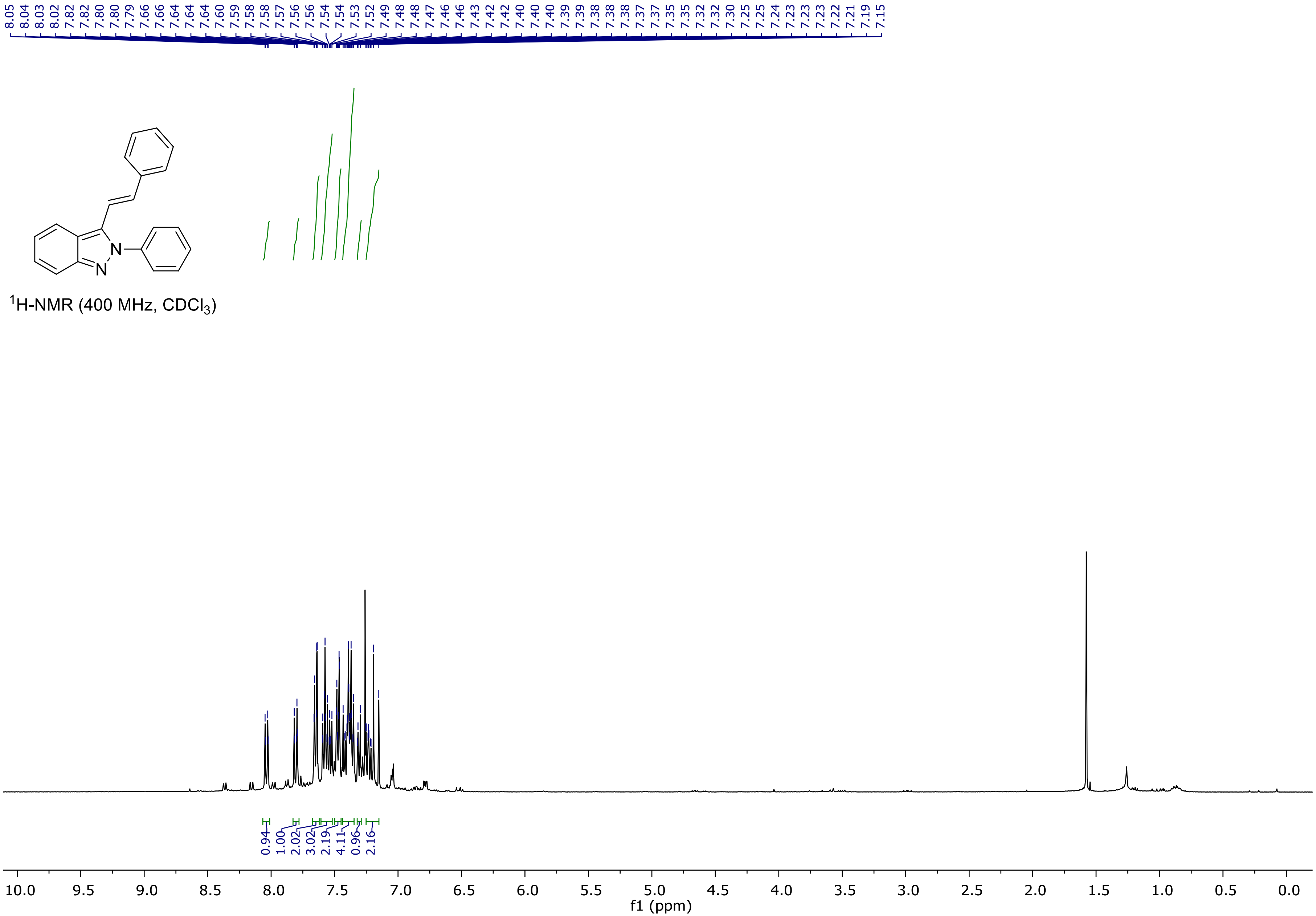

^{1}H-NMR (400 MHz, $CDCl_3$)

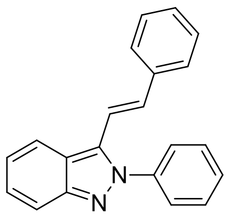

^{13}C {^{1}H} NMR (101 MHz, $CDCl_3$)

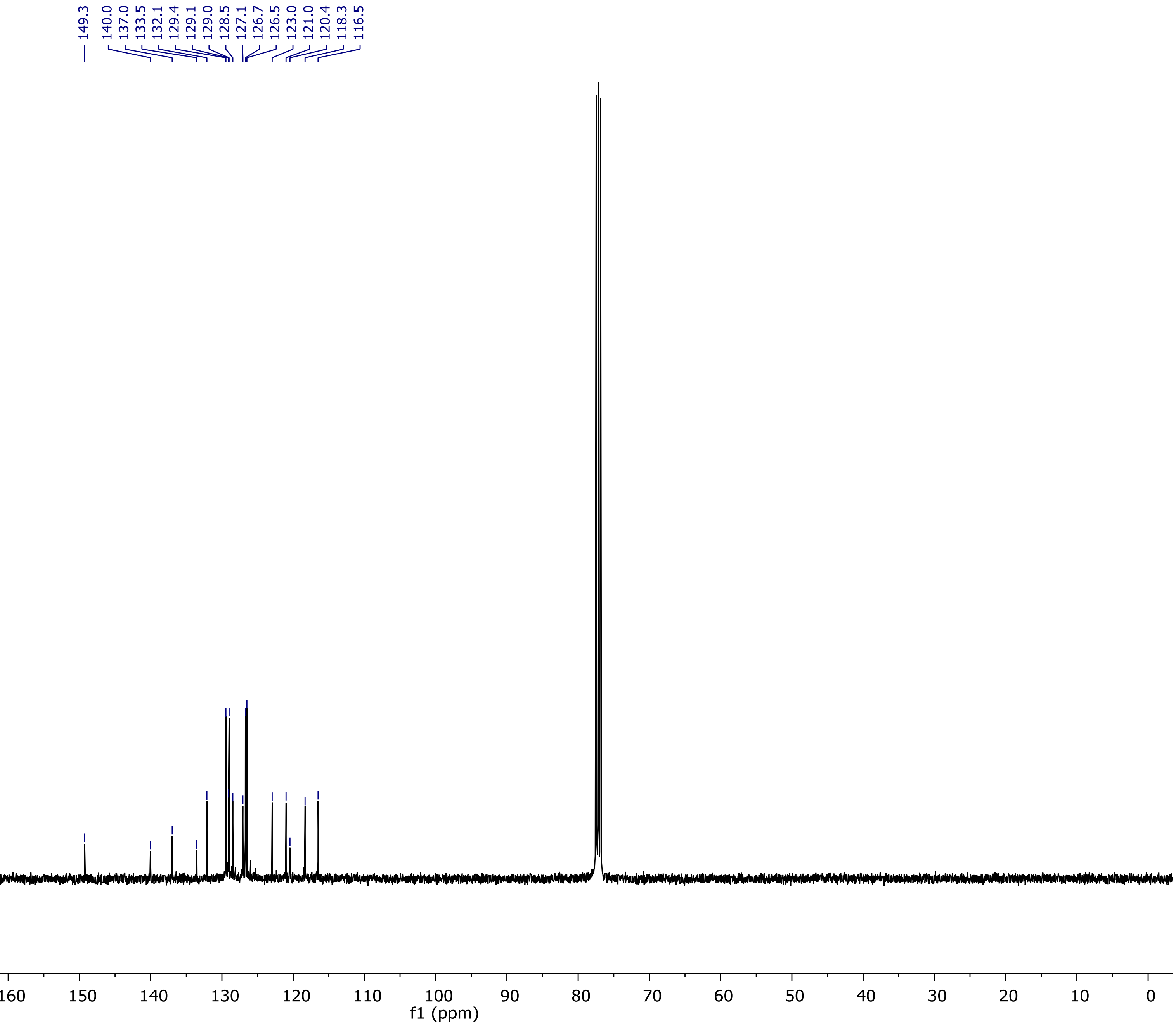

(*E*)-2-[(1,1'-biphenyl)-3-yl]-3-(pent-1-en-1-yl)-2*H*-indazole (3)

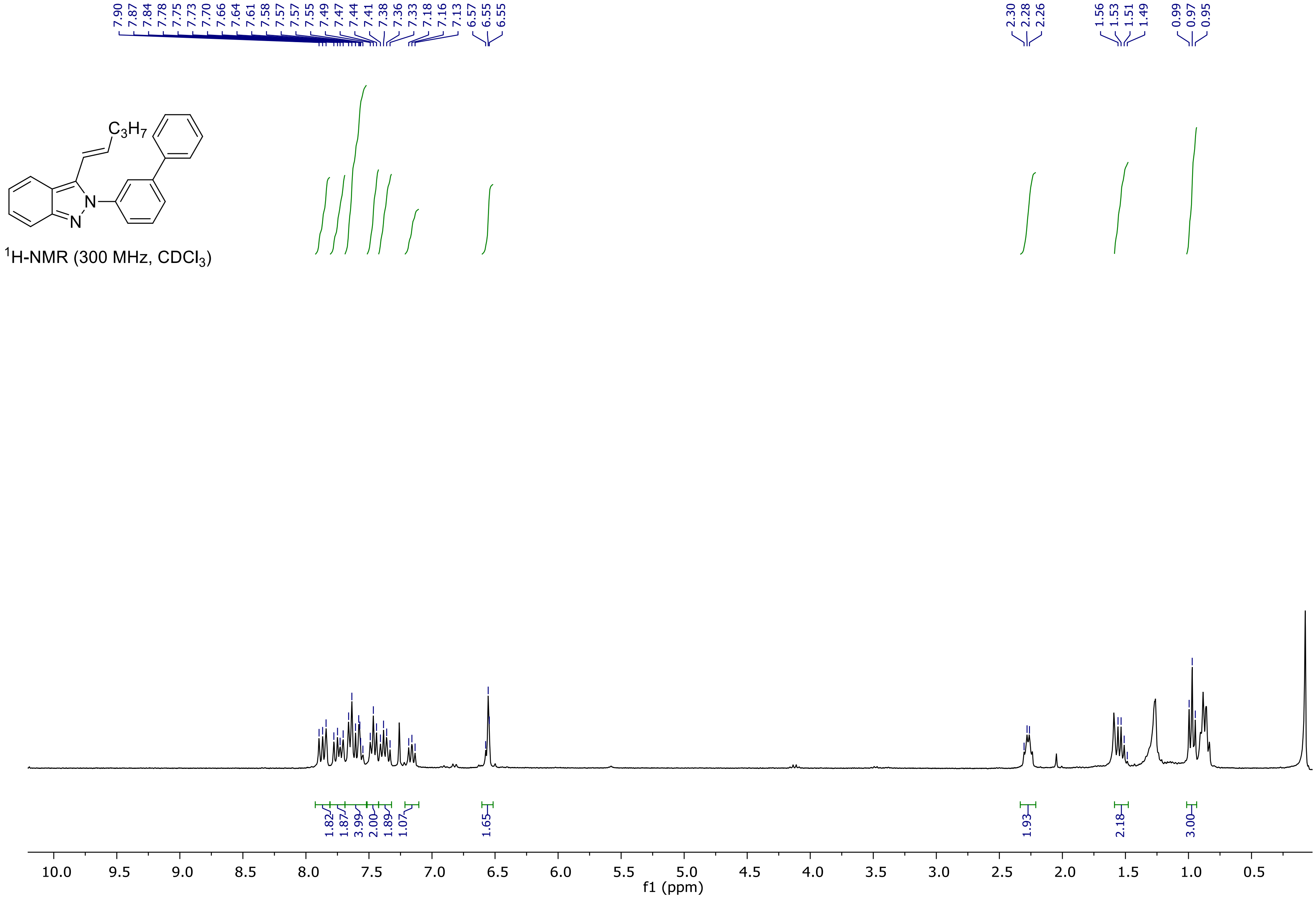

^{1}H-NMR (300 MHz, $CDCl_3$)

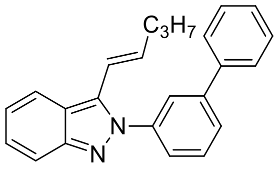

$^{13}C\{^{1}H\}$ NMR (126 MHz, $CDCl_3$)

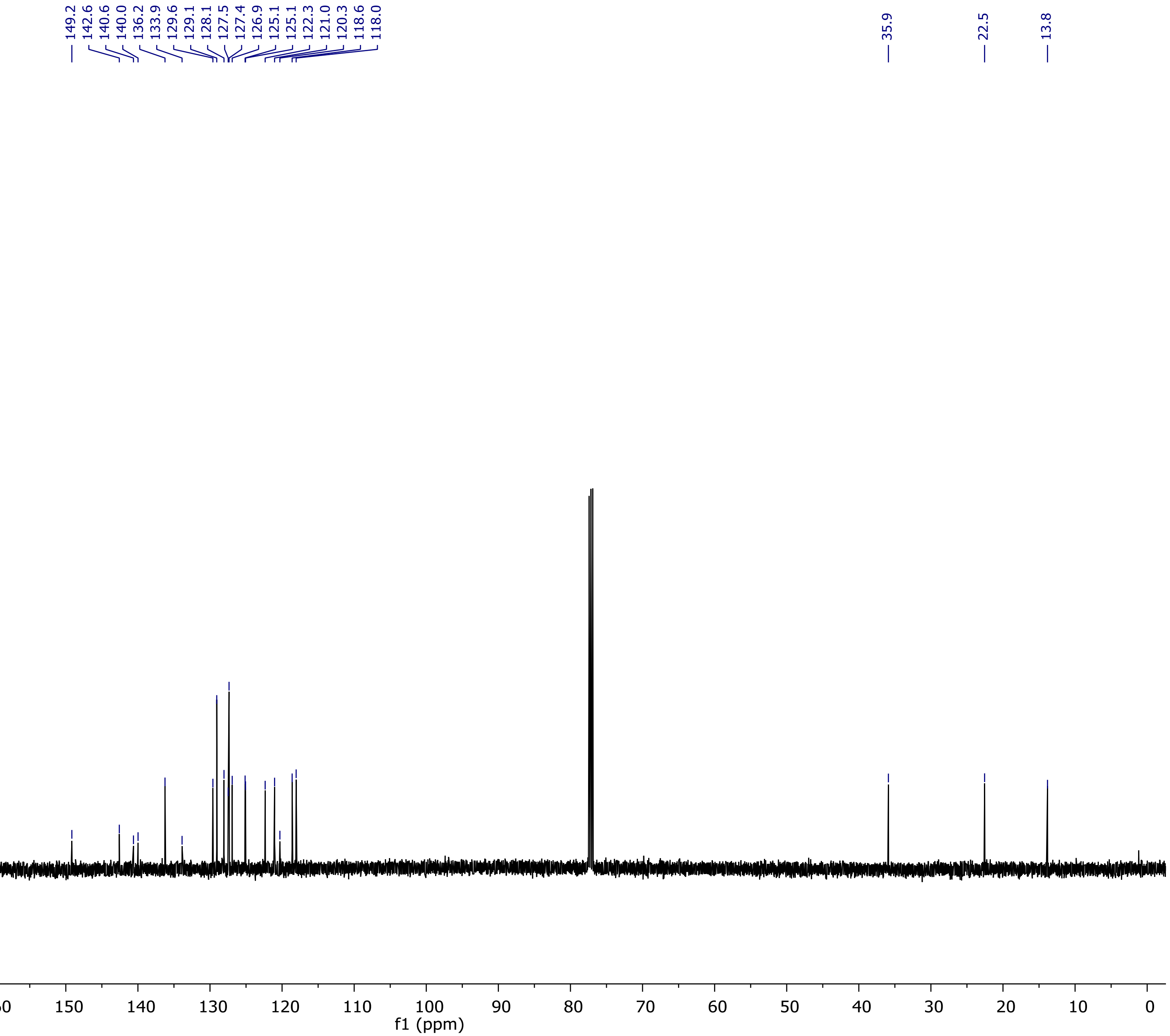

(*E*)-3-[3-(Pent-1-en-1-yl)-2*H*-indazol-2-yl]aniline (4)

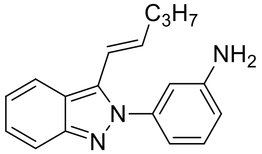

^{1}H-NMR (300 MHz, $CDCl_3$)

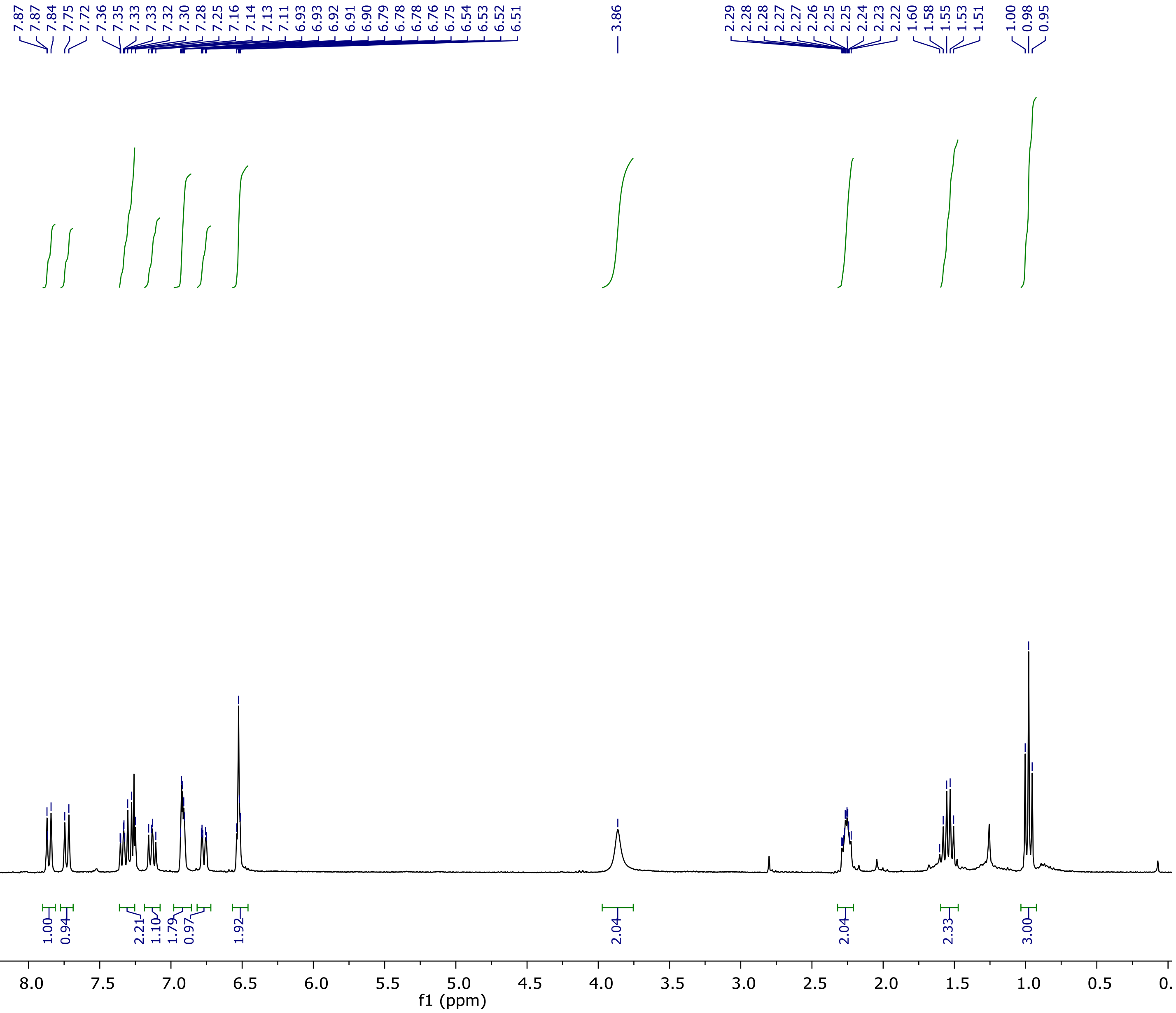

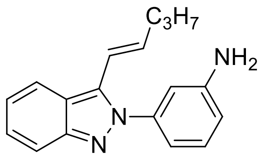

$^{13}C\ \{^1H\}$ NMR (75 MHz, $CDCl_3$)

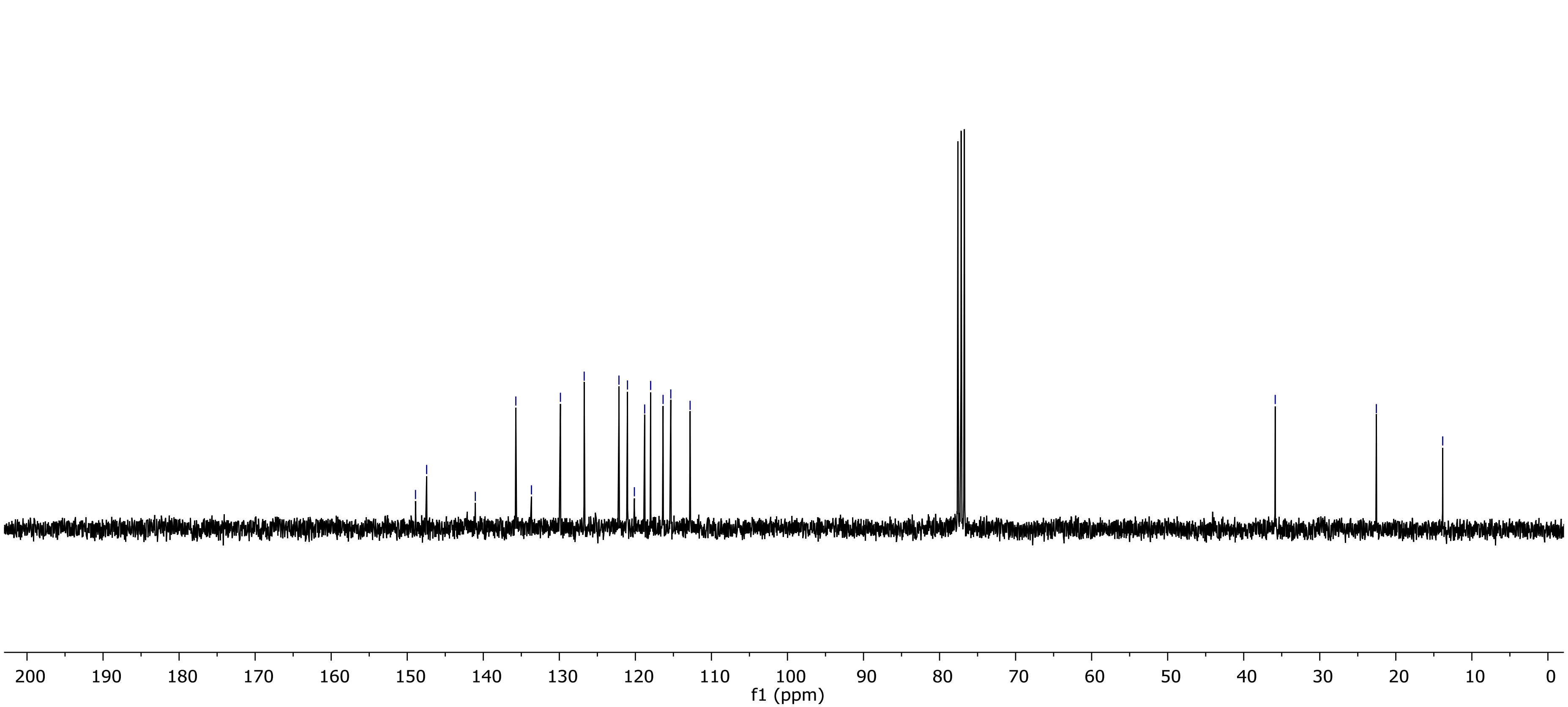

(*E*)-4-[3-(Pent-1-en-1-yl)-2*H*-indazol-2-yl]phenol (5)

^{1}H-NMR (300 MHz, $CDCl_3$)

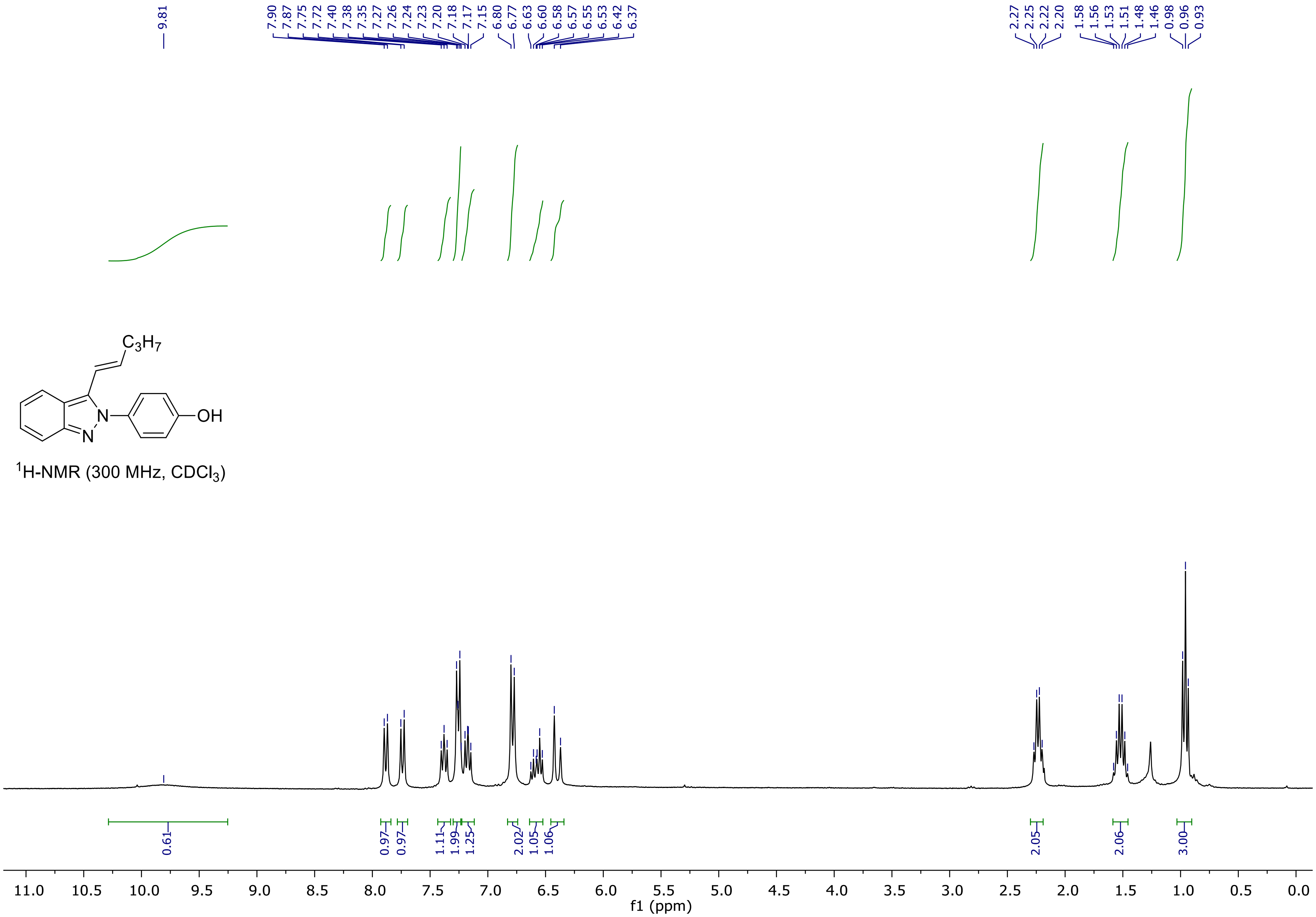

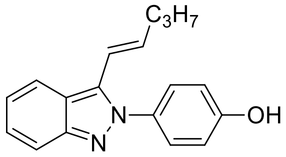

^{13}C {^{1}H} NMR (75 MHz, $CDCl_3$)

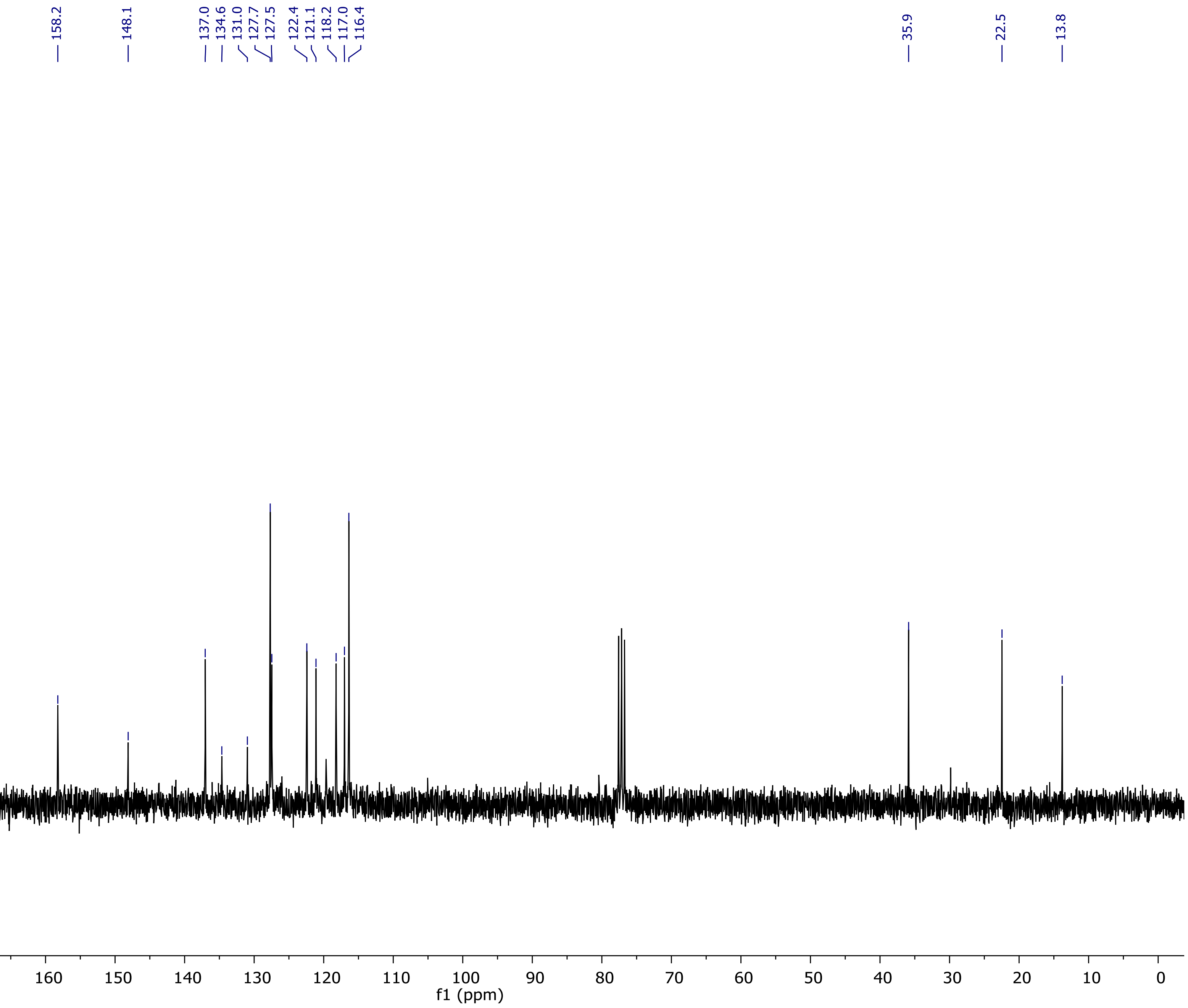

(*E*)-3-(Pent-1-en-1-yl)-2-phenyl-2*H*-indazole-5-carboxylic acid (6)

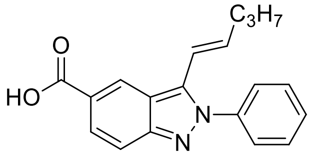

^{1}H-NMR (300 MHz, CD_3COCD_3)

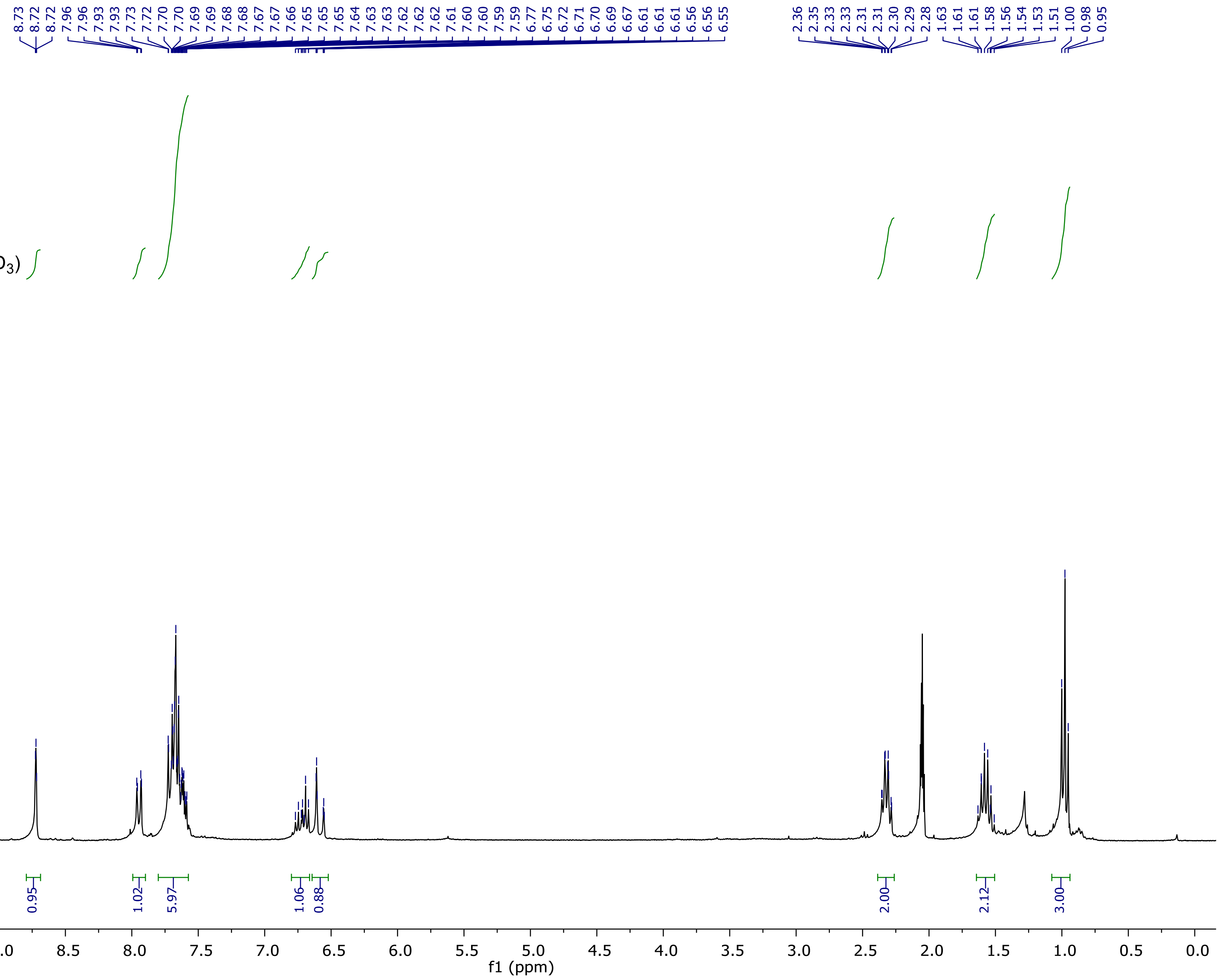

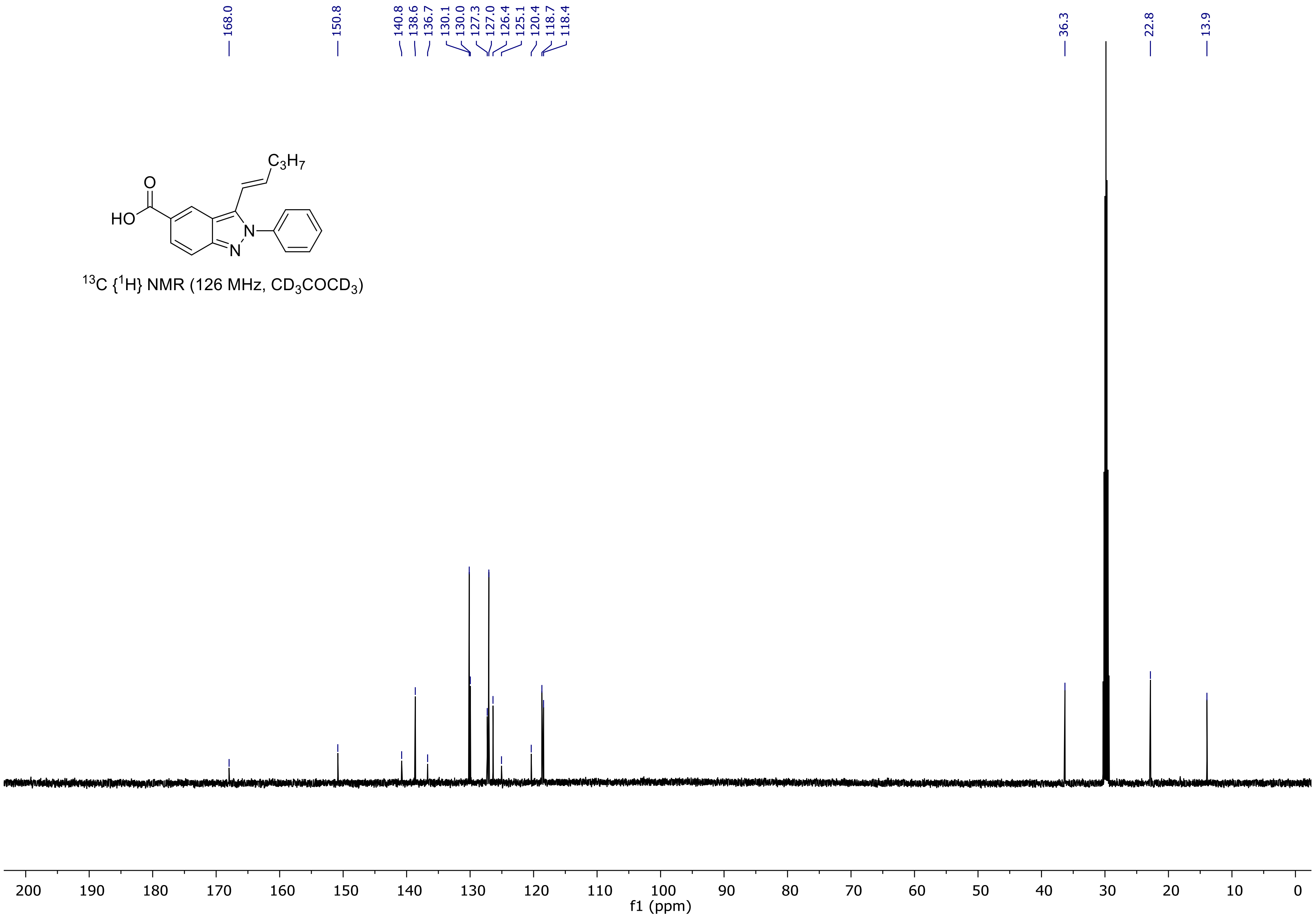

^{13}C {^{1}H} NMR (126 MHz, CD_3COCD_3)

5-Phenylindazolo[2,3-*a*]quinoline (7)

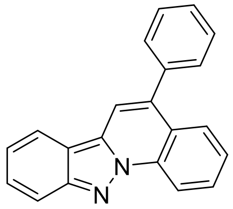

^{1}H-NMR (500 MHz, $CDCl_3$)

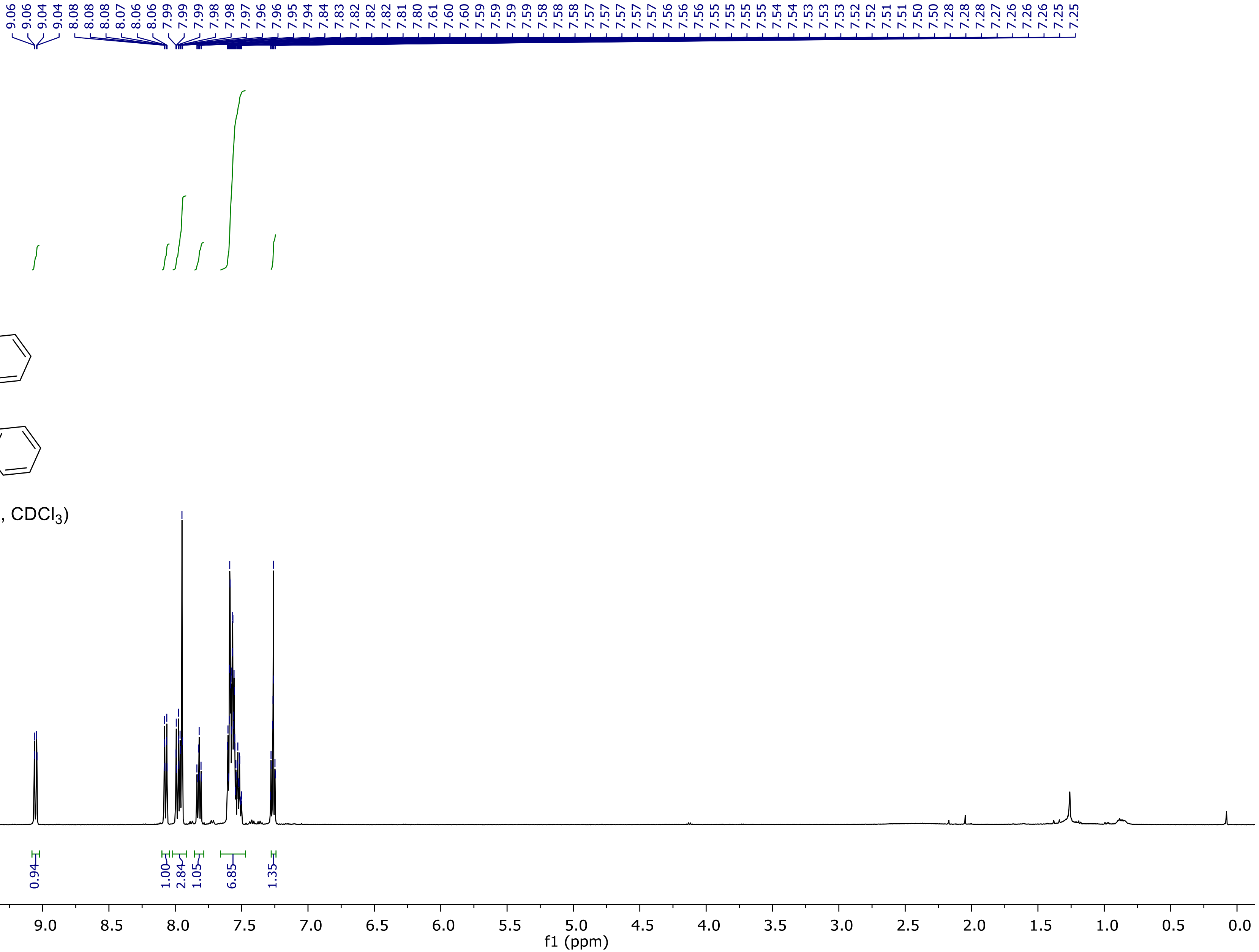

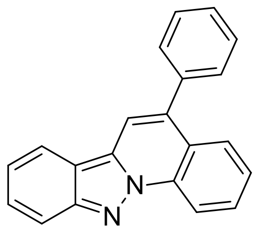

$^{13}C\ \{^1H\}$ NMR (126 MHz, $CDCl_3$)

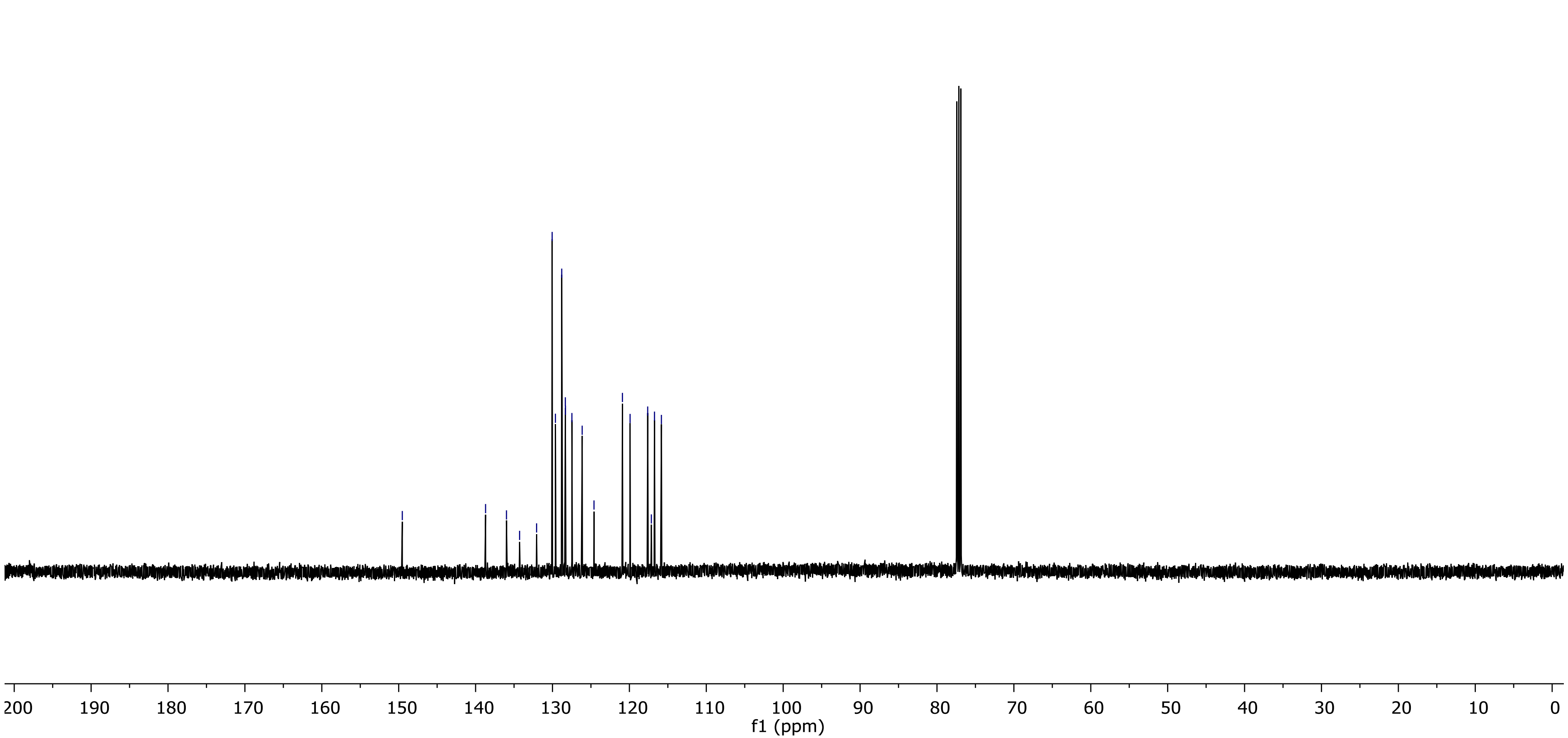

References

[1] Mañas, C.; Merino, E. Visible Light-Mediated Heterodifunctionalization of Alkynylazobenzenes for 2*H*-Indazole Synthesis. *Org. Lett.* **2024**, *26*, 1868–1873.

[2] CLH report for Copper (I) oxide, Proposal for Harmonised Classification and Labelling, ECHA, **2013**.

[3] Ying, S.; Liu, X.; Guo, T.; Li, X.; Zhou, M.; Wang, X.; Zhu, M.; Jiang, H.; Gui, Q.-W. Ultrasound-assisted bromination of indazoles at the C3 position with dibromohydantoin. *RSC Adv.* **2023**, *13*, 581–585.

[4] Souers, A. J.; Gao, J.; Brune, M.; Bush, E.; Wodka, D.; Vasudevan, A.; Judd, A. S.; Mulhern, M.; Brodjian, S.; Dayton, B.; Shapiro, R.; Hernandez, L. E.; Marsh, K. C.; Sham, H. L.; Collins, C. A.; Kym, P. R. Identification of 2-(4-Benzyloxyphenyl)-*N*-[1-(2-pyrrolidin-1-yl-ethyl)-1*H*-indazol-6-yl]acetamide, an Orally Efficacious Melanin-Concentrating Hormone Receptor 1 Antagonist for the Treatment of Obesity. *J. Med. Chem*. **2005**, *48*, 1318–1321.

[5] Boloor, A.; Kanouni, T.; Stafford, J.; Veal, J.; Wallace, M. WO 2017/161012 A1, **2017**.

[6] Sukowski, V.; Borselen, M.; Mathew, S.; Fernández-Ibáñez, M. A. S,O-Ligand Promoted *meta*-C−H Arylation of Anisole Derivatives via Palladium/Norbornene Catalysis *Angew. Chem. Int. Ed.* **2022**, *61*, e202201750.

[7] Gaussian 16, Revision C.01; Frisch, M. J. et al. Gaussian, Inc., Wallingford CT, 2016.

[8] Zhao, Y.; Truhlar, D. G. The M06 suite of density functionals for main group thermochemistry, thermochemical kinetics, noncovalent interactions, excited states, and transition elements: two new functionals and systematic testing of four M06-class functionals and 12 other functionals. *Theor. Chem. Acc.* **2008**, *120*, 215–241.

[9] Dunning Jr. T. H.; Hay, P. J. in *Modern Theoretical Chemistry*, Ed. H. F. Schaefer III, Vol. 3 (Plenum, New York, 1977) 1–28.

[10] Marenich, A. V.; Cramer, C. J.; Truhlar, D. G. Universal Solvation Model Based on Solute Electron Density and on a Continuum Model of the Solvent Defined by the Bulk Dielectric Constant and Atomic Surface Tensions. *Phys. Chem. B* **2009**, *113*, 6378–6396.
